# Nicotinamide-Based Covalent Inhibitors of tRNA (m^1^G37) Methyltransferase (TrmD) to Increase Antibacterial Activity

**DOI:** 10.1021/acsmedchemlett.6c00164

**Published:** 2026-06-10

**Authors:** Isao Masuda, Simon D. P. Baugh, Kevin McClay, Ryan J. Ford, Lily Tayag, Eric D. Strobel, Thomas Grant, Samantha A. DeSando, Hongjuan Guo, Thomas Christian, Diana Bao Hang, Zheng Ruan, John L. Kulp, Katie B. Freeman, Ya-Ming Hou, Allen B. Reitz, Dipti Kanabar

**Affiliations:** † 716094 Fox Chase Therapeutics Discovery Inc. Doylestown, Pennsylvania 18902, United States; ‡ Department of Biochemistry and Molecular Biology, 6559 Thomas Jefferson University, Philadelphia, Pennsylvania 19107, United States; § Conifer Point Pharmaceuticals, Doylestown, Pennsylvania 18902, United States

**Keywords:** TrmD (tRNA (m^1^G37) methyltransferase), Antibacterial drug discovery, Gram-negative bacteria, covalent inhibition, AdoMet (*S*-adenosyl methionine), structure-based drug design

## Abstract

Bacterial tRNA (m^1^G37) methyltransferase (TrmD) is an essential enzyme required for accurate translation and represents an attractive antibacterial target distinct from its eukaryotic counterpart, Trm5. Here, we describe the development of nicotinamide-based TrmD inhibitors incorporating electrophilic Michael-acceptor motifs to enhance cellular antibacterial activity. Mass spectrometry-based peptide mapping shows covalent modification of TrmD at Cys112, a residue proximal to the AdoMet (*S*-adenosylmethionine) binding region, providing a mechanistic basis for covalent target engagement. Consistent with this mechanism, electrophile-containing analogues showed improved antibacterial activity compared with the parent scaffold. In bacterial growth inhibition assays, selected compounds displayed greater functional specificity for TrmD-driven growth suppression relative to human Trm5, supporting preferential bacterial target engagement. Collectively, these results establish Cys112 as a chemically addressable site for covalent inhibition of TrmD and provide a foundation for the development of TrmD-directed antibacterial agents.

## Graphical abstract

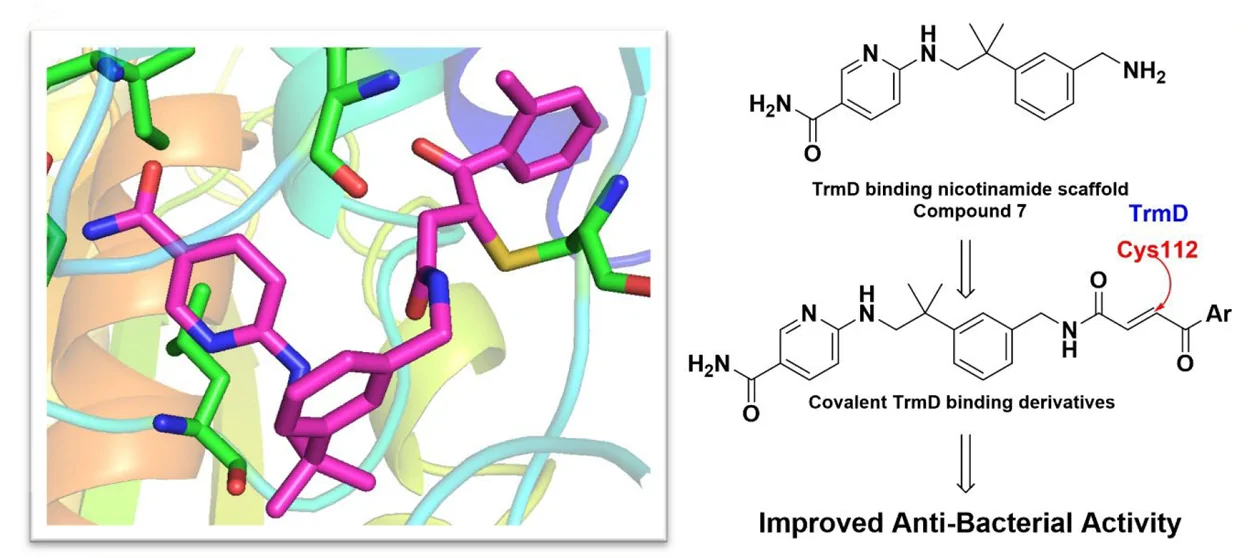

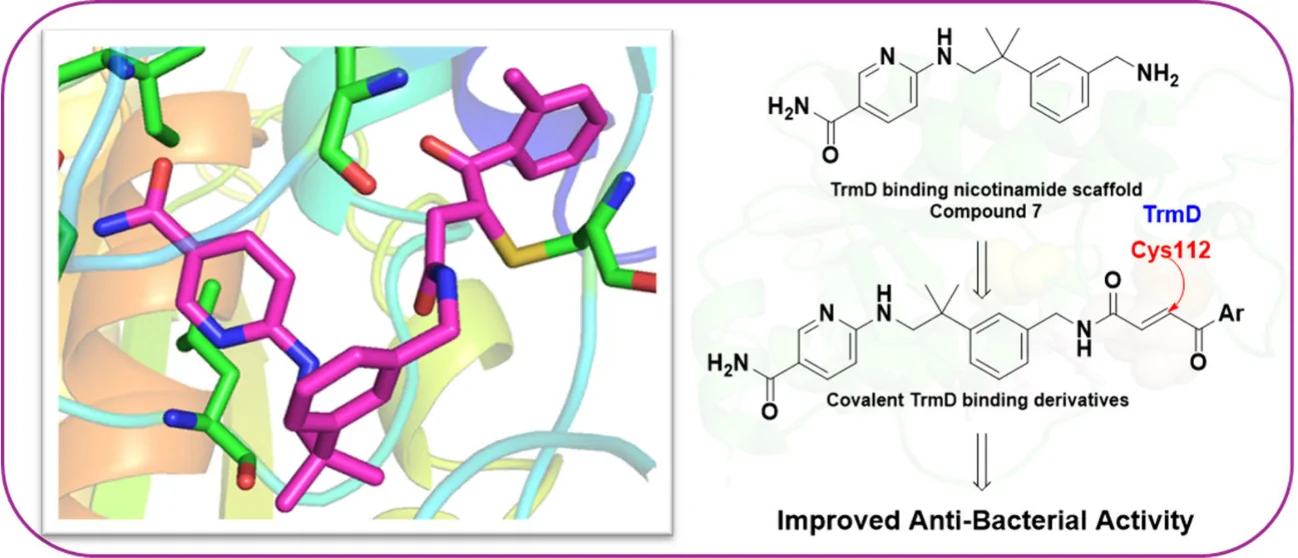

## Introduction

The rapid rise of antimicrobial resistance, particularly among Gram-negative pathogens, has intensified the need for antibacterial agents that operate through novel mechanisms and engage targets distinct from those exploited by current antibiotics.^1–4^ Gram-negative bacteria pose an additional challenge because the outer-membrane permeability barrier and limited intracellular accumulation often restrict access to essential cytosolic targets, accelerating therapeutic failure as resistance emerges.^5–8^ Despite these challenges, there is a strong interest in essential bacterial enzymes that support core processes such as translation, for which disrupting protein synthesis remains a validated strategy for antibacterial therapy.^9,10^

tRNA (m^1^G37) methyltransferase (TrmD), a member of the SpoU-TrmD (SPOUT) RNA methyltransferase family, catalyzes transfer of a methyl group from *S*-adenosyl methionine (AdoMet) to the *N* ^*1*^ position of guanosine 37 in bacterial tRNA.^11–13^ This modification occurs immediately 3′ adjacent to the anticodon and is critical for charging tRNAPro and tRNAArg and for maintaining the reading frame,^14,15^ limiting frameshift errors that would otherwise lead to truncated, nonfunctional proteins.^12,13^ TrmD is essential for growth in diverse bacterial species, supporting its potential as a broad spectrum antibacterial target.^16–19^ Importantly, TrmD is structurally and mechanistically distinct from its eukaryotic counterpart Trm5. For example, TrmD and Trm5 evolved with distinct structures and share no sequence or structural homology. While TrmD uses the trefoil-knot fold to perform catalysis, Trm5 uses the Rossman-fold; while Trm5 recognizes only the anticodon-stem loop for tRNA binding, Trm5 requires the entire L-shaped tRNA tertiary structure; while TrmD requires the divalent metal ion Mg^2+^ for methyl transfer, Trm5 does not; and while TrmD binds the methyl donor AdoMet with high stringency of discrimination, Trm5 binds with a much lower stringency.^20–28^ These fundamental structural and mechanistic differences of TrmD from Trm5 create an opportunity to develop selective inhibitors that minimize human off-target activity.^29^ While multiple TrmD binding small molecules have been reported, including fragment derived and structure guided series that engage the AdoMet binding region, these reversible ligands have shown limited or inconsistent antibacterial activity, highlighting the gap between biochemical engagement and Gram-negative bacterial growth inhibition.^19,30–32^

Covalent inhibitor design offers a complementary strategy to enhance target engagement and the durability of inhibition by forming an irreversible or slowly reversible bond with a nucleophilic residue positioned within or proximal to the active site. When carefully designed, covalent inhibitors can provide improved potency, prolonged residence time, and enhanced cellular efficacy while maintaining selectivity through electrophile tuning and target site accessibility.^33–37^ The catalytic site of TrmD accommodates AdoMet and positions the tRNA substrate for methyl transfer via a highly conserved geometry^11^ Figure 1. Structural analyses reveal that a TrmD binding small molecule containing a nicotinamide scaffold can bind within the AdoMet binding pocket^19^ (Figure 1). This positioning allows the electrophilic functional groups on the nicotinamide derivatives to approach the reactive side chains, creating an opportunity for covalent bond formation. Among the proximal residues around the AdoMet binding pocket, Cys112 is particularly well suited for covalent targeting due to its nucleophilic thiol side chain and spatial orientation toward the bound scaffold. By covalently engaging Cys112, nicotinamide-based inhibitors can potentially block AdoMet binding and tRNA methylation, thereby enhancing the potency and prolonging target engagement. This strategy leverages both the scaffold’s structural complementarity and the chemical reactivity of the cysteine residue, providing a rational approach to designing selective covalent TrmD inhibitors with potential antibacterial activity. Building upon previously reported nicotinamide-based scaffolds, which provide an electrophilic handle for covalent modification, we designed and optimized fragments to engage nucleophilic residues within the TrmD active site. Mass spectrometry and biophysical/biochemical assays confirmed the formation of a covalent bond, functional inhibition, and selectivity compared to human Trm5.

**Figure 1:**
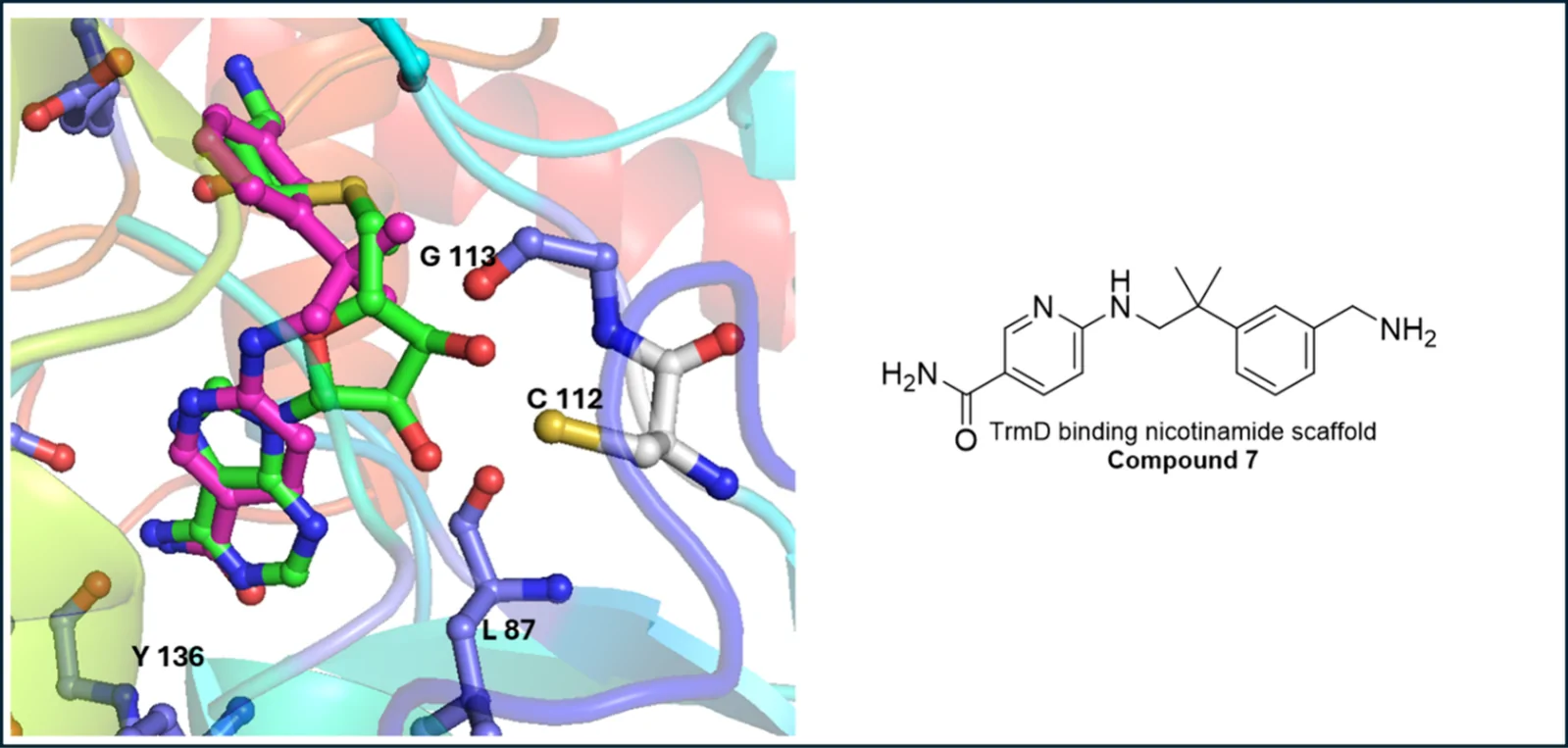
Binding pose of the nicotinamide-scaffold compound **7** (magenta) in the TrmD (PDB: 4YQT) active site. Compound **7** occupies the AdoMet (*S*-adenosyl methionine) (green) binding pocket and is positioned in close proximity to the Cys112 residue, supporting its potential for covalent interaction.

## Results and Discussion

### Chemistry

A modified covalent handle has been added to a nicotinamide-containing TrmD binding^19^ scaffold and was synthesized as outlined in Scheme 1. Functionalization of the core scaffold proceeded through stepwise derivatization to enable late-stage diversification. Base-promoted α-alkylation of aryl acetonitrile **1** afforded intermediate **2**, which was subsequently subjected to benzylic bromination using *N*-bromosuccinimide (NBS) to provide bromomethyl intermediate **3**. Protecting group manipulations were employed as necessary to enable chemoselective transformations. Intermediate **3** underwent nucleophilic substitution, followed by deprotection and selective reprotection to furnish the Boc-protected intermediate **4**. Reduction of the nitrile group in **4** using Raney nickel delivered primary amine **5**, which was coupled to 6-fluoropyridine-3-carbonitrile via nucleophilic aromatic substitution to afford intermediate **6** in moderate to good yield. The oxidative conversion of the heteroaryl nitrile to the corresponding carboxamide, followed by deprotection, yielded the advanced intermediate **7**.^19^ Late-stage diversification was achieved by amide coupling of the terminal amine in **7** with a panel of commercially available or prepared via Knoevenagel condensation of substituted phenyl ethanone and glyoxylic acid, yielding the final compound series (**8**–**37**) and enabling rapid access to structural diversity for SAR evaluation. The synthesis of compounds containing various linkers (**38**–**43**) is shown in the Supporting Information ().

**Scheme 1:**
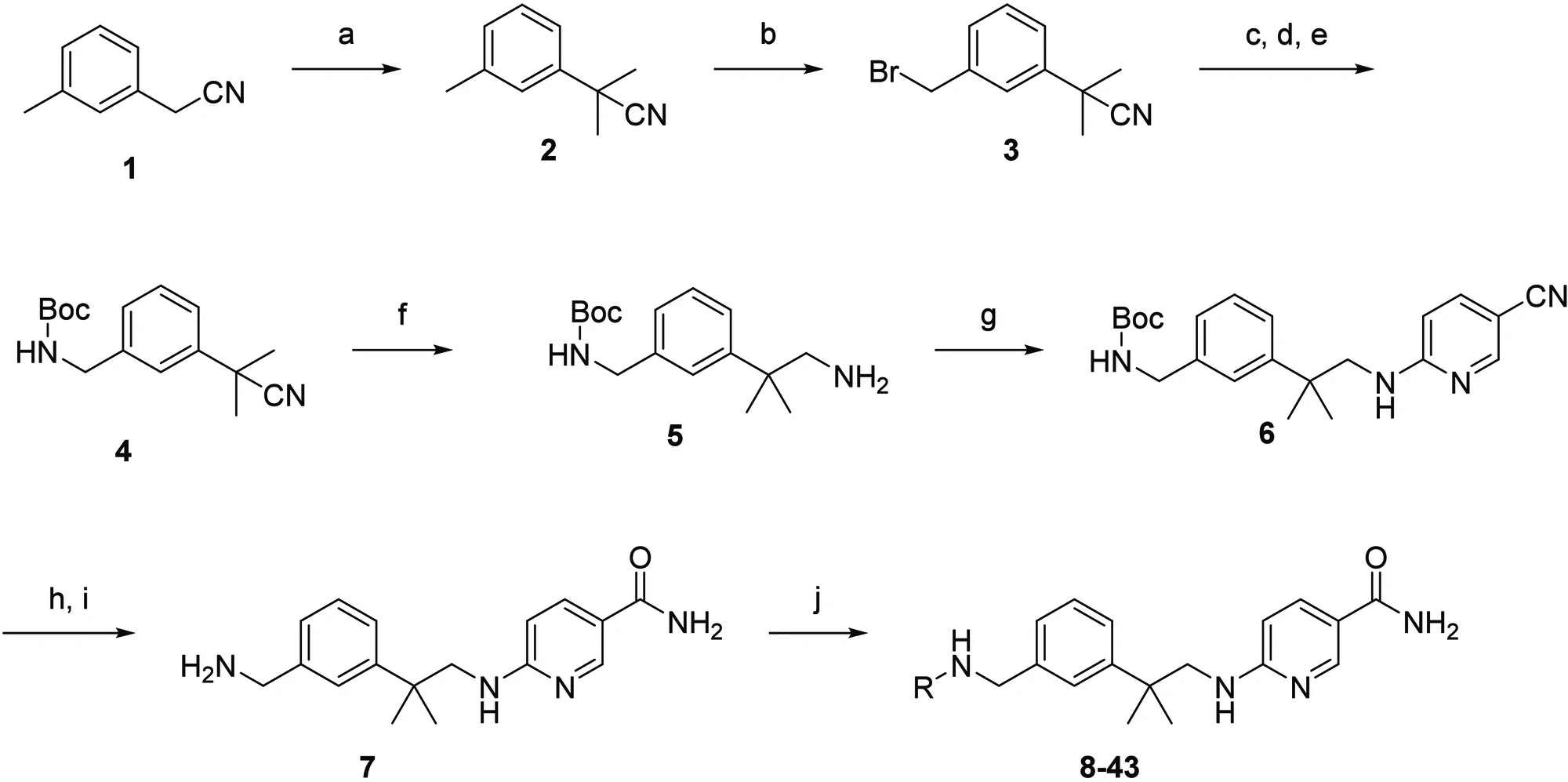
Synthesis of Modified Covalent Handle Added to a Nicotinamide-Containing TrmD Binding Scaffold^a^

a Reagents: (a) CH_3_I, NaH, Dry THF, RT, overnight; (b) NBS, Benzoyl peroxide, CCl_4_, Reflux, 16 h; (c) (Boc)_2_NH, NaH, THF, RT, 24h; (d) 4 M HCl in dioxane, DCM, RT, overnight; (e) di*tert*-butyl dicarbonate, TEA, DCM, RT, overnight; (f) RaNi/H_2_, EtOH, RT, overnight; (g)), 6-fluoropyridine-3-carbonitrile, TEA, DMF, 80 °C, overnight; (h) 30% aq. H_2_O_2_, K_2_CO_3_, DMSO, 80 °C, 4h; (i) 4 M HCl in dioxane, DCM, RT, 2 h; (j) Carboxylic acid derivative, HATU, DIPEA, DMF, RT, overnight.

To elucidate the relationship between target engagement and antibacterial activity, compounds **7**–**43** were evaluated for TrmD binding by differential scanning fluorimetry, reported as changes in melting temperature from DMSO control (Δ*T*_m_), and for cellular efficacy using percent growth inhibition at 100 μM and MIC determination in *Escherichia coli* (*E. coli*) porin mutant strain, which is deficient in the permeability of the Gram-negative double-membrane envelope structure^20^ (Table 1). Compounds exhibiting ≤50% growth inhibition at 100 μM were not advanced to MIC testing. The unsubstituted parent compound **7** produced only a small thermal shift (Δ*T*_m_ = 2.5 °C) and moderate growth inhibition (46%), indicating weak target engagement and limited cellular activity. Early acylated analogs **8** and **9** showed minimal thermal shifts (Δ*T*_m_ ≤ 1.5 °C) and no detectable growth inhibition. In contrast, compound **10** produced a larger thermal shift (Δ*T*_m_ = 8.8 °C) but still lacked antibacterial activity, indicating that binding alone does not translate into cellular efficacy.

**Table 1:**
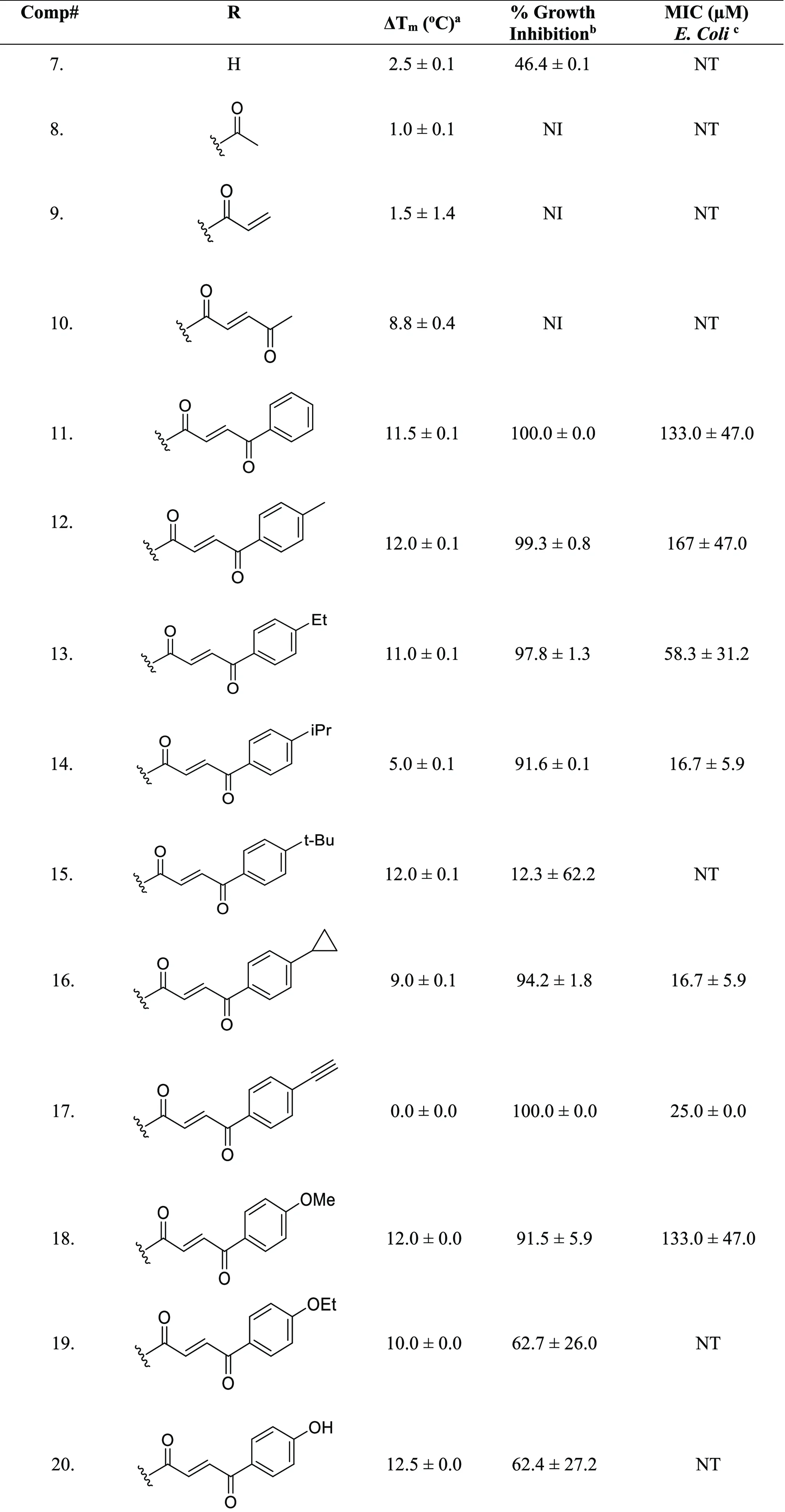

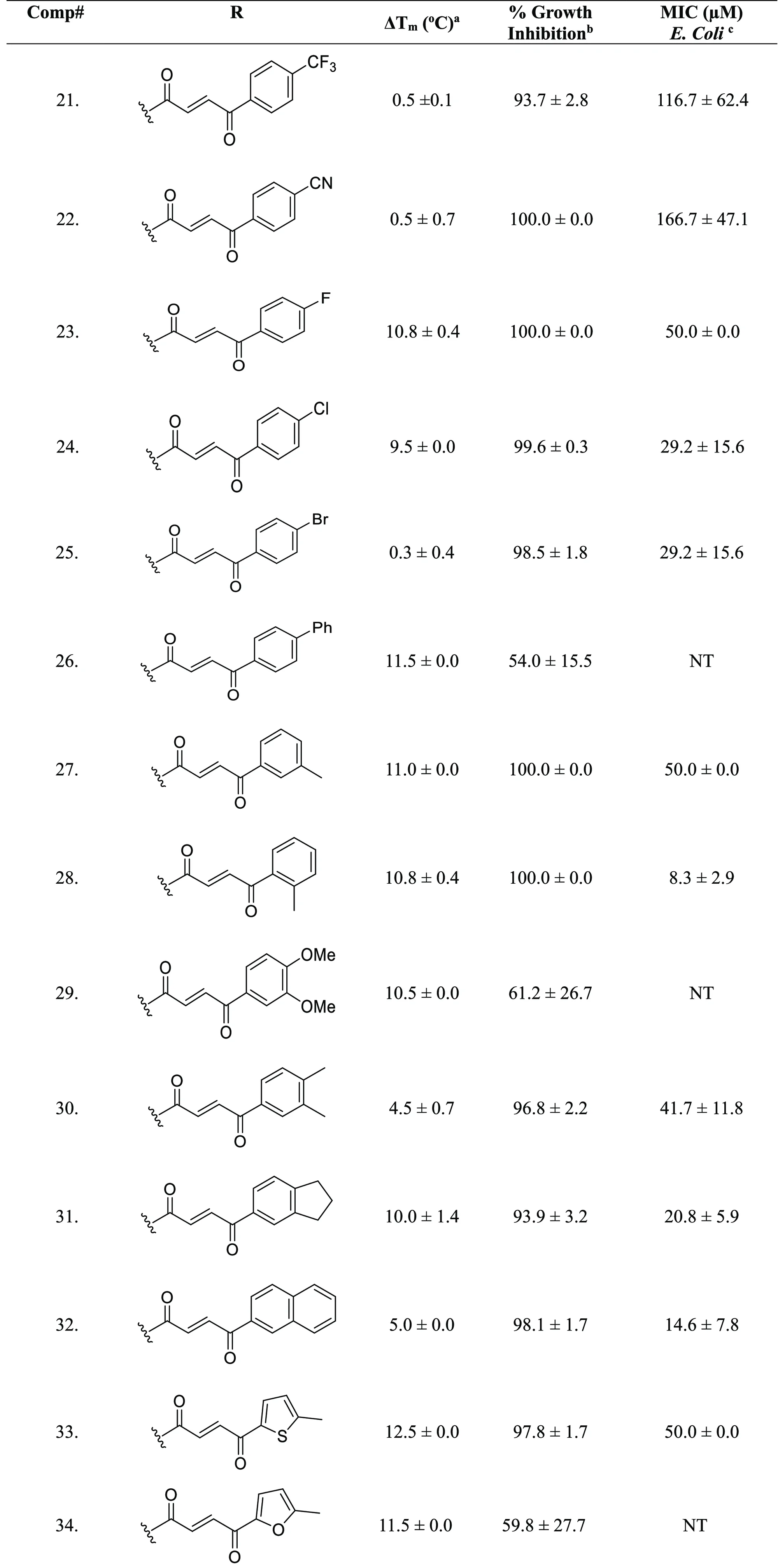

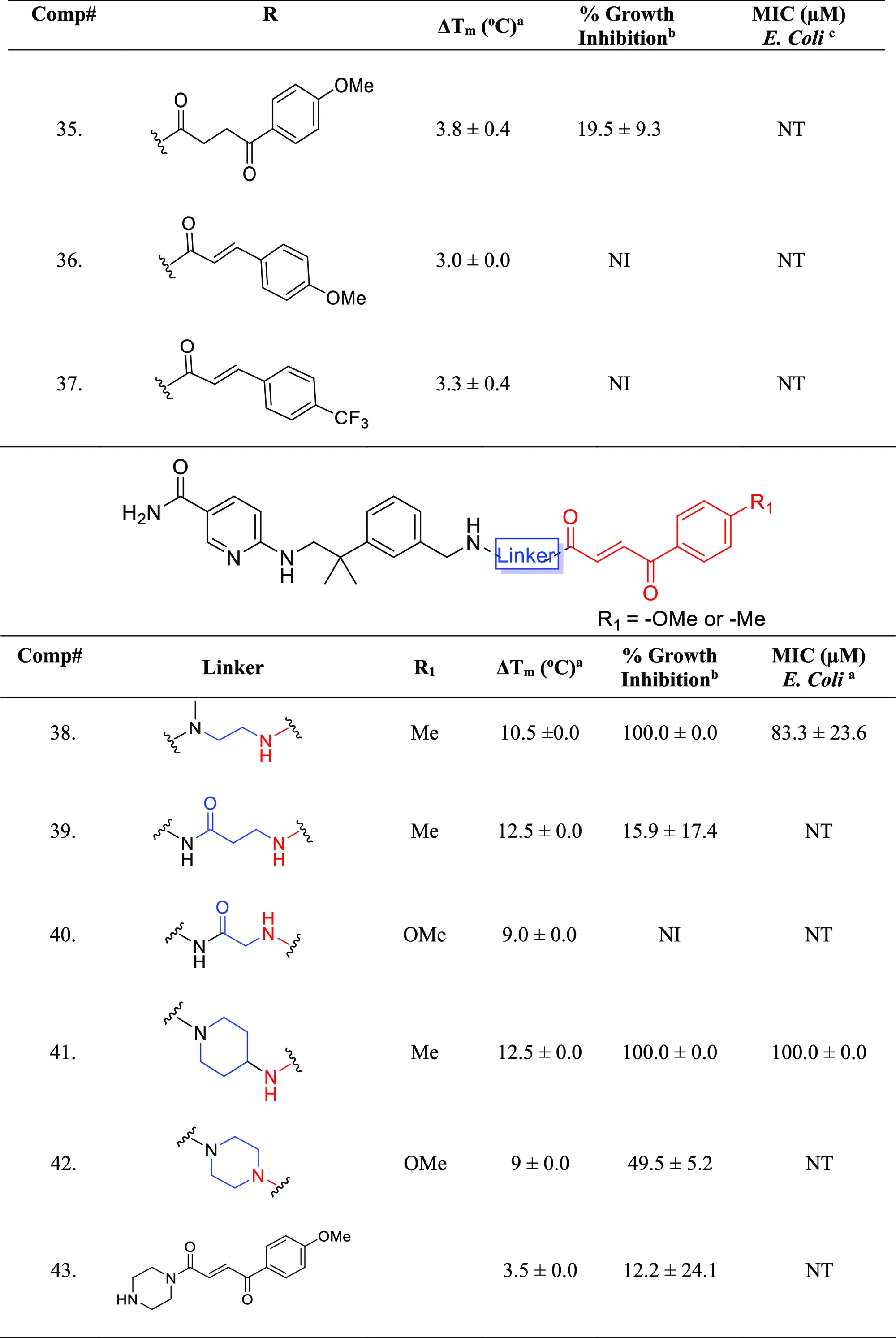
Structure–Activity Relationship (SAR) for Nicotinamide-Based TrmD Inhibitors (Compounds **7**–**43**) Bearing Varied Aryl/Michael Acceptor Substituents/Varied Linker^a^

* TrmD engagement was assessed by differential scanning fluorimetry (Δ*T*_m_, °C), and whole-cell activity was evaluated by *E. coli* porin mutant strain growth inhibition (%) and minimum inhibitory concentration (MIC, μM) as indicated.

a Δ*T*_m_ values were determined by differential scanning fluorimetry performed in triplicate with compounds tested at 10 μM

b Percent growth inhibition was determined at 100 μM following incubation with an *E. coli* porin mutant strain, which was engineered to express a mutant porin protein that enhances uptake of chemicals from the outside. Quantification of growth inhibition by colony-forming units relative to vehicle controls.

c MIC values were determined by overnight incubation of compounds at varying concentrations with an *E. coli* porin mutant strain, which was engineered to express a mutant porin protein that enhances uptake of chemicals from the outside.

NI = No inhibition (0% inhibition) at 100 μM.

NT = Compounds with ≤50% inhibition at 100 μM were not tested for MIC.

Introduction of an aryl-substituted α,β-unsaturated carbonyl (Michael acceptor) motif (**11–33**) generally produced larger thermal shifts and enabled measurable antibacterial activity in multiple cases, consistent with improved target engagement within this structural class. The unsubstituted phenyl enone **11** exhibited a pronounced thermal shift (Δ*T*_m_ = 11.5 °C) and complete growth inhibition, translating into moderate antibacterial potency (MIC = 133 μM). The *para*-methyl analog **12** displayed comparable thermal shift (Δ*T*_m_ = 12.0 °C) but did not improve the MIC (167 μM). The *para*-ethyl analog **13** retained a large thermal shift (Δ*T*_m_ = 11.0 °C) and showed improved potency (MIC = 58 μM), while increasing steric bulk with a *p*ara-isopropyl substituent (**14**) preserved a moderate thermal response and delivered a marked gain in antibacterial activity (MIC = 16.7 μM). Similarly, the *para*-cyclopropyl analog **16** exhibited an improved thermal shift and matched this potency (MIC = 16.7 μM), indicating that compact hydrophobic *para*-substituents are optimal within this series. In contrast, incorporation of a linear *p*-alkyne substituent in compound **17** did not produce a measurable thermal shift (Δ*T*_m_ = 0.0 °C), despite high growth inhibition at 100 μM, suggesting that this observed cellular effect is not driven by strong TrmD binding.

Substituent electronics further influenced activity. Electron-donating substituents such as methoxy (**18**) and ethoxy (**19**) produced large thermal shifts (Δ*T*_m_ ≈ 10–12 °C) but did not improve antibacterial potency, while the polar phenol analog **20** similarly failed to enhance activity. In contrast, strongly electron-withdrawing substituents (**21**, *p*-CF_3_; **22**, *p*-CN) resulted in smaller thermal shifts (Δ*T*_m_ ≤ 0.5 °C) and weaker antibacterial activity (MIC ≥ 100 μM), indicating that excessive electronic perturbation is poorly tolerated. Halogen substitution at **23** and **24** maintained relatively large thermal shifts; the bromo-substituted analog **25** exhibited a small thermal shift (Δ*T*_m_ = 0.3 °C) yet retained high growth inhibition, achieving MIC values in the 29–50 μM range. However, the bulky biphenyl analog **26**, despite producing a large thermal shift (Δ*T*_m_ = 11.5 °C), lacked antibacterial activity, consistent with excessive steric demand limiting productive whole-cell translation. Substituent position and aryl disubstitution further modulated activity. The *meta*-methyl analog **27** produced a large thermal shift (Δ*T*_m_ = 11.0 °C) but translated into only moderate antibacterial potency (MIC = 50 μM), whereas the *ortho*-methyl analog **28** maintained a comparable thermal shift (Δ*T*_m_ = 10.8 °C) while delivering a pronounced improvement in cellular activity (MIC = 8.3 μM), consistent with a more favorable binding geometry enabled by ortho substitution. In contrast, aryl disubstitution and increased ring fusion were generally less productive. The dimethoxy analog **29** showed a slightly reduced thermal shift (Δ*T*_m_ = 10.5 °C) and poor antibacterial activity, while the dimethyl analog **30** retained a measurable thermal response but exhibited moderate potency (MIC = 41.7 μM). Likewise, the bicyclic hydrophobic analogs **31** and **32** maintain measurable thermal shifts and showed growth inhibition, indicating that increased aromatic bulk and ring fusion are tolerated and likely productive for engagement and cellular translation. Heteroaryl replacement further emphasized the need for a balance between hydrophobicity and polarity. The thienyl analog **33** exhibited a large thermal shift (Δ*T*_m_ = 12.5 °C) and moderate antibacterial potency (MIC = 50 μM), whereas the more polar oxygen containing heteroaryl analog **34** showed a similar thermal shift but poor antibacterial activity, suggesting limited cellular translation.

Integrity of the electrophilic pharmacophore was required for activity. Compound **35**, which lacks the Michael acceptor functionality, exhibited reduced thermal shift and antibacterial activity, indicating that removal of the electrophilic handle compromises productive engagement. Similarly, compounds **36** and **37**, which retain only a cinnamoyl (cinnamide like) mono carbonyl motif, produced weak thermal shifts (Δ*T*_m_ = 3.0–3.3 °C) and were inactive in the cellular assay. Overall, this data supports the α,β-unsaturated 1,3-dicarbonyl motif as a key determinant of TrmD engagement and antibacterial activity.

To probe linker effects while holding the warhead constant, analogs **38**–**42** retained the *para*-methyl Michael acceptor motif of **12** or **18** and varied the linker connecting the nicotinamide scaffold to the electrophilic handle. Several variants maintained large thermal shifts (up to Δ*T*_m_ ≈ 12.5 °C), indicating that target engagement can be preserved despite substantial changes to the linker; however, these modifications did not consistently improve antibacterial potency. For example, the acyclic diamine analog **38** showed a large thermal shift (Δ*T*_m_ = 10.5 °C) but only modest activity (MIC = 83 μM), while more polar amide containing linkers (**39**–**40**) retained favorable thermal shifts without improving cellular efficacy. Constrained cyclic diamine variants (**41**–**42**) also maintained large thermal shifts (Δ*T*_m_ ≈ 12.5 °C) but delivered limited potency gains (e.g., MIC = 100 μM), highlighting that linker modifications can preserve binding while altering physicochemical properties that limit antibacterial activity.

Finally, we established that both the Michael acceptor warhead and the nicotinamide-based TrmD binding scaffold were required for antibacterial activity. Compound **43**, which retains an electrophilic covalent handle but lacks the nicotinamide recognition element, failed to improve the thermal shift or antibacterial performance, demonstrating that the electrophile alone is insufficient to drive TrmD engagement or growth inhibition.This supports the conclusion that cell-based activity in this series is primarily driven by TrmD-directed inhibition, where the scaffold positions the electrophile for productive engagement within the active site.

As shown in Table 2 antibacterial activity of compound **7** against Gram-positive and Gram-negative bacteria was not measurable (MIC > 800 μM) across all tested strains. In contrast, compound **28** displayed potent activity against multiple *Staphylococcus aureus* strains, including a methicillin-sensitive strain (MSSA), methicillin-resistant strains (MRSA), and an enterotoxin producer strain (MIC = 1.56–6.25 μM) and moderate activity against additional Gram-negative organisms, including *Haemophilus influenzae* and *Vibrio cholerae* (MIC = 50 μM). These results indicate that incorporation of the covalent electrophilic motif markedly improves antibacterial activity relative to the parent scaffold.

**Table 2:** Minimum Inhibitory Concentrations (MICs) of Compounds **7** and **28** against Representative Gram-Positive and Gram-Negative Bacterial Strains

Bacteria	Strain	MIC (μM)^a^ Compound 7	MIC (μM)^a^ Compound 28
*S. aureus*	Newman	>800	3.12
	M1198	>800	3.12
	F336222	>800	3.12
	SU-5	>800	1.56
	TCH60	>800	6.25
	FRI361	>800	3.12
*H. influenzae*	Rd KW20	>800	50.0
*V. cholerae*	TND0905	>800	50.0

a MIC values were determined in triplicate.

In summary, aryl-substituted α,β-unsaturated carbonyl (Michael acceptor) analogs gave the largest thermal shifts and the most measurable antibacterial activity, while strongly electron-donating or electron-withdrawing substituents reduced potency. Removing the Michael acceptor **(35**) or replacing it with a monocarbonyl motif (**36**, **37**) abolished activity and removing the nicotinamide scaffold (**43**) also eliminated engagement. The unsubstituted parent (**7**) and nonelectrophilic acyl analogs (**8**–**10**) were likewise inactive in cells despite measurable binding, reinforcing that both the electrophilic warhead and the nicotinamide recognition element are required. Across the series, Δ*T*_m_, growth inhibition, and MIC were not always aligned, even in the porin mutant *E. coli* background indicating that binding to TrmD alone is insufficient to predict functional inhibition in cells and likely reflects binding modes that do not fully block catalysis. Together, these results guided optimization to compound **28** as the lead analog and define the structural features required for productive covalent engagement of TrmD.

## Mechanism of Action

Motivated by prior reports from the Nomura group demonstrating that electrophile-containing small molecules can function as molecular glues in mammalian systems by covalently recruiting E3 ligases to induce target degradation of targeted proteins,^38^ we tested **18**, bearing a *para*-methoxy α,β-unsaturated cinnamate motif for its ability to serve as a molecular glue. We initially hypothesized that incorporation of a covalent handle into the nicotinamide based TrmD binder might enhance antibacterial activity by promoting TrmD degradation via recruitment of bacterial E3 ligases or components of bacterial proteolysis machinery.^39,40^ To test this hypothesis, we performed proteomic profiling using the biotinylated analog of **18**. This biotinylated compound retained activity of the parent **18** and showed 91.5% *E. coli* cell growth inhibition at 100 μM; however, these studies did not reveal selective engagement of proteins associated with the bacterial degradation machinery nor did they provide evidence of interactions with E3 like ligases or other proteolytic components (). Consistent with these findings, immunoblot analyses showed no detectable reduction in cellular TrmD protein levels following compound treatment, indicating that antibacterial activity was not driven by induced TrmD degradation. Instead, the pull-down experiment showed that TrmD itself was the primary engaged protein across the proteomic data set. Moreover, qPCR analysis of **18** () revealed dose-dependent upregulation of *TrmD* transcripts at higher concentrations, consistent with a compensatory cellular response to TrmD inhibition rather than protein degradation, suggesting that the antibacterial activity was not driven by a molecular glue-mediated degradation mechanism.

To define the molecular basis of TrmD engagement, LC-MS/MS analysis was performed following incubation of representative electrophile containing compound **28** (MIC = 8.3 μM) with *E. coli* cell lysates. After trypsin digestion these studies identified a TrmD derived peptide containing Cys112 (LILVCGR) bearing a mass shift consistent with covalent inhibitor containing compound **28** attachment (Figure 2) with MS/MS fragment ion coverage supporting localization of the modification to the cysteine containing region (). Cys112 is positioned adjacent to the AdoMet binding pocket, providing a structural rationale as to how covalent capture at this site could inhibit the protein and disrupt cofactor-dependent catalysis. Similar results implicating Cys112 as a site of covalent attachment were obtained for compounds **12** and **27**. Importantly, detection of TrmD labeling within a complex bacterial proteome () supports TrmD target engagement as the driver of selective growth inhibition.

**Figure 2:**
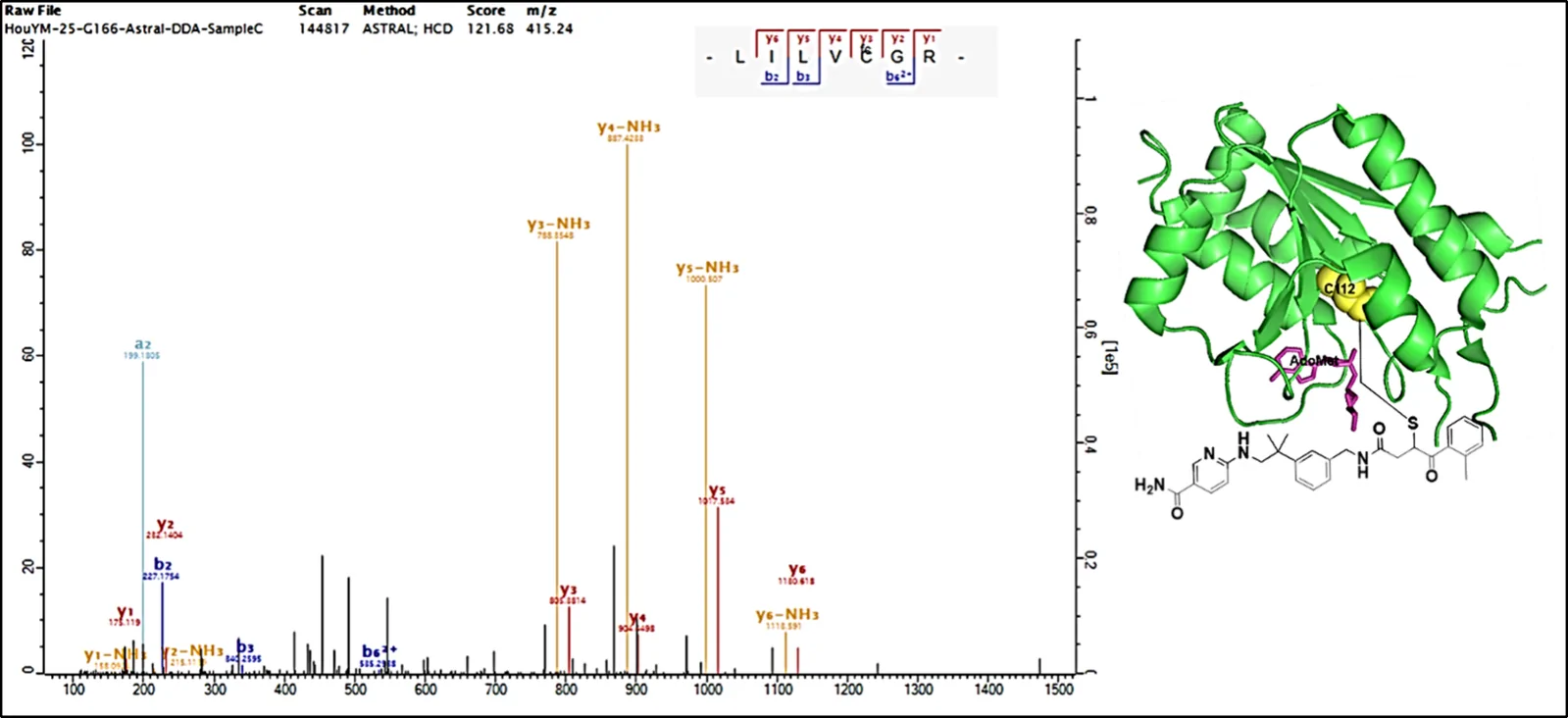
Covalent engagement of TrmD at Cys112 following incubation in *Escherichia coli* cell lysate with **28**. MS/MS spectrum of the peptide LILVCGR following covalent modification by a ∼ 470 Da compound. Fragment ions are annotated as y-type ions (red) and b-type ions (blue); a-type ions (light blue) correspond to b-ions minus CO. Neutral-loss ions (−NH_3_, −17.0265 Da) are indicated in yellow. A continuous y-ion series (y1-y6), including multiple -NH_3_ losses, confirms the peptide sequence and localizes the modification to the cysteine residue. The mass of the covalent modification was determined directly from fragment-ion differences, with the appearance of y3 (C*GR) relative to y2 (GR) corresponding to a +470.23 Da mass shift on cysteine, consistent with covalent attachment of the ∼470 Da compound. Structural model highlighting Cys112 (yellow) positioned adjacent to the AdoMet-binding site (magenta), consistent with scaffold-guided placement of the electrophilic warhead for covalent capture within the TrmD active site.

*In silico* docking studies were subsequently performed to understand this covalent engagement and to define the orientation of the electrophilic substituent relative to Cys112 and the AdoMet binding region (Figure 3). In the predicted binding mode, the nicotinamide-containing scaffold of **28** adopts an extended conformation within the pocket adjacent to the cofactor, forming hydrogen bond interactions with the backbone NH groups of Ile133 and Leu138, as well as a polar contact between the benzyl amide and the backbone carbonyl of Leu87. These anchoring interactions organize the scaffold and orient the cinnamate-derived Michael acceptor toward Cys112, positioning the electrophile in a geometry compatible with covalent bond formation without requiring substantial rearrangement of the bound complex.

**Figure 3:**
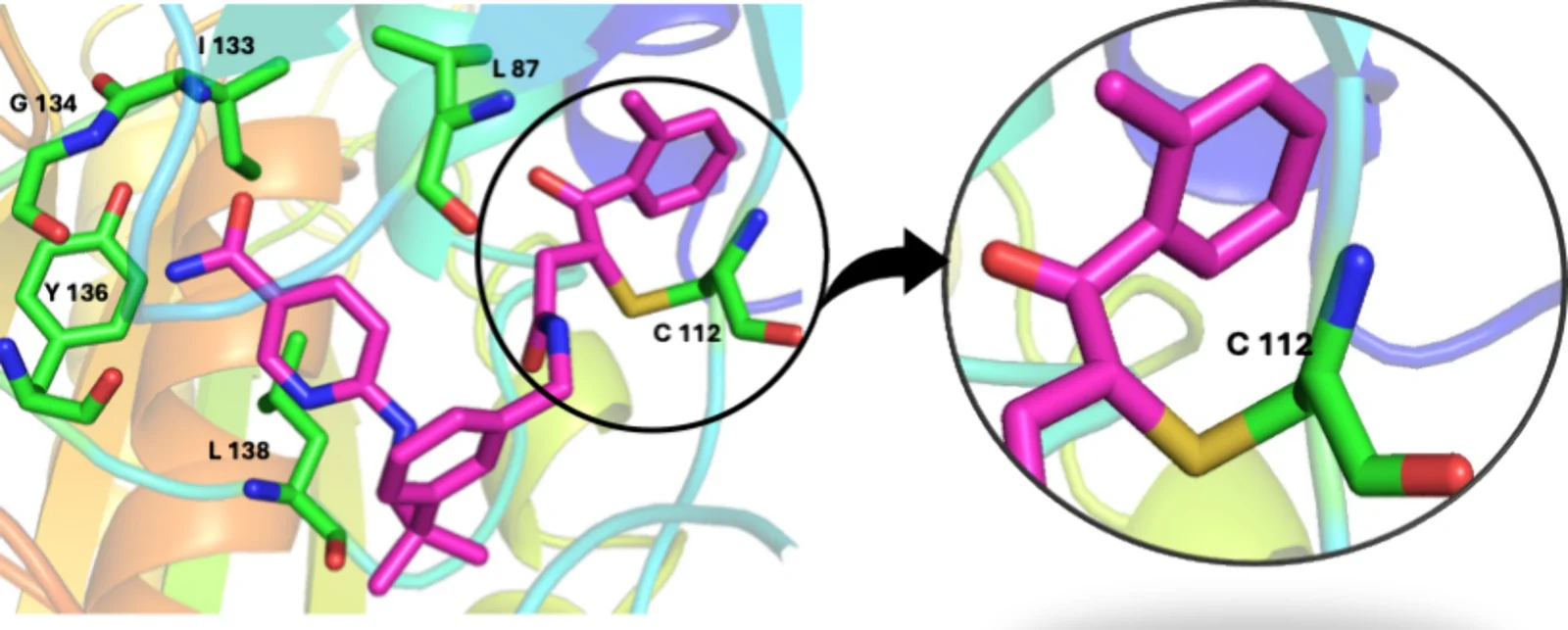
Predicted covalent docking pose of compound **28** (magenta) in the TrmD active site (Docking score = −14.1 kcal/mol), showing placement of the heteroaromatic core in the cofactor-adjacent pocket and orientation of the fumarate-derived Michael acceptor toward Cys112.

Notably, although thiol addition across a polarized C=C bond can in principle occur at either alkene carbon, depending on Cys orientation and adduct stability, conjugate (β) addition is electronically favored for typical α,β-unsaturated carbonyl systems.^41,42^ In the present scaffold, the alkene is positioned between an amide carbonyl and an aryl ketone. Because ketones are stronger π acceptors than amides, whose electrophilicity is attenuated by n→π* donation from nitrogen, the conjugated system is biased toward the aryl ketone. As a result, the β-carbon proximal to the *ortho*-methyl benzoyl group is expected to be more electrophilic than the carbon adjacent to the amide, favoring nucleophilic attack by the Cys112 thiol at this position.^43–45^ Therefore, the docking geometry and intrinsic electronic polarization support Cys112 β addition as the preferred pathway for covalent adduct formation. This model is consistent with the covalent labeling observed by LC-MS/MS following incubation of **28** in *E. coli* cell lysates, supports Cys112 as a chemically addressable hotspot within TrmD, and provides a structural rationale for the improved antibacterial activity of electrophile-containing analogs relative to the parent scaffold.

TrmD enzyme inhibition assay further supported target engagement for our covalent inhibitors (Figure 4). The parent **7** showed strong biochemical potency (IC_50_ = 0.10 ± 0.07 μM), confirming high intrinsic affinity for TrmD; however, this activity did not translate into measurable bacterial growth inhibition. In contrast, covalent inhibitors displayed weaker enzymatic potency (e.g., **12**, IC_50_ = 0.85 ± 0.08 μM; **27**, IC_50_ = 3.00 ± 0.28 μM; **28**, IC_50_ = 4.43 ± 0.44 μM), yet several showed improved antibacterial activities relative to compound **7**. This disconnect highlights that cell-based efficacy is not determined solely by enzyme IC_50_, but also by factors such as target residence time, intracellular exposure, and binding mode.^33^ Consistent with this interpretation, many aryl enone analogs produced larger Δ*T*_m_ values and measurable MICs despite reduced biochemical potency, suggesting that enzyme inhibition IC_50_ alone does not capture the factors controlling whole cell activity. The IC_50_ values reported here come from single-time point measurements and are used only to compare and rank analogs in this study; a follow-up study is underway to measure the full kinetic parameters (*k*_*inact*_ and *K*_*i*_) for the most active analogs.

**Figure 4:**
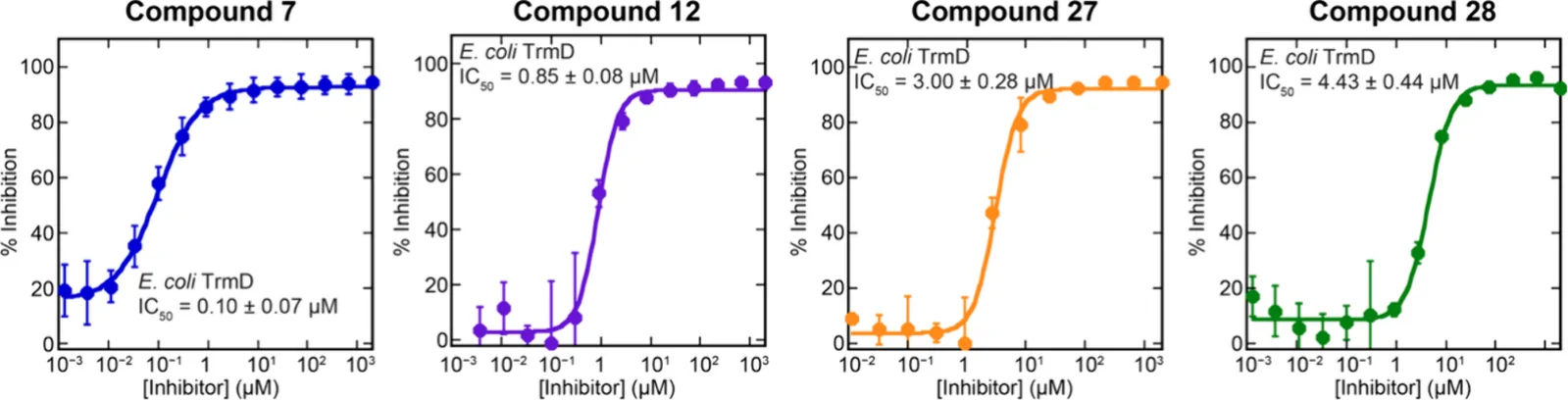
TrmD enzyme inhibition by representative analogs. Dose–response curves for compounds **7**, **12**, **27**, and **28** in the TrmD biochemical assay. Data are plotted as percent inhibition versus inhibitor concentration, and IC_50_ values (mean ± SD) are shown on each panel.

The cellular and biochemical profiling of compounds **11** and **12** supports selective growth inhibition in bacteria expressing TrmD compared to those expressing human Trm5, as shown in Figure 5. In time course colony forming unit (CFU) assays of bacterial cell growth inhibition, both compounds produced rapid and sustained reductions in bacterial viability in the TrmD-expressing strain, whereas growth suppression was slower and less complete in the Trm5-expressing strain, consistent with reduced functional activity when the human ortholog is present. This trend was corroborated by enzyme inhibition data, in which compounds **11** and **12** inhibited *E. coli* TrmD with low micromolar potency (IC_50_ = 1.17 and 0.85 μM, respectively) but showed minimal inhibition of human Trm5 (IC_50_ = 2.17 mM and 1.88 mM, respectively). Collectively, these results indicate that antibacterial activity for covalent inhibitors is driven primarily by selective on-target TrmD inhibition, with substantially weaker inhibition of the human Trm5 enzyme. This selectivity can be explained by the fact that the amino acid residue corresponding to the bacterial reactive Cys112 is absent in human Trm5.

**Figure 5:**
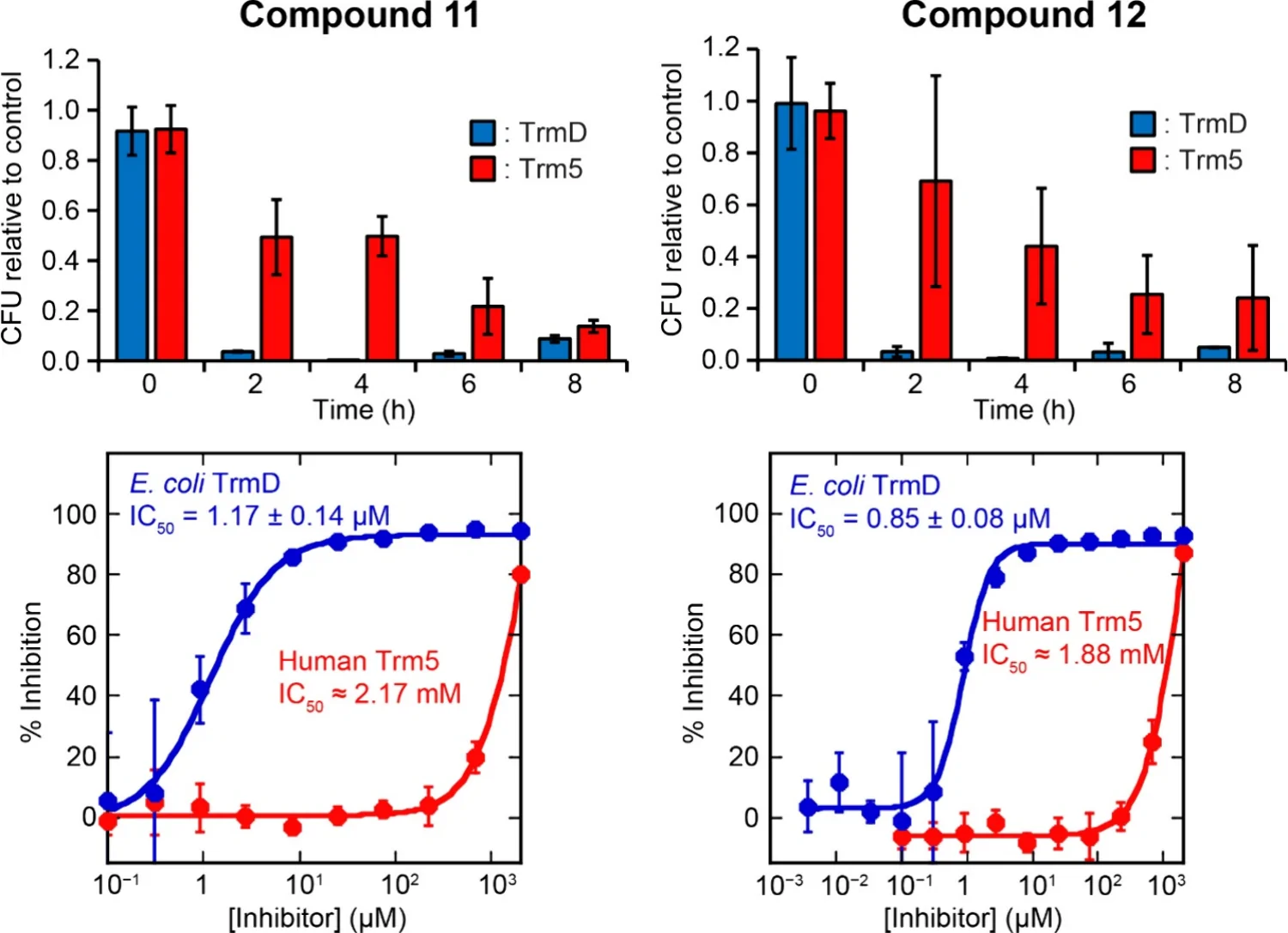
Functional selectivity of compounds **11** and **12** for bacterial TrmD over human Trm5. Top: Time-course bacterial growth inhibition in strains expressing *E. coli* TrmD (blue) or human Trm5 (red) (mean ± SD, *n* = 3). Bottom: Enzyme inhibition dose–response curves showing preferential inhibition of *E. coli* TrmD (blue) relative to human Trm5 (red).

## Conclusion

In summary, we developed a focused series of TrmD inhibitors and established clear structure–activity relationships linking terminal substituent and linker design to target engagement and antibacterial activity. Although the parent scaffold displayed potent biochemical inhibition of TrmD, antibacterial activity remained limited, consistent with previous reports highlighting the difficulty of achieving effective intracellular accumulation, and questioning the druggability of TrmD in Gram-negative bacteria.^19^ Through the incorporation of electrophilic covalent warheads and optimization of aryl enone motifs, we were able to enhance TrmD engagement and obtain measurable antibacterial activity. LC-MS/MS analysis confirmed covalent modification of TrmD at Cys112, a residue located proximally to the AdoMet binding region, supporting a covalent mechanism of inhibition. Importantly, representative compounds demonstrated preferential inhibition of bacterial TrmD over human enzyme Trm5, indicating functional selectivity. Collectively, these findings identify Cys112 as a chemically addressable hotspot and demonstrate that covalent ligand design can overcome some of the limitations previously associated with TrmD inhibition in Gram-negative bacteria. This work therefore provides new support for the druggability of TrmD and establishes a framework for the development of covalent inhibitors of TrmD with an improved cellular activity.

### Experiment Protocol

#### Thermal Shift Assay

Compounds **7**–**43** binding to TrmD were determined by thermal shift assay. Compounds were diluted from 1 mM DMSO stocks in 50 mM HEPES, pH 7.4, 5 mM MgCl_2,_ to a final DMSO assay concentration of 1%. 1.4 μM TrmD protein, 10X Sypro orange, and the compound were combined. Protein was denatured with a stepwise temperature gradient from 20 to 80 °C with 0.5 °C increase every 10 s using a BioRad CFX Opus 384 qPCR machine. Thermal melt temperatures were determined using BioRad CFX Maestro software, and changes were determined relative to DMSO (no compound) controls. Statistical analyses were performed using GraphPad Software.

#### Minimum Inhibitory Concentration (MIC) Assay

We used the hyper-permeable *E. coli* strain GKCW102 for MIC analysis.^47^ This strain was engineered to overexpress a mutant version of the outer membrane siderophore transporter FhuA, inserted into the chromosomal *att*Tn7 locus and activated upon arabinose (Ara) induction. We used P1 transduction to transfer the FhuA locus to the WT strain MG1655 and then removed the kanamycin (Kan) resistance marker of by pCP20. To the resultant transduced strain, termed the pore strain, we prepared an overnight culture in Luria broth (LB) with Ara (0.2%), diluted it by 1:100, and grew cells to optical density at 600 nm (OD_600_) = 0.5 at 37 °C for MIC analysis. In each MIC assay, we used 150 μL of cells at the final concentration of 1 × 10^5^ Colony-forming unit (CFU)/mL to each well of a 96-well plate, containing a serial dilution of the compounds of interest. After incubation at 37 °C for 16 h, we determined MIC by measuring OD_600_ above 0.15 as an indicator of viable cells. Compounds **7** and **28** were tested against two additional Gram-negative pathogens, *Haemophilus influenzae* and *Vibrio cholerae*, which may exhibit greater antibiotic sensitivity, and a Gram-positive pathogen, *Staphylococcus aureus*. One strain each of *H. influenzae* and *V. cholerae*, and six *S. aureus* strains were used, including a methicillin-sensitive strain (MSSA), methicillin-resistant strains (MRSA), and an enterotoxin-producing strain (Table 3). For *H. influenzae*, the assay was performed in the same way as *E. coli*, except that Brain Heart Infusion medium (SIGMA, 53286) supplemented with 10 μg/mL hemin (SIGMA, 51280) and 2 μg/mL β-Nicotinamide adenine dinucleotide (β-NAD, SIGMA, N0632) was used. For *V. cholerae*, the assay was performed in the same way as *E. coli*, except that no Ara was added in the media, and cell cultures were incubated at 30 °C. For *S. aureus*, the assay was performed as for *E. coli*, except that no Ara was added to the media. MIC values were determined in triplicate.

**Table 3:** Gram-Positive and Gram-Negative Bacterial Strains

Species	Strain	Type
*S. aureus*	Newman	MSSA
	M1198	MRSA
	F336222	MRSA
	SU-5	MRSA
	TCH60	MRSA
	FRI361	Enterotoxin C2 producer
*H. influenzae*	Rd KW20	-
*V. cholerae*	TND0905	O1 El Tor Ogawa derivative
*E. coli*	Porin mutant	-

### Growth Inhibition Assay

An overnight culture of the pore strain, together with the WT strain, was inoculated to fresh LB at 1:10 with Ara (0.2%) and grown at 37 °C for 1.5 h. Each culture was then inoculated at 1:50 to LB + Ara with the compound of interest at 100 μM (final concentration) or with dimethyl sulfoxide (DMSO) as a reference. After incubation at 37 °C for 2.5 h, a 3 μL of a 10-fold serial dilution of each culture was spotted onto an LB plate, and after incubation at 37 °C overnight, colonies were counted from the plate with the highest dilution. CFU was calculated by multiplying the number of colonies with the dilution factor and correcting for the plated volume. Each CFU was used to calculate the growth inhibition relative to the DMSO control. To determine how each compound affected cells that do not rely on TrmD for viability, we used a *Trm5* strain where the human *Trm5* gene replaced the chromosomal *TrmD* gene by λRed recombination.^48^ Trm5 is the eukaryotic counterpart for bacterial TrmD, which catalyzes m^1^G37 formation on tRNAs but is fundamentally different in structure.^49,50^ The Trm5 strain was also made into a hyper-permeated strain by P1 transduction as above and was used for the viability assay over the time-course as above, except for 5 h of preincubation with Ara (0.2%). Cells were grown with 100 μM inhibitor and cultures were sampled with compounds **7**–**43** at 0, 2, 4, 6, and 8 h, and the CFU at each time point was calculated relative to DMSO control.

#### Enzyme Inhibition Assay

We prepared recombinant *E. coli* TrmD protein, *E. coli* tRNALeu­(CAG) transcript, and human tRNACys­(GCA) transcript as described.^50–52^ To generate an expression vector for human Trm5 protein, we subcloned the human Trm5 gene into plasmid pEGC at the NotI/XbaI site, and transduced the resultant bacmid to TSA201 cells as described.^53^ The soluble protein in cell lysates was bound to Talon resin and eluted in a buffer containing 20 mM 4-(2-hydroxyethyl)­piperazine-1-ethane-sulfonic acid (HEPES) pH 7.5, 250 mM NaCl, 10 mM β-mercaptoethanol, 0.5 mM phenylmethylsulfonyl fluoride (PMSF), and 40% glycerol. The inhibition assay was done as described previously and IC_50_ was calculated.^54^

#### Production, Purification, and Covalent Labeling of TrmD

The gene encoding TrmD in *E. coli* was modified and amplified via PCR and cloned into a derivative of pMal-C4X (New England Biolabs), where a cut site for the HRV3C protease was inserted between the maltose binding protein (MBP) and TrmD (see for amino acid sequence of the fusion protein). The plasmid was transformed into NiCo21 (DE3) cells (New England Biolabs), which were then grown in 250-ml aliquots of LB broth in 1-L baffled flasks, supplemented with 100 ug/mL ampicillin and 0.1 mM IPTG. The cell cultures were incubated at 30 °C while shaking at 140 R.P.M. for ∼ 15 h. The cells were harvested via centrifugation, and the pellet was frozen at minus 20 °C before further processing. The cells were lysed using B-PER Complete Bacterial Protein Extraction Reagent (ThermoFisher), following the manufacturer’s instructions. The clarified lysate was then applied to 5 mL of amylose resin (New England Biolabs), washed with (25 mM HEPES, pH 7.5, 100 mM NaCl), and eluted with the same buffer, containing 10 mM maltose. HRV3C protease (GenScript) was added to the pooled fractions containing the purified protein to cut the fusion protein, separating MBP from TrmD. Cleavage of the fusion protein was monitored over the course of a few days, until all the fusion protein had been cut, as determined by visual inspection of SDS-PAGE gels. Following cleavage with HRV3C, the reaction mixture was diluted with 5 mM NaPO4 buffer pH 7.8, to bring the NaCl concentration down to 25 mM. To remove the maltose from the solution and from MBP itself, the diluted products were applied to 2 mL of ion-exchange resin (Q-Sepharse FF [Cytiva]) which was washed with column buffer (25 mM NaPO4, pH 7.8) and eluted with a gradient of 0 to 1000 mM NaCl, ramped over 20 column volumes. The products were then repurified on amylose resin, which removed much the of cleaved MBP, with TrmD coming out in the flow-through fraction. Some of the cleaved MBP failed to bind to the amylose resin and came out with the TrmD, but not in quantities that would negatively impact the downstream experiments. The purified protein products were diluted in PBS to a final concentration of ∼ 0.25 mg/mL and the test compounds, **12**, **27**, and **28**, disolved in DMSO, were added to a 5× molar excess. They were then incubated at ∼ 23 °C for 4 h. After the incubation period, aliquots of the labeled proteins were prepared for loading onto an SDS-PAGE gel (NuPAGE Bis-Tris, 4–12 [ThermoFisher]), by heating at 95 °C with 1x NuPAGE LDS Sample Buffer (ThermoFisher) and BME. Six SDS-gel bands were provided to the proteomics and metabolomics facility at Wistar Institute, Philadelphia, PA. The entire stained gel regions were reduced with TCEP, alkylated with iodoacetamide, and digested with trypsin. Tryptic digests were analyzed using a standard 40 min LC gradient on the Thermo Orbitrap Astral mass spectrometer in Data-Dependent Acquisition mode.

Amino acid sequence of the MBP-TrmD Fusion peptide is shown in blue before cleavage
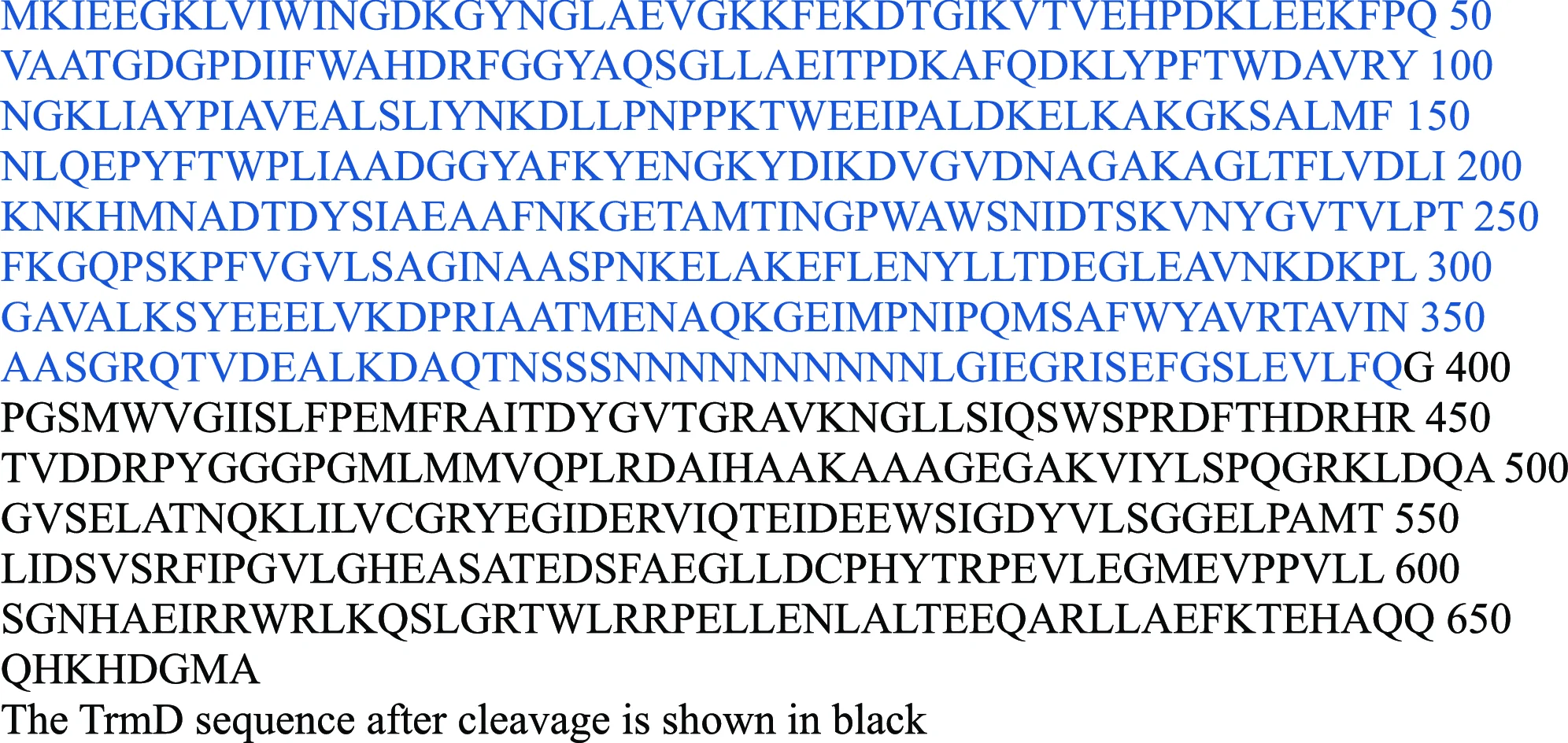


#### RNA Extraction and qPCR for E. coli

RNA was extracted from frozen pellets using Zymo Direct-zol RNA miniprep kit as per manufacturer’s instructions. Samples were treated with an additional dsDNA treatment (Thermo Scientific) before the qPCR. cDNA synthesis and qRT-PCR was performed using iTaq Universal SYBR Green One-Step Kit (Bio-Rad). qRT-PCRs were run on a CFX Opus 384 (Bio-Rad). Relative quantification was determined using the standard ΔΔCt method and normalized to DNA gyrase subunit B. The following primer pairs were used: TrmD forward: 5′- GTCCCTGTGGTGACAGATAAAT-3′, rmD reverse: 5′- GTTAATGATGGTGCAACCCTTG-3′, *gyrB* forward: 5′- AGAGTTATCGGCGTGAATGG-3′, *gyrB* reverse: 5′- CACATGGTATTCGAGGTGGTAG-3′.^55^

#### Covalent Docking

Computational studies were completed in Maestro (Version 14.0.136, MMshare Version 6.6.136, Release 2024-2). The protein structure prediction was carried out using Boltz-2,^56^ in which two full sequence chains and a small molecule, were provided as input for the simulation. The resulting output structure was used for subsequent docking and covalent docking simulations. Before docking any, the ligands and protein were both prepared for simulation. The ligands were prepared using Schrödinger’s LigPrep module, with ionization states adjusted for a pH of 7.4 ± 0.5 while the protein structure was prepared using Schrödinger’s Protein Preparation Workflow, adjusted to a pH of 7.4 ± 0.5. Schrödinger’s Receptor Grid Generation module was then used to define a distinct, localized docking grid for the orthosteric binding site centered around compound **28** on the TrmD dimer structure. Grid dimensions were left as default, 10 Å × 10 Å × 10 Å. (van der Waals radius scaling was kept at default parameters with a scaling factor of 1.0 and a partial charge cutoff of 0.25. No constraints, rotatable groups, or excluded volumes were employed in these grid generations.) Noncovalent ligand (Compound **7**) was XP docked using this standard grid, while ligand featuring a covalent warhead (Compound **28**) was processed through the Glide Covalent Docking module. Cys112 was selected as the reactive residue for covalent docking while the grid was also centered around compound **28** from the TrmD dimer structure featuring the same 10Å x 10Å x 10Å dimensions. Michael addition was selected as the reaction type with the docking mode set to Pose Prediction (Thorough). Protein alignments were performed using the Protein Structure Alignment panel within Maestro.

#### Chemistry

All chemicals and anhydrous solvents were purchased from commercially available sources and used without additional purification. General solvents and reagents were purchased from Fisher Scientific. ^1^H NMR spectra were obtained on a Varian Mercury 400-MHz NMR. Chemical shifts were reported in parts per million (ppm, δ) using various solvents as internal standards (CDCl_3_, δ 7.26 ppm; DMSO-*d*_6_, δ 2.49 ppm). ^1^H NMR splitting patterns were designated as singlet (s), doublet (d), triplet (t), or quartet (q). Splitting patterns that could not be interpreted or easily visualized were recorded as multiplet (m) or broad (br). Coupling constants were reported in hertz (Hz). Liquid chromatography–mass spectrometry was performed on a Waters Alliance 2695 HPLC/MS (Waters Symmetry C18, 4.6 × 75 mm, 3.5 mm) with a 2996 diode array detector from 210 to 400 nm equipped with a Micromass ZQ mass spectrometer and ESI detector. High-resolution mass was determined by the Mass Spectrometry and Proteomics Facility at the University of Notre Dame, Department of Chemistry and Biochemistry, Notre Dame, IN. Silica gel column purifications were performed on prepacked silica gel cartridges, using gradients of ethyl acetate/hexanes or methanol/methylene chloride. Reverse phase purification was done with Gilson 215 liquid handler, Gilson 306 50SC pumps and Gilson UV/vis-155, Sunfire C18 OBD 10 mM 30 × 150 mm and mobile phase acetonitrile, water with 0.1% TFA. Purity of the final compounds was determined by LC-MS using the area percentage method on the UV trace recorded at a wavelength of 254 nm and found to be >95%. Detailed chemistry experiment protocol provided in (SI).

## Supplementary Material







## Notes

Proteomics data set identifying TrmD peptides (XLSX)
LC-MS/MS analysis of covalent binding and peptide-level modification (XLSX)
Supplementary figures (Figures S1–S2), supplementary tables (Tables S1–S2), chemical synthesis protocols, reaction schemes, compound characterization data including ^1^H NMR spectra, and references (PDF)

## Notes

No unexpected or unusually high safety hazards were encountered.

## Abbreviations

AdoMet: S-adenosyl methionine
AMR: antimicrobial resistance
CFU: colony-forming units
Cys: cysteine
DCM: dichloromethane
DIPEA: N,N-diisopropylethylamine
DMF: N, N-dimethylformamide
DMSO: dimethyl sulfoxide
DSF: differential scanning fluorimetry
Δ*T*_m_: change in melting temperature
*E. coli*: *Escherichia coli*
HATU: 1-[bis­(dimethylamino)­methylene]-1H-1,2,3-triazolo­[4,5-*b*]­pyridinium 3-oxid hexafluorophosphate
IC_50_: half-maximal inhibitory concentration
LC-MS/MS: liquid chromatography-tandem mass spectrometry
m^1^G37: *N* ^1^-methylguanosine at position 37
MIC: minimum inhibitory concentration
MS: mass spectrometry
NBS: N-bromosuccinimide
NI: no inhibition
NT: not tested
RaNi: Raney nickel
RT: room temperature
SAR: structure–activity relationship
SPOUT: SpoU-TrmD family
TEA: triethylamine
THF: tetrahydrofuran
tRNA: Transfer RNA
TrmD: tRNA (m^1^G37) methyltransferase
Trm5: eukaryotic tRNA (m^1^G37) methyltransferase

## References

[1] Hutchings M. I., Truman A. W., Wilkinson B. (2019). Antibiotics: Past, Present and Future. Curr. Opin. Microbiol..

[2] Tacconelli E., Carrara E., Savoldi A. (2018). Discovery,
Research,
and Development of New Antibiotics: The WHO Priority List of Antibiotic-Resistant
Bacteria and Tuberculosis. Lancet Infect. Dis..

[3] Laxminarayan R., Sridhar D., Blaser M., Wang M., Woolhouse M. (2016). Achieving
Global Targets for Antimicrobial Resistance. Science.

[4] Lewis K. (2020). The Science
of Antibiotic Discovery. Cell.

[5] Nikaido H. (2003). Molecular
Basis of Bacterial Outer Membrane Permeability Revisited. Microbiol. Mol. Biol. Rev..

[6] Delcour A. H. (2009). Outer Membrane
Permeability and Antibiotic Resistance. Biochim.
Biophys. Acta.

[7] Richter M. F., Drown B. S., Riley A. P. (2017). Predictive
Compound
Accumulation Rules Yield a Broad-Spectrum Antibiotic. Nature.

[8] Masi M., Réfrégiers M., Pos K. M., Pagès J.-M. (2017). Mechanisms
of Envelope Permeability and Efflux in Gram-Negative Bacteria. Nat. Rev. Microbiol..

[9] Wilson D. N. (2014). Ribosome-Targeting
Antibiotics and Mechanisms of Bacterial Resistance. Nat. Rev. Microbiol..

[10] Arenz S., Wilson D. N. (2016). Bacterial Protein Synthesis as a Target for Antibiotic
Inhibition. Cold Spring Harb. Perspect. Med..

[11] Christian T., Hou Y.-M. (2007). Distinct Determinants
of tRNA Recognition by the TrmD
and Trm5Methyl Transferases. J. Mol. Biol..

[12] Björk G. R., Hagervall T. G. (2014). Transfer
RNA Modification: Presence, Synthesis, and
Function. EcoSal Plus.

[13] Gamper H. B., Masuda I., Frenkel-Morgenstern M., Hou Y.-M. (2015). Maintenance of Protein
Synthesis Reading Frame by EF-P and m^1^G37-tRNA. Nat. Commun..

[14] Masuda I., Hwang J.-Y., Christian T., Maharjan S., Mohammad F., Gamper H., Buskirk A. R., Hou Y.-M. (2021). Loss of N1-Methylation
of G37 in tRNA Induces Ribosome Stalling and Reprograms Gene Expression. eLife.

[15] Masuda I., Yamaki Y., Detroja R., Tagore S., Moore H., Maharjan S., Nakano Y., Christian T., Matsubara R., Lowe T. M., Frenkel-Morgenstern M., Hou Y.-M. (2022). tRNA Methylation Resolves Codon Usage Bias at the Limit
of Cell Viability. Cell Reports.

[16] Reddy T. B. K., Riley R., Wymore F., Montgomery P., DeCaprio D., Engels R., Gellesch M., Hubble J., Jen D., Jin H., Koehrsen M., Larson L., Mao M., Nitzberg M., Sisk P., Stolte C., Weiner B., White J., Zachariah Z. K., Sherlock G., Galagan J. E. (2007). Comparative
Genomic Assessment of Novel Broad-Spectrum Targets for Antibacterial
Drugs. Antimicrob. Agents Chemother..

[17] Zhong W., Yang H. (2019). Discovery
of Novel tRNA-(N1G37) Methyltransferase (TrmD)
Inhibitors as Antibacterial Agents. ACS Infect.
Dis..

[18] Zhong W., Wu J. (2019). Thienopyrimidinone Derivatives That Inhibit Bacterial
tRNA (N1G37) Methyltransferase TrmD. J. Med.
Chem..

[19] Wilkinson A. J., Ooi N., Finlayson J., Lee V. E., Lyth D., Maskew K. S., Newman R., Orr D., Ansell K., Birchall K., Canning P., Coombs P., Fusani L., McIver E., Pisco J., Ireland P. M., Jenkins C., Norville I. H., Southern S. J., Cowan R., Hall G., Kettleborough C., Savage V. J., Cooper I. R. (2023). Evaluating the Druggability of TrmD,
a Potential Antibacterial Target, through Design and Microbiological
Profiling of a Series of Potent TrmD Inhibitors. Bioorg. Med. Chem. Lett..

[20] Hou Y.-M., Masuda I., Foster L. J. (2020). tRNA Methylation: An Unexpected Link
to Bacterial Resistance and Persistence to Antibiotics and Beyond. Wiley Interdiscip. Rev. RNA.

[21] Masuda I., Matsubara R., Christian T., Rojas E. R., Yadavalli S. S., Zhang L., Goulian M., Foster L. J., Huang K. C., Hou Y.-M. (2019). tRNA Methylation Is a Global Determinant of Bacterial
Multi-drug Resistance. Cell Syst..

[22] Goto-Ito S., Ito T., Yokoyama S. (2017). Trm5 and TrmD:
Two Enzymes from Distinct Origins Catalyze
the Identical tRNA Modification, m1G37. Biomolecules.

[23] Hou Y.-M., Masuda I., Gamper H. (2019). Codon-Specific
Translation by m^1^G37 Methylation of tRNA. Front. Genet..

[24] Hou Y.-M., Matsubara R., Takase R., Masuda I., Sulkowska J. I. (2017). TrmD: A
Methyl Transferase for tRNA Methylation With m^1^G37. Enzymes.

[25] Sakaguchi R., Lahoud G., Christian T., Gamper H., Hou Y.-M. (2014). A Divalent
Metal Ion-Dependent N(1)-Methyl Transfer to G37-tRNA. Chem. Biol..

[26] Sakaguchi R., Giessing A., Dai Q., Lahoud G., Liutkeviciute Z., Klimasauskas S., Piccirilli J., Kirpekar F., Hou Y.-M. (2012). Recognition
of Guanosine by Dissimilar tRNA Methyltransferases. RNA.

[27] Christian T., Lahoud G., Liu C., Hou Y.-M. (2010). Control of Catalytic
Cycle by a Pair of Analogous tRNA Modification Enzymes. J. Mol. Biol..

[28] Christian T., Evilia C., Hou Y.-M. (2006). Catalysis
by the Second Class of
tRNA­(m^1^G37) Methyl Transferase Requires a Conserved Proline. Biochemistry.

[29] Ito T., Masuda I., Yoshida K. (2015). Structural Basis for
Methyl-Donor-Dependent and Independent Binding of S-Adenosyl-L-methionine
Analogues to TrmD. Proc. Natl. Acad. Sci. U.S.A..

[30] Thomas S. E., Mendes V., Kim S. Y. (2020). Fragment-Based Discovery
of a New Class of Inhibitors Targeting TrmD, an Essential Bacterial
tRNA Methyltransferase. Nucleic Acids Res..

[31] Hill P. J., Abibi A., Albert R., Andrews B., Gagnon M. M., Gao N., Grebe T., Hajec L., Huang J., Jones M., Kohli R. M., Linsell M. S., MacDougall J., McDevitt D., Minassian B. F., Projan S. J., Rittenhouse S., Sheridan R. P., Uria-Nickelsen M., Wang J., Wu T., Ye X., Zhang Y., Zhu T. (2013). Structure-Guided Discovery
of Selective Inhibitors of the Bacterial tRNA (m^1^G37) Methyltransferase
TrmD. J. Med. Chem..

[32] Whitehouse A. J., Thomas S. E., Brown K. P., Fanourakis A., Chan D. S.-H., Libardo M. D. J., Mendes V., Boshoff H. I. M., Floto R. A., Abell C., Blundell T. L., Coyne A. G. (2019). Structure-Guided
Development of Inhibitors of TrmD from *Mycobacterium abscessus*. J. Med. Chem..

[33] Singh J., Petter R. C., Baillie T. A., Whitty A. (2011). The Resurgence of Covalent
Drugs. Nat. Rev. Drug Discovery.

[34] Baillie T. A. (2016). Targeted
Covalent Inhibitors for Drug Design. Angew.
Chem., Int. Ed..

[35] De
Cesco S., Kurian J., Dufresne C., Mittermaier A. K., Moitessier N. (2017). Covalent Inhibitors: A Rational Approach to Drug Discovery. Eur. J. Med. Chem..

[36] Gehringer M., Laufer S. A. (2019). Emerging and Re-Emerging Warheads
for Targeted Covalent
Inhibitors: Applications in Medicinal Chemistry and Chemical Biology. J. Med. Chem..

[37] Lagoutte R., Patouret R., Winssinger N. (2017). Covalent Inhibitors:
An Opportunity
for Rational Target Selectivity. Curr. Opin.
Chem. Biol..

[38] Ward C. C., Kleinman J. I., Brittain S. M., Lee P. S., Chung C. Y. S., Kim K., Petri Y., Thomas J. R., Tallarico J. A., McKenna J. M., Schirle M., Nomura D. K. (2019). Covalent Ligand
Screening Uncovers a RNF4 E3 Ligase Recruiter for Targeted Protein
Degradation Applications. ACS Chem. Biol..

[39] Elharar Y., Roth Z., Hecht N. (2014). Posttranslational regulation
of proteasomal degradation by the Pup-proteasome system. Proc. Natl. Acad. Sci. U.S.A..

[40] Bonjorno A. F., Pavan A. R., Fernandes G. F. S., Scarim C. B., Castagnolo D., Dos Santos J. L. (2024). BacPROTACs
Targeting Clp Protease: A Promising Strategy
for Anti-Mycobacterial Drug Discovery. Front.
Chem..

[41] Krishnan S., Miller R. M., Tian B., Mullins R. D., Jacobson M. P., Taunton J. (2014). Design of Reversible,
Cysteine-Targeted Michael Acceptors
Guided by Kinetic and Computational Analysis. J. Am. Chem. Soc..

[42] Jackson P. A., Widen J. C., Harki D. A., Brummond K. M. (2017). A Chemical Perspective
on the Reactivity of α,β-Unsaturated Carbonyls toward
Thiols in Biological Systems. J. Med. Chem..

[43] Allgäuer D. S., Steigelmann M., Mayr H. (2017). Quantification and Theoretical Analysis
of the Electrophilicities of Michael Acceptors. J. Am. Chem. Soc..

[44] Choudhary A., Fry C. G., Kamer K. J., Raines R. T. (2013). An n→π*
Interaction Reduces the Electrophilicity of the Acceptor Carbonyl
Group. Chem. Commun..

[45] Newberry R. W., Raines R. T. (2017). The n→π*
Interaction. Acc. Chem. Res..

[47] Krishnamoorthy G., Wolloscheck D., Weeks J. W., Croft C., Rybenkov V. V., Zgurskaya H. I. (2016). Breaking
the Permeability Barrier of *Escherichia
coli* by Controlled Hyperporination of the Outer Membrane. Antimicrob. Agents Chemother..

[48] Datsenko K. A., Wanner B. L. (2000). One-step inactivation
of chromosomal genes in Escherichia
coli K-12 using PCR products. Proc. Natl. Acad.
Sci. U. S. A..

[49] Christian T., Evilia C., Williams S., Hou Y. M. (2004). Distinct Origins
of tRNA­(m1G37) Methyltransferase. J. Mol. Biol..

[50] Christian T., Sakaguchi R., Perlinska A. P., Lahoud G., Ito T., Taylor E. A., Yokoyama S., Sulkowska J. I., Hou Y. M. (2016). Methyl Transfer by Substrate Signaling from a Knotted
Protein Fold. Nat. Struct Mol. Biol..

[51] Christian T., Gamper H., Hou Y. M. (2013). Conservation of structure and mechanism
by Trm5 enzymes. RNA.

[52] Masuda I., Sakaguchi R., Liu C., Gamper H., Hou Y.-M. (2013). The temperature
sensitivity of a mutation in the essential tRNA modification enzyme
tRNA methyltransferase D (TrmD). J. Biol. Chem..

[53] Li Y., Ruan Z., Lee J., Orozco I. J., Zhou E., Du J., Lü W. (2025). Structural
basis of PANX1 permeation and positive modulation
by mefloquine. Nat. Commun..

[54] Bader C. D., Masuda I., Nichols A., Kalkreuter E., Yang D., Christian T., Nakano Y., Hou Y. M., Shen B. (2025). Discovery of the 5-Chlorotryptophan-Containing Antibiotics through
Metabologenomics-assisted High-throughput Screening. JACS Au.

[55] Clifton B. E, Fariz M. A, Uechi G.-I., Laurino P. (2021). Evolutionary repair
reveals an unexpected role of the tRNA modification m^1^G37
in aminoacylation. Nucleic Acids Res..

[56] Passaro, S. ; Corso, G. ; Wohlwend, J. ; Reveiz, M. ; Thaler, S. ; Somnath, V. R. ; Getz, N. ; Portnoi, T. ; Roy, J. ; Stark, H. ; Kwabi-Addo, D. ; Beaini, D. ; Jaakkola, T. ; Barzilay, R. Boltz-2: Towards Accurate and Efficient Binding Affinity Prediction. bioRxiv. 2025, 10.1101/2025.06.14.659707.

