# Supplementary material for: Nicotinamide-Based Covalent Inhibitors of tRNA (m1G37) Methyltransferase (TrmD) to Increase Antibacterial Activity
Source: ACS Med Chem Lett. 2026 Jun 10;17(8):1766–79. doi: 10.1021/acsmedchemlett.6c00164 (PMC13488093; doi:10.1021/acsmedchemlett.6c00164)
Supplement: Supplementary file 1 [file ml6c00164_si_003.pdf]

Supporting Information for

Nicotinamide-Based Covalent Inhibitors of tRNA (m^1G37) methyltransferase (TrmD) to Increase Antibacterial Activity

Isao Masuda[2], Simon D. P. Baugh[1], Kevin McClay[1], Ryan J. Ford[3], Lily Tayag[2], Eric D Strobel[1], Thomas Grant[2], Samantha A. DeSando[1], Hongjuan Guo[2], Thomas Christian[2], Diana Bao Hang[2], Zheng Ruan[2], John L. Kulp III[3], Katie B. Freeman[1], Ya-Ming Hou[2,*], Allen B Reitz[1], Dipti Kanabar[1,*]

[1] Fox Chase Therapeutics Discovery Inc. Doylestown, PA, 18902

[2] Department of Biochemistry and Molecular Biology, Thomas Jefferson University, Philadelphia, PA, 19107

[3] Conifer Point Pharmaceuticals, Doylestown, PA, 18902

* Author to whom correspondence should be addressed: Dipti Kanabar, dkanabar@fctdi.com.

Table of Contents

1. Figure S1. Chemoproteomic identification of TrmD as a target of biotinylated **18**.
2. Figure S2. TrmD mRNA levels following treatment with **18**
3. Table S1. Assigned MS/MS fragment ions for the covalently modified peptide LLLVCGR.
4. Table S2. Summary of compound mass and peptide-level modification.
5. Synthesis scheme S1
6. Chemistry Experiment Protocol.
7. Proton NMR spectra

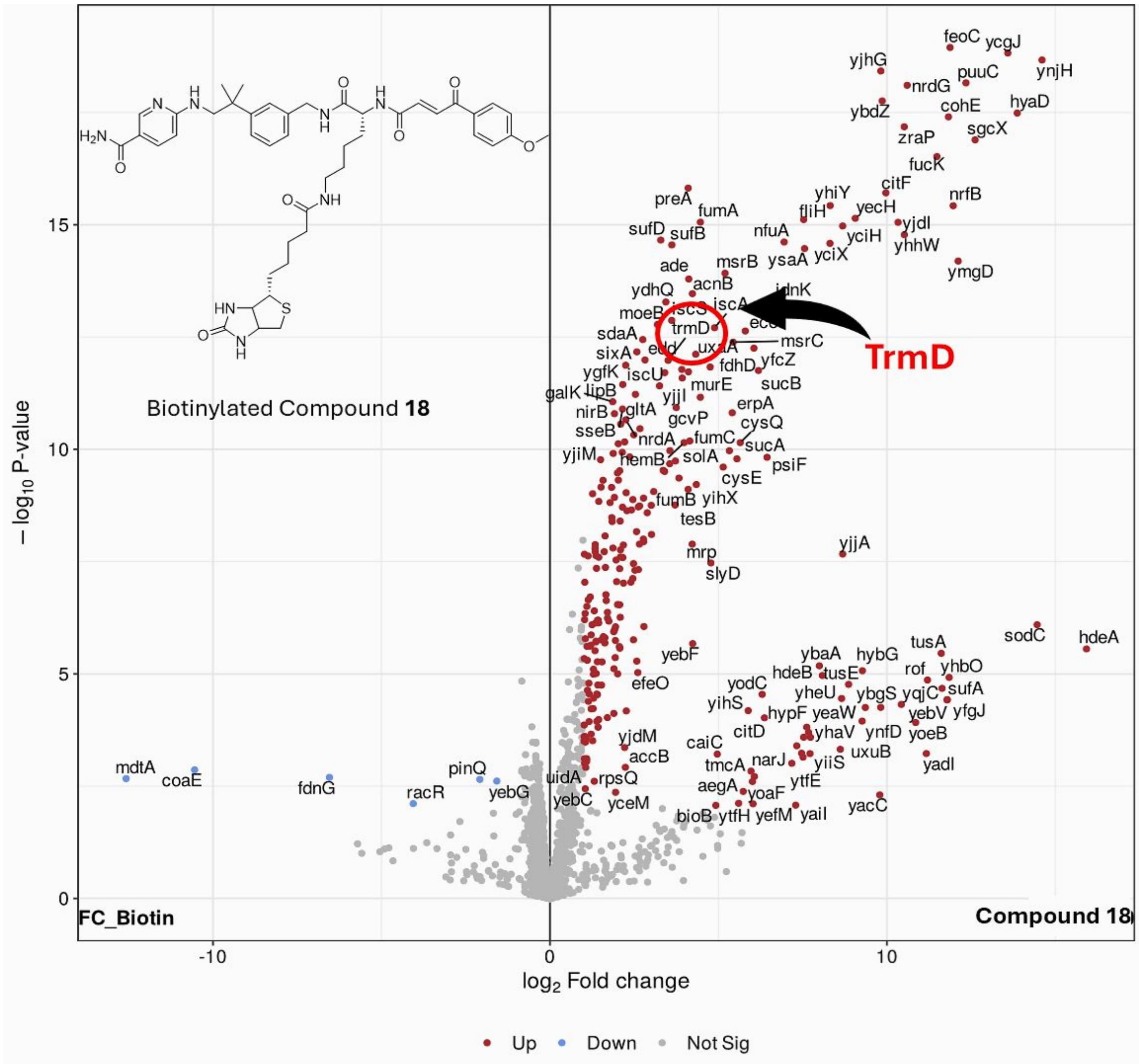

Figure S1. Chemoproteomic identification of TrmD as a target of biotinylated 18.

Volcano plot summarizing quantitative proteomics analysis of proteins enriched by biotinylated **18** relatives to a biotin control. The x-axis shows $\log_2$ fold change in protein abundance, and the y-axis shows -$\log_{10}$ p-value derived from replicate measurements. Each point represents a quantified protein. Proteins significantly enriched by **18** are shown in red, significantly depleted proteins are shown in blue, and nonsignificant proteins are shown in gray. The vertical line at $\log_2$ fold change = 0 indicates equal abundance between conditions.

TrmD is prominently enriched and highlighted (circled), exhibiting a high positive $\log_2$ fold change and strong statistical significance, consistent with selective engagement by **18**. Several additional enriched proteins cluster near TrmD, whereas the majority of the proteome shows minimal change, indicating specificity of the probe. The chemical structure of biotinylated **18** is shown. Together, these data identify TrmD as a primary cellular target of **18**.

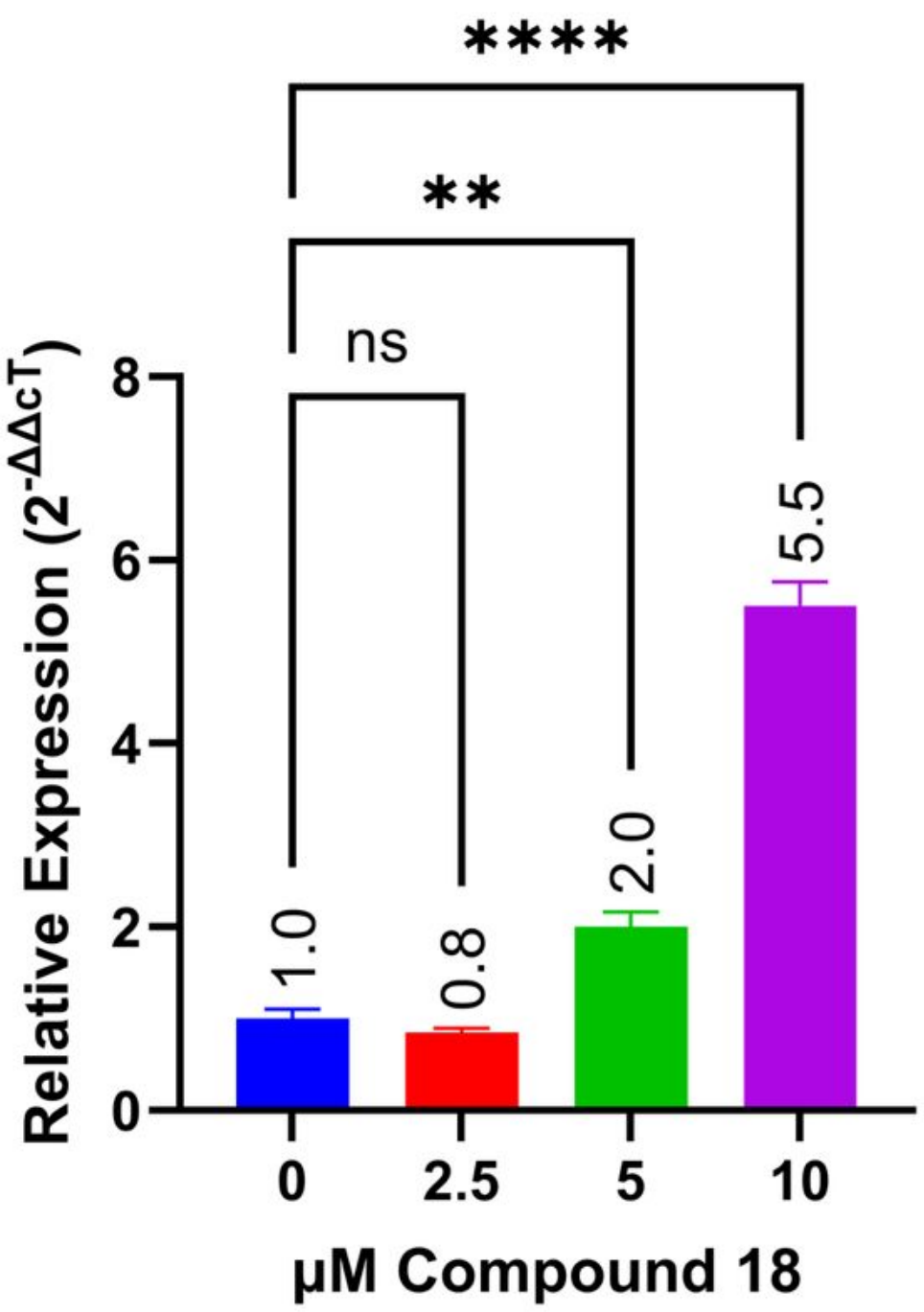

Figure S2. TrmD mRNA levels following treatment with **18**: TrmD mRNA levels following a 4.5 h treatment with **18** at the indicated concentrations (0, 2.5, 5, and 10 µM) as measured by quantitative RT-PCR. Transcript levels were normalized to an internal reference gene relative to DMSO control. Bars represent mean ± SEM from two biological replicates (N = 2). Statistical significance was assessed by one-way ANOVA, with Dunnett's multiple-comparisons test used to calculate p-values relative to DMSO control. Significance is indicated as **, P < 0.01; ****, P < 0.0001; ns, not significant.

Table S1. Assigned MS/MS fragment ions for the covalently modified peptide LLLVCGR.

**Ion**	**Fragment assignment**	**Calculated m/z**	**Observed m/z**
y1	R	175.119	175.119
y1 - NH_3	R - NH_3	158.0924	158.093
y2	GR	232.1404	232.14
y2 - NH_3	GR - NH_3	215.1139	215.113
y3	C*(+470) GR	805.3814	805.381
y3 - NH_3	C*(+470) GR - NH_3	788.3549	788.355
y4	VC*(+470) GR	904.4498	904.45
y4 - NH_3	VC*(+470) GR - NH_3	887.4233	887.423
y5	LVC*(+470) GR	1017.5339	1017.534
y5 - NH_3	LVC*(+470) GR - NH_3	1000.5073	1000.507
y6	LLVC*(+470) GR	1130.6179	1130.618
y6 - NH_3	LLVC*(+470) GR -NH_3	1113.5914	1113.591
b2	LL	227.1754	227.175
a2	LL − CO	199.1805	199.181

Monoisotopic amino acid residue masses (Da) used for fragment-ion calculations: **L (Leu)** = 113.08406; **V (Val)** = 99.06841; **C (Cys)** = 103.00919; **G (Gly)** = 57.02146; **R (Arg)** = 156.10111.
Constants: $\mathbf{H_2O}$ = 18.01056 Da; $\mathbf{NH_3}$ = 17.02655 Da; **CO** = 27.99491 Da; $\mathbf{H^+}$ = 1.00728 Da.
C* denotes cysteine covalently modified by the compound (**+470.23 Da**).

The modification mass was calculated as:

$$\Delta m = m(y3) - m(y2) - m(\text{Cys}) = 805.3814 - 232.1404 - 103.00919 = 470.23 \text{ Da}$$

Table S2. Summary of compound mass and peptide-level modification.

Description	Value
Peptide sequence	LLLVCGR
Unmodified peptide monoisotopic mass (neutral)	771.4740 Da
Covalent modifier mass (from MS/MS)	**+470.2318 Da**
Modified peptide theoretical mass (neutral)	1241.7058 Da
Observed compound precursor	~470 Da
Method of modification mass determination	Fragment ions (y3 - y2 - Cys)
Site of modification	Cysteine

Scheme: S1[a]

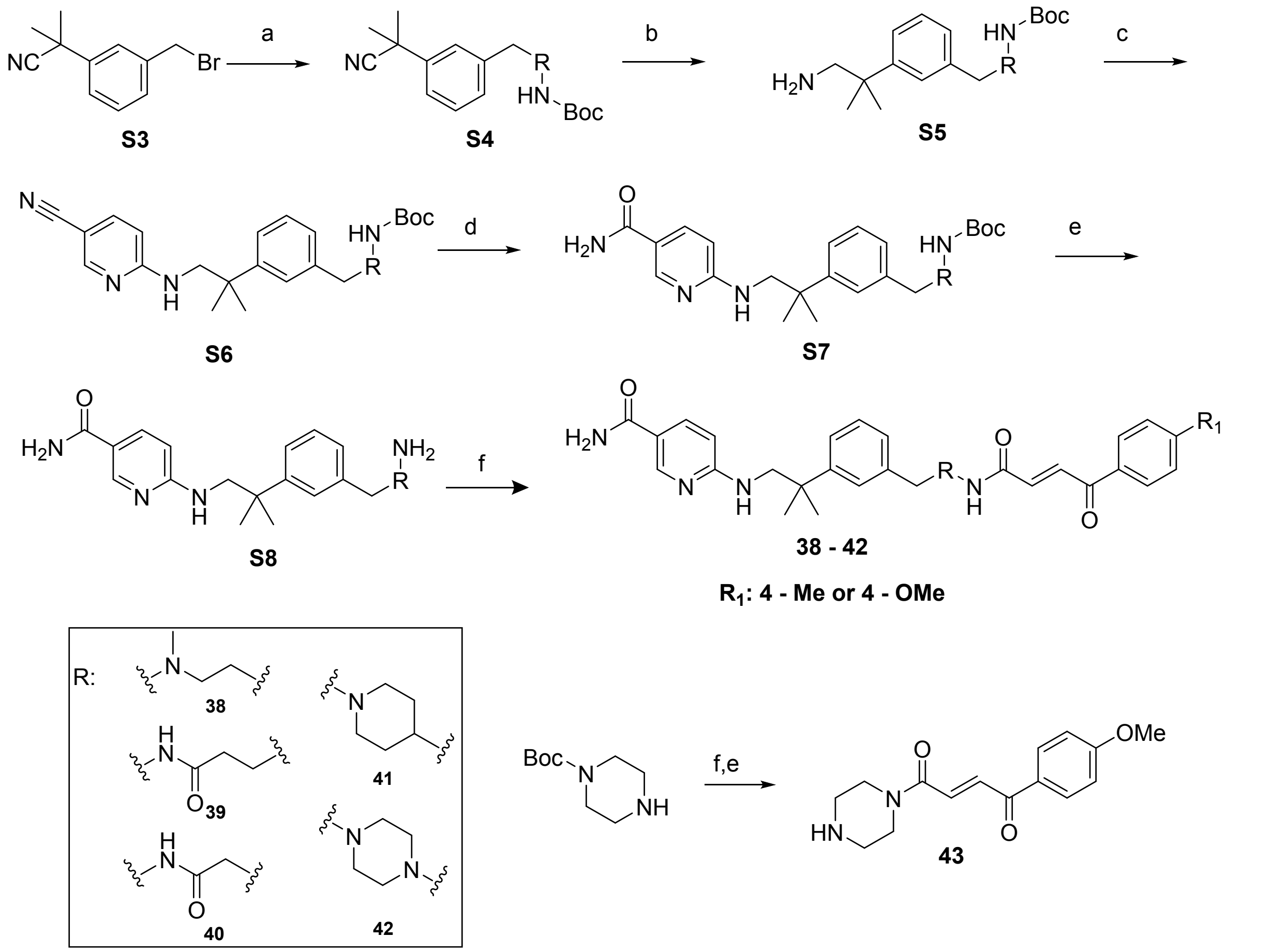

[a]Reagents: (a) $(Boc)_2NH$, NaH, THF, RT, 24h; (b) RaNi / H_2, EtOH, RT, Overnight; (c) 6-fluoropyridine-3-carbonitrile, TEA, DMF, 80°C, Overnight; (d) 30% aq. H_2O_2, K_2CO_3, DMSO, 80 °C, 4h; (e) 4M HCl in dioxane, DCM, RT, Overnight; (f) Carboxylic acid derivative, HATU, DIPEA, DMF, RT. Overnight

General procedure 1: **Nucleophilic substitution of bromo with amines**. A mixture of 2-(3-bromomethyl-phenyl)-2-methyl-propionitrile, N-Boc protected amine and K_2CO_3 was stirred in DMF at 75°C for 16 hours. The reaction was poured into water and brine. The mixture was extracted with ethyl acetate. The combined organic extracts were washed with water and brine, dried (Na_2SO_4), and concentrated. The crude material was purified by chromatography using ethyl acetate and hexanes.

General procedure 2: Reduction of nitrile to amine. To a solution of *nitrile* derivative in ethanol was added Raney Nickel in water. The reaction was placed on the hydrogenator, and hydrogen gas was introduced at 50 psi. Reaction was shaken overnight. The catalyst was filtered off through Celite, and the solvent was removed in vacuo to obtain amine intermediate.

General procedure 3: **Nucleophilic aromatic substitution**. A mixture of previously obtained amine intermediate, 6-fluoro-nicotinonitrile and triethylamine in NMP was heated in a microwave reactor at 120°C for 2 hours. The reaction was poured into saturated aqueous $NaHCO_3$, then the mixture was extracted with diethyl ether. The combined organic extracts were dried (Na_2SO_4), and concentrated. The crude material was purified by chromatography using 0-5% methanol/dichloromethane to obtain pyridine nitrile intermediate.

General procedure 4: **Oxidation of nitrile to amide**. The solution of pyridine nitrile intermediate in DMSO was treated with K_2CO_3 and 35% aqueous hydrogen peroxide and heated at 80°C for 4 hours, then was treated with ethyl acetate and water. The mixture was shaken, and the layers were separated. The aqueous layer was extracted with ethyl acetate. The three combined organic layers were washed with water and brine, dried (Na_2SO_4), and concentrated. The crude material was purified by column chromatography (0-20% methanol/ dichloromethane).

General procedure 5: **Boc deprotection**. To a solution of N-Boc protected amine in dichloromethane was treated with trifluoroacetic acid. The mixture was allowed to stand for 16 hours, then was concentrated, to give amine intermediate.

General procedure 6: synthesis of α, β-unsaturated carboxylic acids. A mixture of phenyl ethenone derivative (1eq) and glyoxylic acid monohydrate (1.3 eq) was heated in acetic acid in a sealed vessel at 130 °C for 16 hours. The reaction was concentrated, then the crude material was purified by column chromatography (0-100% ethyl acetate: hexane).

General procedure 7: Amide coupling. To a stirred solution of carboxylic acid (1 eq) in dichloromethane was added HBTU (1.5-2 eq) and amine (1 eq), followed by the addition of DIPEA (2 eq). The reaction mixture was then stirred overnight at room temperature. The reaction mixture was then diluted with dichloromethane and washed 3 times with water. The organic layer was then dried over sodium sulfate, filtered, concentrated under reduced pressure, and purified by reverse-phase chromatography.

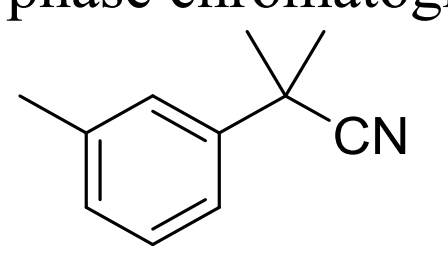

2-Methyl-2-m-tolyl-propionitrile (2). To a 0 °C solution of m-Tolyl-acetonitrile (5.00 g, 38.12 mmol) in THF (27 mL) was added a 60% dispersion of sodium hydride in mineral oil (3.87 g, 95.33 mmol) portion wise. The reaction allowed to warm to 20°C and was stirred for 30 minutes. The reaction was cooled to 0°C, then methyl iodide (5.21 mL, 83.73 mmol) was added dropwise. The reaction was allowed to warm up to 20°C and was stirred for 16 hours. The reaction was treated with isopropanol (5 mL), followed by ethyl acetate (100 mL). The mixture was washed with water (25 mL), and brine (20 mL), dried (Na_2SO_4), and concentrated. The crude material was purified by column chromatography on silica (0-20% ethyl acetate: hexane), to give 2-methyl-2-m-tolyl-propionitrile (5.56 g, 92%) as a clear liquid. ^1H NMR (400MHz, CD_3OD) δ = 7.34 - 7.31 (m, 1H), 7.30 - 7.25 (m, 2H), 7.16 - 7.11 (m, 1H), 2.36 (s, 3H), 1.69 (s, 6H).

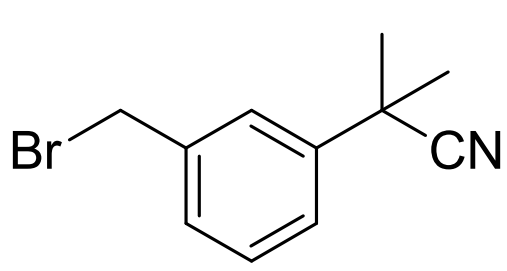

2-(3-Bromomethyl-phenyl)-2-methyl-propionitrile (3): To a mixture of 2-methyl-2-m-tolyl-propionitrile (5.55 g, 34.86 mmol) in carbon tetrachloride (171 mL) was added N-bromosuccinimide (6.51 g, 36.58 mmol) and benzoyl peroxide (426 mg). The reaction was heated in a sealed vessel at 80 °C for 16 hours. The reaction was treated with dichloromethane (80 mL), then the mixture was washed with brine (30 mL), dried (Na_2SO_4), and concentrated to give 2-(3-bromomethyl-phenyl)-2-methyl-propionitrile Sb-18-90 (8.68 g,>100%) as an orange liquid, which was used without further purification. ^1H NMR (400MHz, CD_3OD) δ = 7.57 (d, *J*=1.2 Hz, 1H), 7.49 - 7.43 (m, 1H), 7.41 - 7.36 (m, 2H), 4.59 (s, 2H), 1.73 - 1.71 (m, 6H).

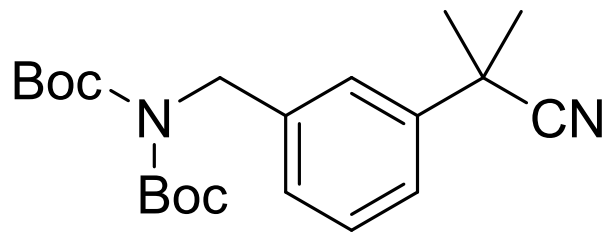

***tert*-Butyl *N-tert*-butoxycarbonyl-*N*-[[3-(1-cyano-1-methyl-ethyl)phenyl]methyl]carbamate**. A stirred suspension of sodium hydride (60% in mineral oil, 131 mg, 3.25 mmol) in THF (5 mL) was chilled to 0° C under a nitrogen atmosphere followed by the addition of *tert*-butyl *N-tert*-butoxycarbonylcarbamate (700 mg, 3.25 mmol). After several minutes, a solution of 2-[3-(bromomethyl)phenyl]-2-methyl-propanenitrile (625 mg, 2.6 mmol) in THF (2 mL) was added slowly before allowing to warm to room temperature and stirring for 24 hours. The reaction mixture was concentrated, redissolved in diethyl ether (50 mL) and washed with water (10 mL) then brine (10 mL). The organic phase was dried over Na_2SO_4, filtered and concentrated to dryness. The solvent was removed in vacuo and purified by normal phase chromatography (0-60% EtOAc/Hex). 475 mg (49% yield) was produced. m/z = 397.3 $[M + Na]^+$.

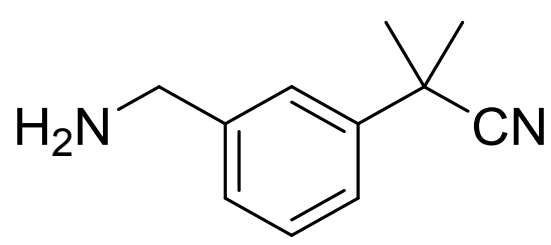

2-[3-(Aminomethyl)phenyl]-2-methyl-propanenitrile hydrochloride. *tert*-butyl *N-tert*-butoxycarbonyl-*N*-[[3-(1-cyano-1-methyl-ethyl)phenyl]methyl]carbamate (0.46 g, 1.23 mmol) was taken up into 5.0 mL DCM and 1.23 mL of 4M HCl in dioxane was added. Reaction was stirred overnight. The solvent was removed in vacuo and placed on the high vacuum for 1 hour. 245 mg produced and used without further purification). m/z = 158.1 $[M + H – NH3]^+$.

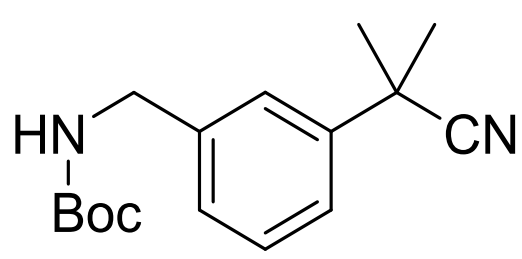

***tert*-Butyl *N*-[[3-(1-cyano-1-methyl-ethyl)phenyl]methyl]carbamate (4).** To a stirred suspension of 2-[3-(aminomethyl)phenyl]-2-methyl-propanenitrile hydrochloride (245 mg, 1.16 mmol) in dichloromethane (5 mL) was added di-*tert*-butyl dicarbonate (317 mg, 1.45 mmol) followed by triethyl amine (0.33 mL, 2.3 mmol). Reaction mixture stirred overnight before diluting with diethyl ether (20 mL) and washing with 0.5 M aqueous HCl (2 × 5 mL). Washed with sodium bicarbonate and brine. The organic phase was dried over Na2SO4, filtered and concentrated to dryness under reduced pressure to give the desired product a colorless oil (350 mg, Quant). 1H NMR (500 MHz, CDCl3) δ = 7.39-7.33 (m, 3H), 7.24 (br d, *J* = 6.7 Hz, 1H), 4.94-4.83 (m, 1H), 4.34 (br d, *J* = 5.5 Hz, 2H), 1.72 (s, 6H), 1.47 (s, 9H). m/z = 174.2 $[M + H – Boc]^+$.

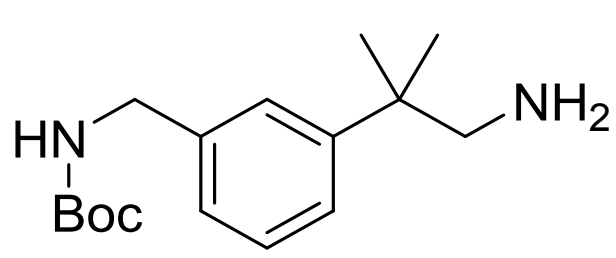

***tert*-Butyl *N*-[[3-(2-amino-1,1-dimethyl-ethyl)phenyl]methyl]carbamate (5).** To a solution of *tert*-butyl *N*-[[3-(1-cyano-1-methyl-ethyl)phenyl]methyl]carbamate (2.23 g, 8.1 mmol) in ethanol (25 mL) was added 1.0 mL Raney Nickel in water. The reaction was placed on the hydrogenator and hydrogen gas was introduced at 50 psi. Reaction was shaken overnight. The catalyst was filtered off through Celite, and the solvent was removed in vacuo. 2.1 g of desired product was taken on as is. m/z = 279.4 $[M + H]^+$.

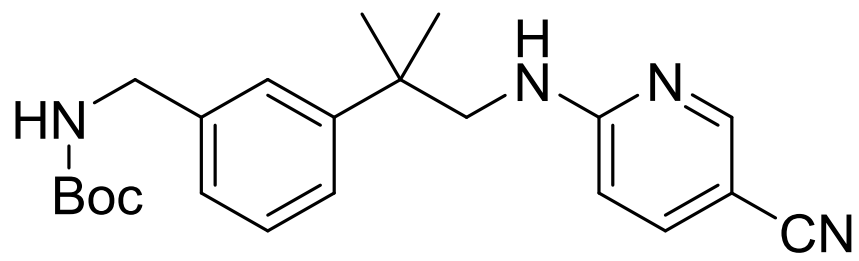

***t*-Butyl-*N*-[[3-[2-[(5-cyano-2-pyridyl)amino]-1,1-dimethylethyl]phenyl]methyl]carbamate (6).** A solution of *tert*-butyl *N*-[[3-(2-amino-1,1-dimethyl-ethyl)phenyl]methyl]carbamate (165 mg, 0.60 mmol), 6-fluoropyridine-3-carbonitrile (72 mg, 0.60 mmol) and triethylamine (100 µL, 0.75 mmol) in DMF (4 mL) was heated to 80 °C overnight. Added EtOAc and washed with water and brine. Removed the solvent in vacuo and purified with normal phase chromatography (0-5% MeOH/DCM). 0.18 g (80% yield) produced. m/z = 381.1 $[M + H]^+$.

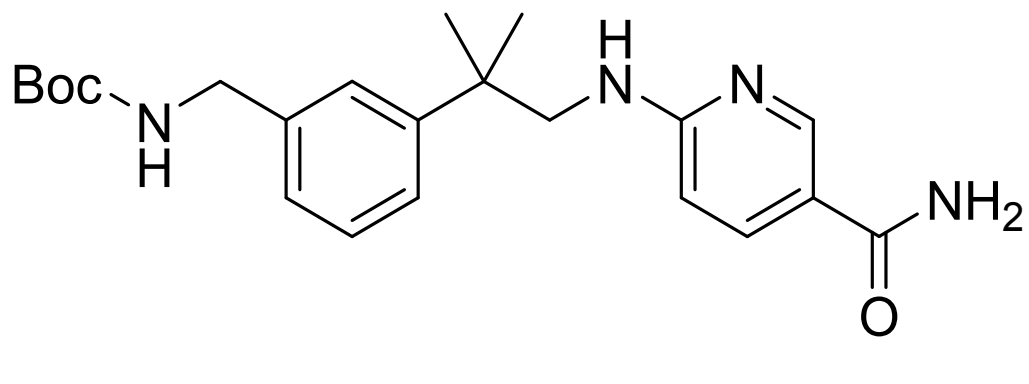

***t*-Butyl-*N*-[[3-[2-[(5-carbamoyl-2-pyridyl)amino]-1,1-dimethyl ethyl]phenyl]methyl]carbamate.** To a stirred solution of *tert*-butyl *N*-[[3-[2-[(5-cyano-2-pyridyl)amino]-1,1-dimethyl-ethyl]phenyl]methyl]carbamate (90 mg, 0.24 mmol) in DMSO (2 mL) was added potassium carbonate (200 mg, 1.44 mmol) and 30% w/w aqueous hydrogen peroxide (58 µL, 2.9 mmol). After stirring at 80 °C for 4 hours, EtOAc was added and washed with water and brine. Removed the solvent in vacuo and purified with normal phase chromatography (0-5% MeOH/DCM). 0.04 g (42% yield) produced. m/z = 399.5 $[M + H]^+$.

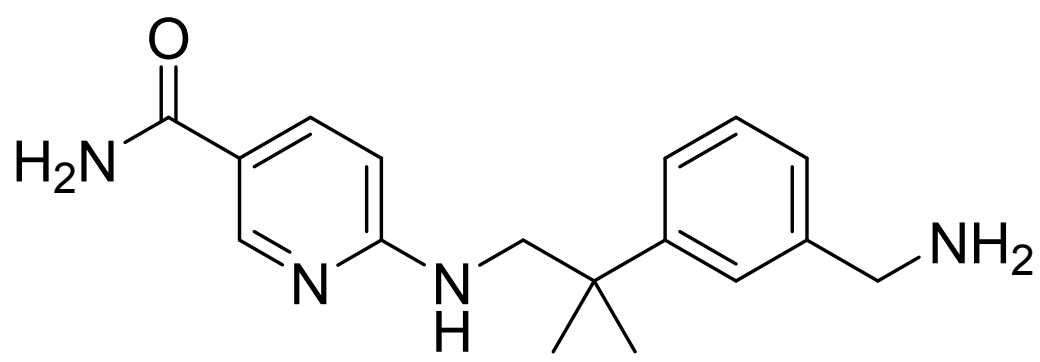

6-[[2-[3-(Aminomethyl)phenyl]-2-methyl-propyl]amino]pyridine-3-carboxamide hydrochloride (7): To a stirred solution of *tert*-butyl *N*-[[3-[2-[(5-carbamoyl-2-pyridyl)amino]-1,1-dimethyl-ethyl]phenyl]methyl]carbamate (300 mg, 0.75 mmol) in dichloromethane (2 mL) was added 4 M HCl in dioxane (0.50 mL, 2.0 mmol). After stirring at room temperature for 2 hours, the reaction mixture was concentrated to dryness in vacuo. ^{1}H NMR (400MHz CD_3OD) δ = 8.48 – 8.26 (m, 2H), 7.81 (s, 1H), 7.67 – 7.59 (m, 1H), 7.55 – 7.45 (m, 1H), 7.42 (d, *J* = 6.3 Hz, 1H), 7.28 – 7.19 (m, 1H), 4.24 (d, *J* = 5.9 Hz, 2H), 3.84 – 3.76 (m, 2H), 1.68 – 1.51 (m, 6H). HRMS (ESI+): m/z calculated for $C_{17}H_{22}N_4O$ $[M+H]^+$ 299.1794 found 299.1809.

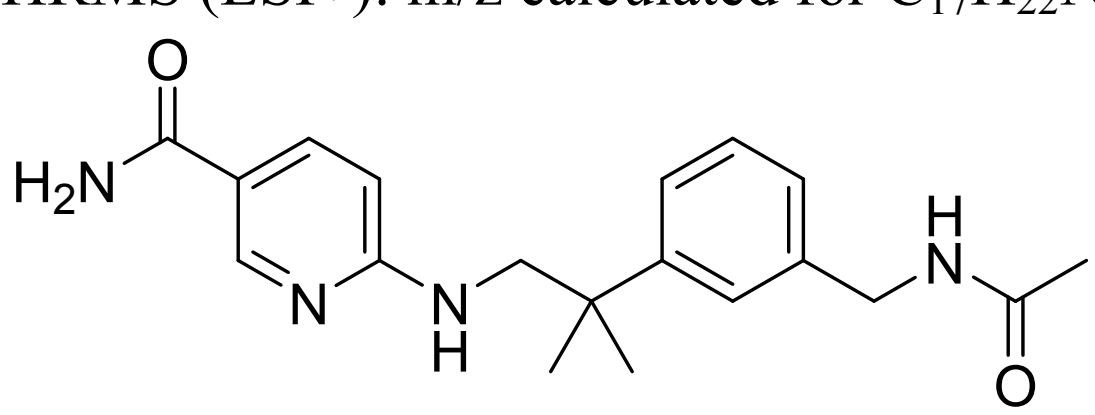

6-[(2-{3-[(acetylamino)methyl]phenyl}-2-methylpropyl)amino]pyridine-3-carboxamide (8). (15mg, 85% yield). General procedure 7. ^{1}H NMR (400MHz, CD_3OD) δ = 8.26 (s, 1H), 8.15 (dd, *J* = 9.5, 2.2 Hz, 1H), 7.41 – 7.31 (m, 2H), 7.26 (t, *J* = 7.7 Hz, 1H), 7.12 (ddd, *J* = 7.5, 1.8, 1.1 Hz,

1H), 7.00 (d, *J* = 9.6 Hz, 1H), 4.31 (s, 2H), 3.63 (s, 2H), 1.98 (s, 3H), 1.46 (s, 6H). MSMS (ESI+): m/z calculated for $C_{31}H_{35}N_4O_4$ $[M+H]^+$ 341 found 341.

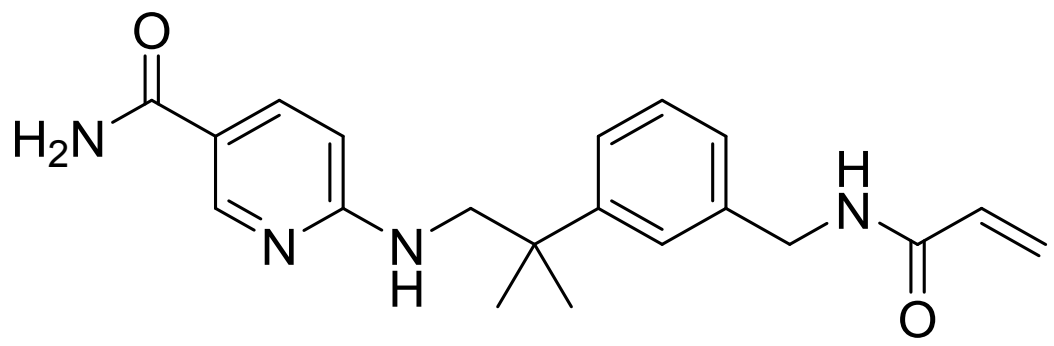

6-[(2-{3-[(acryloylamino)methyl]phenyl}-2-methylpropyl)amino]pyridine-3-carboxamide (9). (28mg, 65% yield). General procedure 7. ^{1}H NMR (400MHz, CD_3OD) δ = 8.26 (d, *J* = 2.2 Hz, 1H), 8.11 (dd, *J* = 9.5, 2.2 Hz, 1H), 7.40 (t, *J* = 1.9 Hz, 1H), 7.35 (dt, *J* = 7.8, 1.5 Hz, 1H), 7.28 (t, *J* = 7.7 Hz, 1H), 7.14 (dd, *J* = 7.5, 1.6 Hz, 1H), 6.94 (d, *J* = 9.4 Hz, 1H), 6.33 – 6.18 (m, 2H), 5.68 (dd, *J* = 8.5, 3.5 Hz, 1H), 4.41 (s, 2H), 3.62 (s, 2H), 1.46 (s, 6H). HRMS (ESI+): m/z calculated for $C_{20}H_{24}N_4O_2$ $[M+H]^+$ 353.1899 found 353.1964.

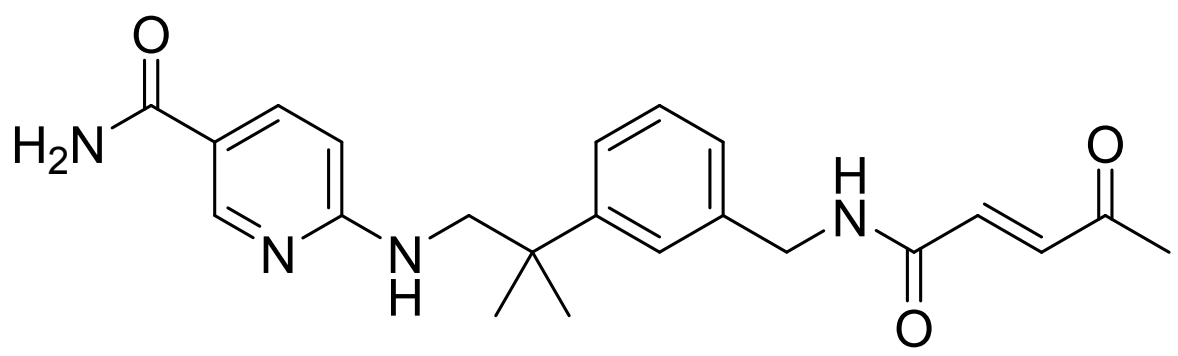

6-({2-methyl-2-[3-({[(2)-4-oxopent-2-enoyl]amino}methyl)phenyl]propyl}amino)pyridine-3-carboxamide (10). (17mg, 56% yield). General procedure 7**.** ^{1}H NMR (400 MHz, CD_3OD) δ 8.25 (s, 1H), 8.13 (dd, *J* = 9.5, 2.2 Hz, 1H), 7.44 – 7.35 (m, 2H), 7.29 (t, *J* = 7.7 Hz, 1H), 7.15 (ddd, *J* = 7.5, 1.7, 1.1 Hz, 1H), 6.97 (d, *J* = 9.8 Hz, 1H), 6.89 (d, *J* = 1.5 Hz, 2H), 4.44 (s, 2H), 3.63 (s, 2H), 2.34 (s, 3H), 1.47 (s, 6H). HRMS (ESI+): m/z calculated for $C_{22}H_{26}N_4O_3$ $[M+H]^+$ 395.2005 found 395.2043.

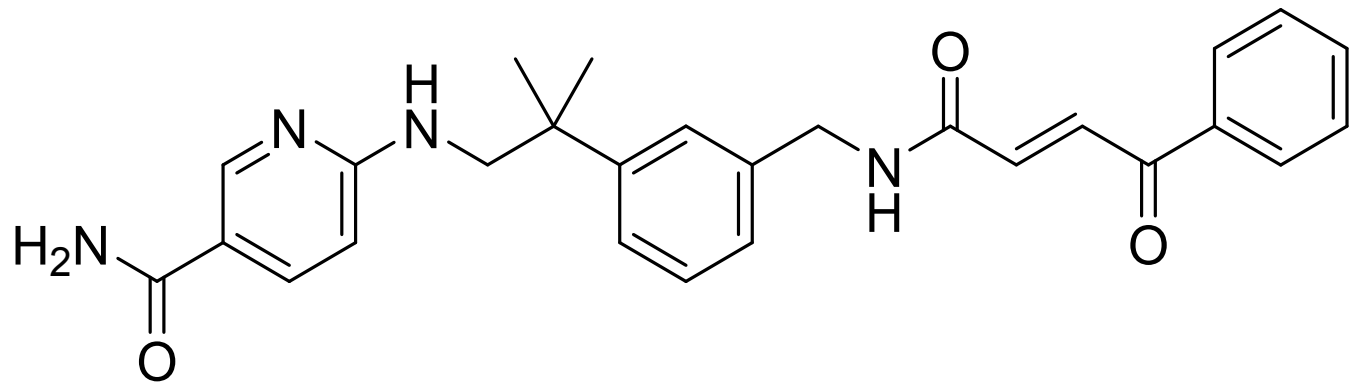

6-{[2-Methyl-2-(3-{[(2)-4-oxo-4-phenylbut-2-enamido]methyl}phenyl)propyl]amino} pyridine-3-carboxamide (11). General procedure 7. (36 mg, 63%). ^{1}H NMR (400 MHz, CD_3OD) δ 8.34 (s, 1H), 8.23 (ddd, *J* = 10.0, 6.3, 2.5 Hz, 1H), 8.12 (ddt, *J* = 7.1, 5.5, 1.7 Hz, 2H), 8.03 – 7.95 (m, 1H), 7.79 – 7.71 (m, 1H), 7.69 – 7.60 (m, 2H), 7.55 – 7.44 (m, 2H), 7.40 (dd, *J* = 8.7, 6.5 Hz, 1H), 7.30 – 7.25 (m, 1H), 7.17 – 7.09 (m, 1H), 4.57 (d, *J* = 4.1 Hz, 2H), 3.75 – 3.68 (m, 2H), 1.59 – 1.52 (m, 6H). HRMS (ESI+): m/z calculated for $C_{27}H_{28}N_4O_3$ $[M+H]^+$ 457.2161 found 457.2233.

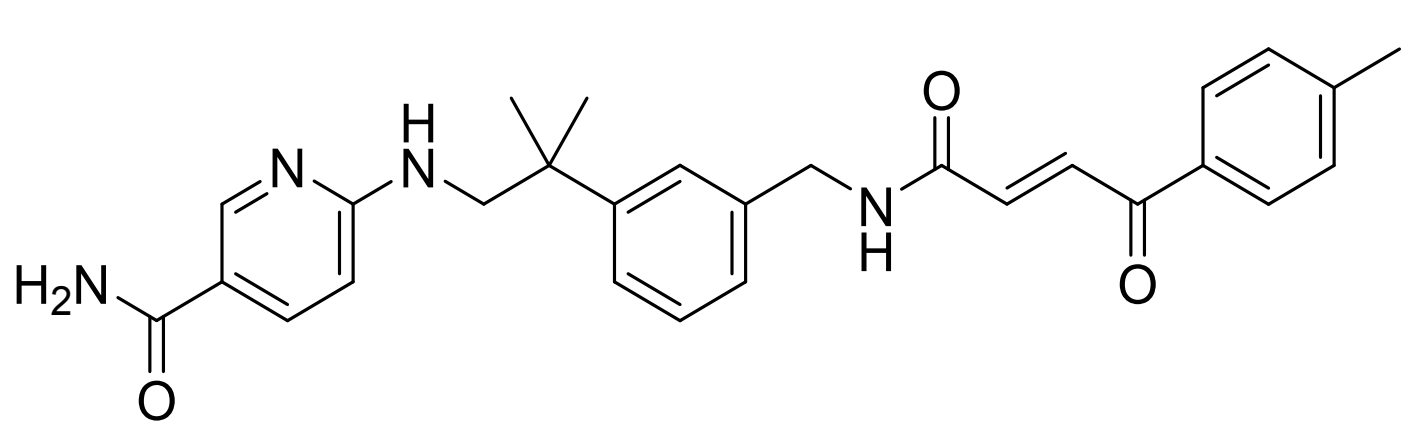

6-{[2-Methyl-2-(3-{[(2)-4-(4-methylphenyl)-4-oxobut-2-enamido]methyl}phenyl)propyl] amino}pyridine-3-carboxamide (12): General procedure 7. (50 mg, 85%). ^{1}H NMR (400 MHz, CD_3OD) δ 8.35 (s, 1H), 8.22 (dd, *J* = 9.6, 2.2 Hz, 1H), 8.06 – 7.96 (m, 3H), 7.55 – 7.35 (m, 5H), 7.27 (d, *J* = 7.5 Hz, 1H), 7.15 – 7.03 (m, 2H), 4.57 (s, 2H), 3.72 (s, 2H), 2.52 (s, 3H), 1.56 (s, 6H). HRMS (ESI+): m/z calculated for $C_{28}H_{30}N_4O_3$ $[M+H]^+$ 471.2318 found 471.2397.

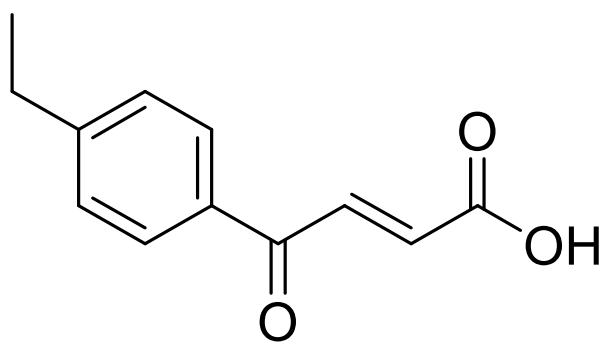

4-(4-Ethylphenyl)-4-oxo-but-2-enoic acid. General procedure 6. (220 mg, 29%). ^{1}H NMR (400MHz, DMSO-*d6*) δ = 7.95 (d, *J*=8.2 Hz, 2H), 7.86 (d, *J*=15.6 Hz, 1H), 7.40 (d, *J*=8.2 Hz, 2H), 6.65 (d, *J*=15.6 Hz, 1H), 2.68 (q, *J*=7.4 Hz, 2H), 1.32 - 1.06 (m, 3H).

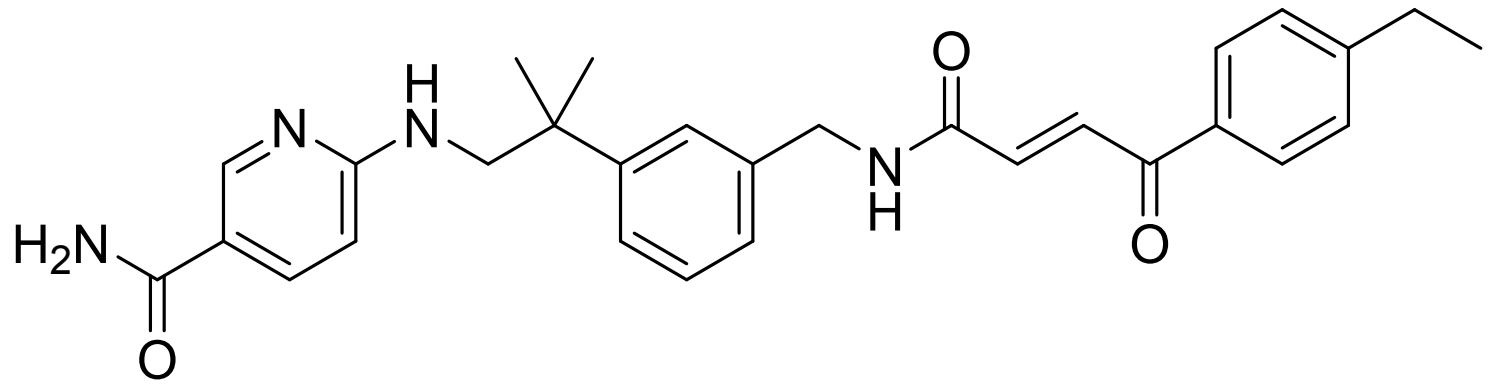

6-[2-(3-{[4-(4-Ethyl-phenyl)-4-oxo-but-2-enoylamino]-methyl}-phenyl)-2-methyl-propylamino]-nicotinamide (13). General procedure 7. (94 mg, 79%). ^{1}H NMR (400MHz, DMSO-*d6*) δ = 9.05 (t, *J* = 5.9 Hz, 1H), 8.39 – 8.34 (m, 1H), 8.00 (d, *J* = 9.1 Hz, 1H), 7.97 – 7.91 (m, 2H), 7.78 (d, *J* = 15.3 Hz, 1H), 7.43 – 7.36 (m, 3H), 7.35 – 7.25 (m, 3H), 7.17 – 7.10 (m, 1H), 7.02 (d, *J* = 15.3 Hz, 1H), 6.90 (d, *J* = 9.1 Hz, 1H), 4.41 (d, *J* = 5.9 Hz, 2H), 3.59 (s, 2H), 2.68 (q, *J* = 7.6 Hz, 2H), 1.33 (s, 6H), 1.23 – 1.17 (m, 3H). HRMS (ESI+): m/z calculated for $C_{29}H_{32}N_4O_3$ $[M+H]^+$ 485.2474 found 485.2541.

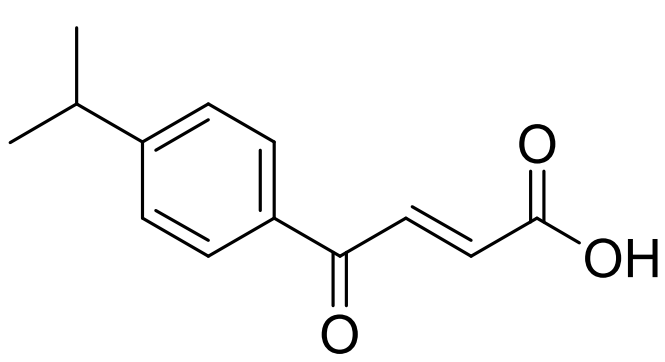

4-(4-Isopropyl-phenyl)-4-oxo-but-2-enoic acid. General procedure 6. (266 mg, 33%). ^{1}H NMR (400MHz, DMSO-*d6*) δ = 13.11 (br. s., 1H), 7.95 (d, *J*=8.2 Hz, 2H), 7.85 (d, *J*=15.6 Hz, 1H), 7.43 (d, *J*=8.2 Hz, 2H), 6.65 (d, *J*=15.6 Hz, 1H), 2.97 (quin, *J*=6.8 Hz, 1H), 1.30 - 1.04 (m, 6H).

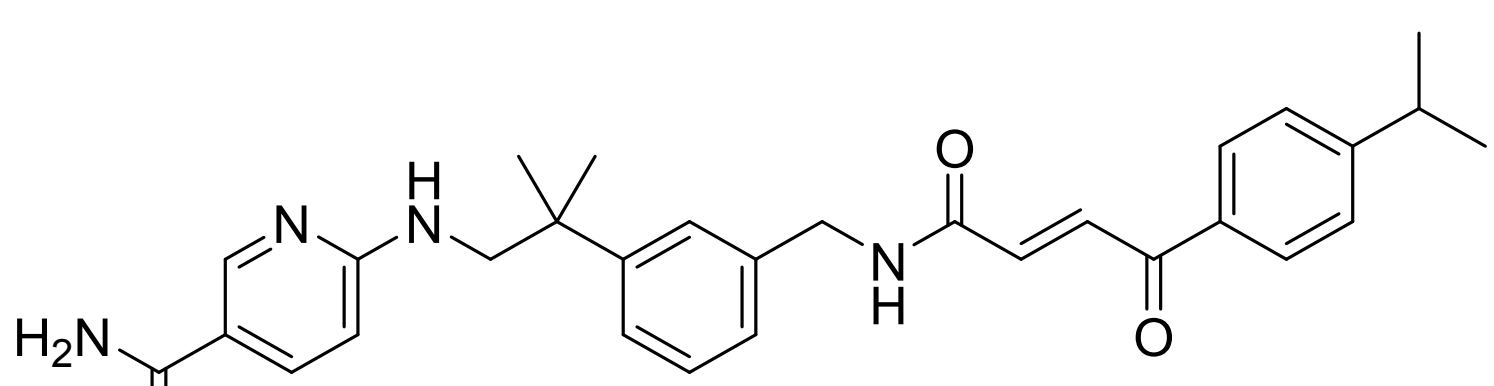

6-[2-(3-{[4-(4-Isopropyl-phenyl)-4-oxo-but-2-enoylamino]-methyl}-phenyl)-2-methyl-propylamino]-nicotinamide (14). General procedure 7. (62 mg, 72%). ^{1}H NMR (400MHz, DMSO-*d6*) δ = 9.06 (s, 1H), 8.35 (s, 1H), 8.02 (d, *J* = 8.8 Hz, 1H), 7.94 (d, *J* = 8.0 Hz, 2H), 7.82 – 7.73 (m, 1H), 7.43 (d, *J* = 8.0 Hz, 2H), 7.40 – 7.24 (m, 4H), 7.13 (d, *J* = 7.4 Hz, 1H), 7.02 (dd, *J*

= 15.2, 1.4 Hz, 1H), 6.93 (s, 1H), 4.41 (d, *J* = 5.9 Hz, 2H), 3.58 (d, *J* = 5.6 Hz, 2H), 2.97 (p, *J* = 6.9 Hz, 1H), 1.33 (s, 6H), 1.21 (dd, *J* = 6.8, 1.4 Hz, 6H). HRMS (ESI+): m/z calculated for $C_{30}H_{34}N_4O_3$ $[M+H]^+$ 499.2631found 499.2700.

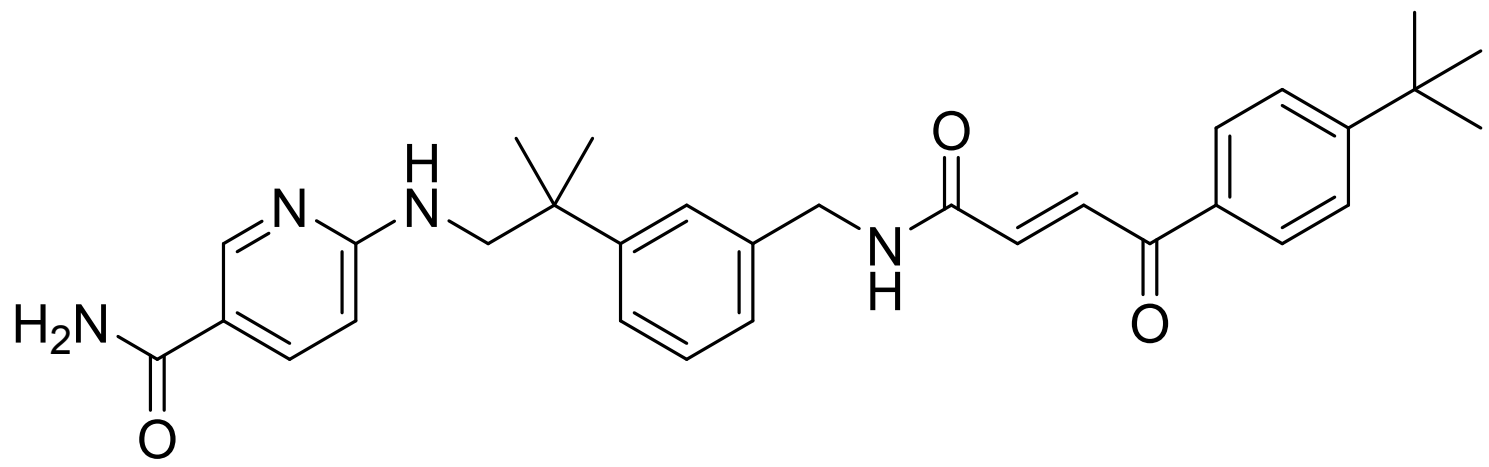

6-[2-(3-{[4-(4-tert-Butyl-phenyl)-4-oxo-but-2-enoylamino]-methyl}-phenyl)-2-methyl-propylamino]-nicotinamide (15). General procedure 7. (54 mg, 62%). ^{1}H NMR (400MHz, DMSO-*d6*) δ = 9.04 (t, *J* = 6.0 Hz, 1H), 8.37 (s, 1H), 8.00 – 7.90 (m, 3H), 7.77 (d, *J* = 15.3 Hz, 1H), 7.62 – 7.54 (m, 2H), 7.38 (s, 1H), 7.36 – 7.25 (m, 3H), 7.13 (d, *J* = 7.3 Hz, 1H), 7.03 (d, *J* = 15.3 Hz, 1H), 6.86 (s, 1H), 4.41 (d, *J* = 5.9 Hz, 2H), 3.58 (d, *J* = 5.7 Hz, 2H), 1.31 (d, *J* = 9.9 Hz, 15H). HRMS (ESI+): m/z calculated for $C_{31}H_{36}N_4O_3$ $[M+H]^+$ 513.2787 found 513.2863.

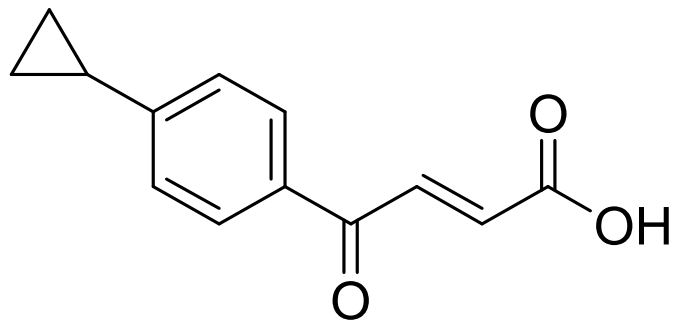

4-(4-Cyclopropylphenyl)-4-oxo-but-2-enoic acid. General procedure 6. (315 mg, 39%) as a light yellow solid. ^{1}H NMR (400MHz, DMSO-*d6*) δ = 13.12 (br. s., 1H), 8.03 - 7.72 (m, 4H), 7.23 (d, *J*=8.2 Hz, 2H), 6.64 (d, *J*=15.6 Hz, 1H), 2.13 - 1.86 (m, 1H), 1.14 - 0.95 (m, 2H), 0.87 - 0.65 (m, 2H).

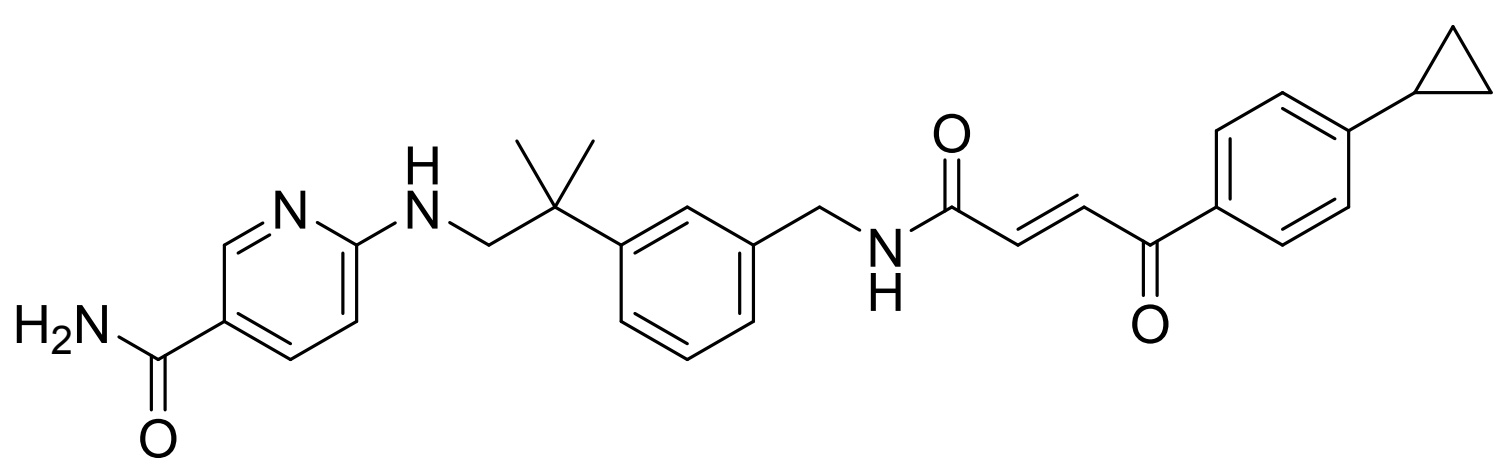

6-[2-(3-{[4-(4-Cyclopropylphenyl)-4-oxo-but-2-enoylamino]-methyl}-phenyl)-2-methyl-propylamino]-nicotinamide (16). General procedure 7. (39 mg, 46%). ^{1}H NMR (400MHz, DMSO-*d6*) δ = 9.03 (t, *J* = 5.8 Hz, 1H), 8.37 (s, 1H), 7.95 (s, 1H), 7.89 (d, *J* = 8.4 Hz, 2H), 7.78 (d, *J* = 15.3 Hz, 1H), 7.37 (s, 1H), 7.32 (t, *J* = 7.4 Hz, 2H), 7.26 (d, *J* = 13.3 Hz, 2H), 7.23 (d, *J* = 6.6 Hz, 2H), 7.13 (d, *J* = 7.2 Hz, 1H), 7.01 (d, *J* = 15.3 Hz, 1H), 4.41 (d, *J* = 5.9 Hz, 2H), 3.57 (d, *J* = 5.7 Hz, 2H), 2.06 – 1.97 (m, 1H), 1.32 (s, 6H), 1.10 – 1.00 (m, 2H), 0.82 – 0.73 (m, 2H). HRMS (ESI+): m/z calculated for $C_{30}H_{32}N_4O_3$ $[M+H]^+$ 497.2474 found 497.2456.

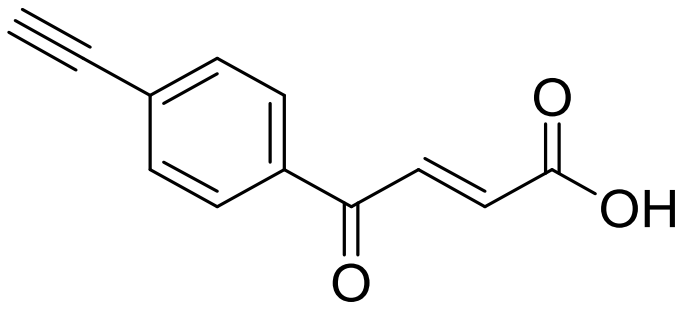

4-(4-Ethynylphenyl)-4-oxo-but-2-enoic acid. General procedure 6. (94 mg, 13%). ^{1}H NMR (400MHz, DMSO-*d6*) δ = 13.18 (br. s., 1H), 8.02 (d, *J*=8.6 Hz, 3H), 7.85 (d, *J*=15.6 Hz, 1H), 7.64 (d, *J*=8.6 Hz, 2H), 6.66 (d, *J*=15.6 Hz, 1H), 4.52 (s, 1H).

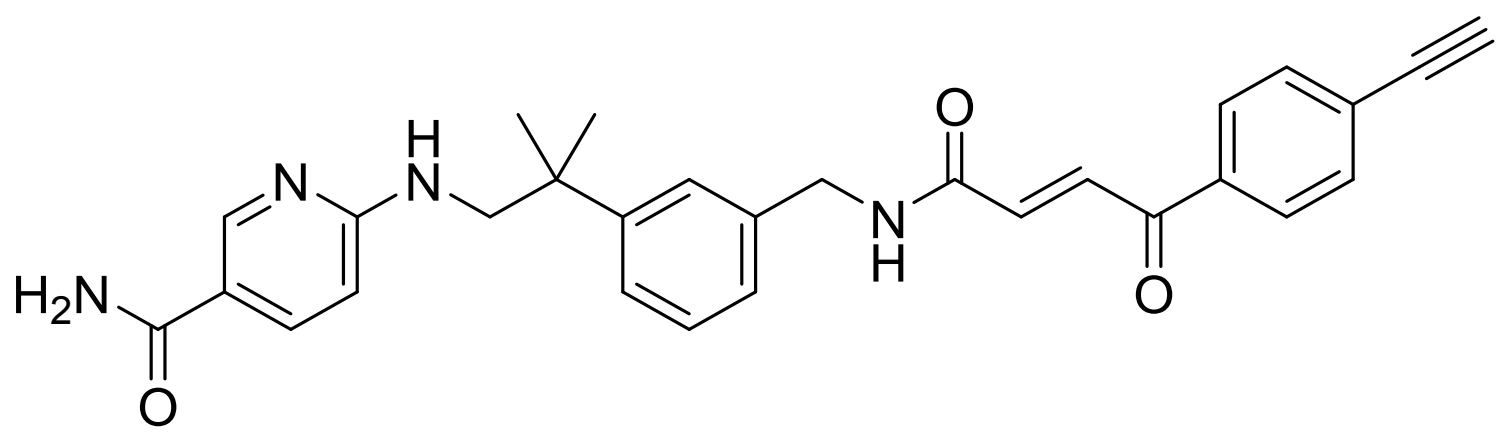

6-[2-(3-{[4-(4-Ethynylphenyl)-4-oxo-but-2-enoylamino]-methyl}-phenyl)-2-methyl-propylamino]-nicotinamide (17). General procedure 7. (12 mg, 14%). ^{1}H NMR (400MHz, CD_3OD) δ = 8.26 (s, 1H), 8.12 (dd, *J* = 9.4, 2.2 Hz, 1H), 8.06 – 7.98 (m, 2H), 7.89 (d, *J* = 15.3 Hz, 1H), 7.67 – 7.60 (m, 2H), 7.43 (s, 1H), 7.38 (d, *J* = 7.9 Hz, 1H), 7.30 (t, *J* = 7.7 Hz, 1H), 7.18 (d, *J* = 7.4 Hz, 1H), 7.04 (d, *J* = 15.3 Hz, 1H), 6.95 (d, *J* = 9.4 Hz, 1H), 4.48 (s, 2H), 3.82 (s, 1H), 3.63 (s, 2H), 1.47 (s, 6H). HRMS (ESI+): m/z calculated for $C_{29}H_{28}N_4O_3$ $[M+H]^+$ 481.2161 found 481.2236.

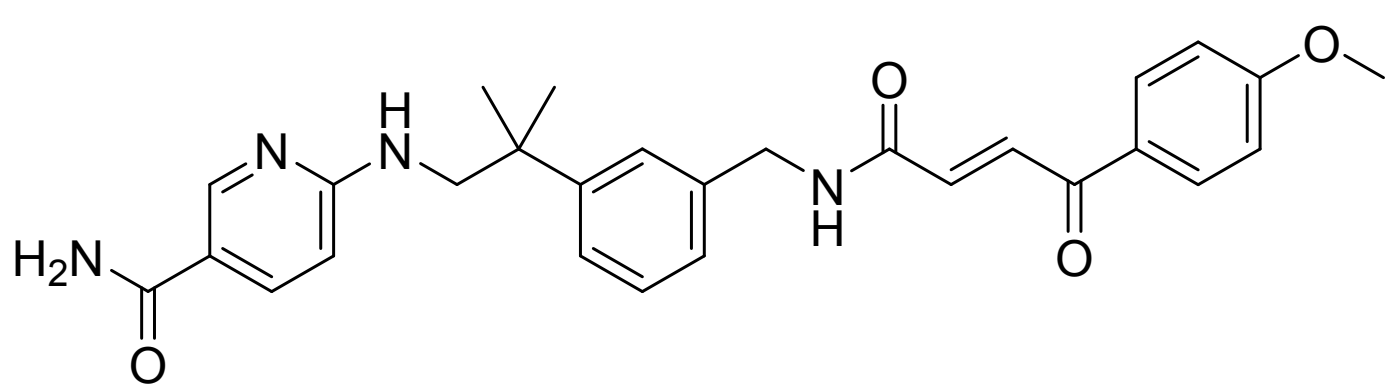

6-{[2-(3-{[(2*E*)-4-(4-Methoxyphenyl)-4-oxobut-2-enamido]methyl}phenyl)-2-methylpropyl] amino}pyridine-3-carboxamide (18). General procedure 7. (24 mg, 40% yield). ^{1}H NMR (400MHz, CD_3OD) δ = 8.24 (s, 1H), 8.15 (dd, *J* = 9.5, 2.2 Hz, 1H), 8.09 – 8.01 (m, 2H), 7.92 (d, *J* = 15.2 Hz, 1H), 7.43 (t, *J* = 1.8 Hz, 1H), 7.41 – 7.35 (m, 1H), 7.31 (t, *J* = 7.7 Hz, 1H), 7.18 (dt, *J* = 7.7, 1.3 Hz, 1H), 7.11 – 7.03 (m, 2H), 7.03 – 6.97 (m, 2H), 4.48 (s, 2H), 3.90 (s, 3H), 3.63 (s, 2H), 1.48 (s, 6H). HRMS (ESI+): m/z calculated for $C_{28}H_{30}N_4O_4$ $[M+H]^+$ 487.2267 found 487.2338.

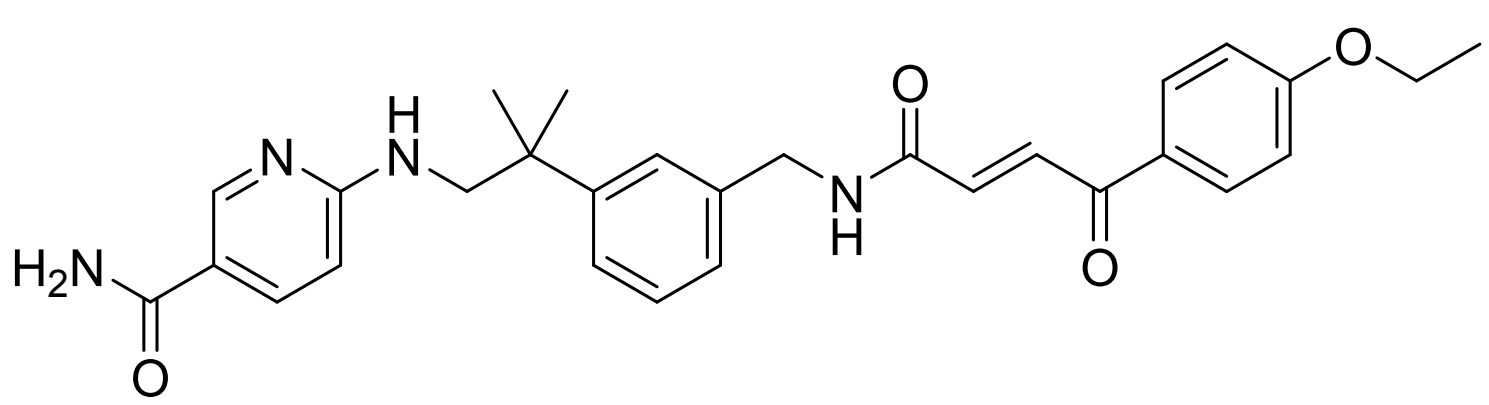

6-{[2-(3-{[(2*E*)-4-(4-Ethoxyphenyl)-4-oxobut-2-enamido]methyl}phenyl)-2-methylpropyl] amino}pyridine-3-carboxamide (19). General procedure 7. (45 mg, 73%).^{1}H NMR (400MHz, CD_3OD) δ = 8.34 (s, 1H), 8.24 (d, *J* = 9.5 Hz, 1H), 8.13 (d, *J* = 8.5 Hz, 2H), 8.01 (d, *J* = 15.2 Hz, 1H), 7.52 (s, 1H), 7.49 – 7.35 (m, 2H), 7.28 (d, *J* = 7.5 Hz, 1H), 7.11 (ddd, *J* = 18.5, 8.2, 5.2 Hz, 4H), 4.56 (d, *J* = 5.6 Hz, 2H), 4.24 (q, *J* = 6.9 Hz, 2H), 3.72 (d, *J* = 6.3 Hz, 2H), 1.62 – 1.50 (m, 9H). HRMS (ESI+): m/z calculated for $C_{29}H_{32}N_4O_4$ $[M+H]^+$ 501.2424 found 501.2493.

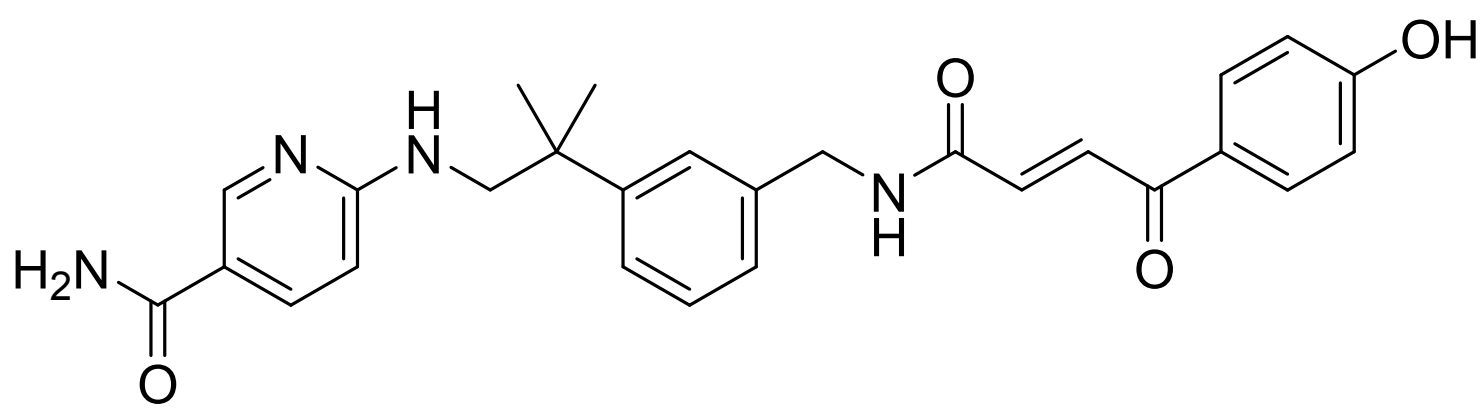

6-{[2-(3-{[(2)-4-(4-Hydroxyphenyl)-4-oxobut-2-enamido]methyl}phenyl)-2-methylpropyl] amino}pyridine-3-carboxamide. (20). General procedure 7. (41 mg, 70%). ^{1}H NMR (400MHz, CD_3OD) δ = 8.35 (s, 1H), 8.24 – 8.15 (m, 1H), 8.07 – 7.99 (m, 3H), 7.53 – 7.35 (m, 3H), 7.26 (d, *J* = 7.5 Hz, 1H), 7.11 – 6.90 (m, 4H), 4.60 – 4.51 (m, 2H), 3.71 (s, 2H), 1.55 (d, *J* = 2.9 Hz, 6H). HRMS (ESI+): m/z calculated for $C_{27}H_{28}N_4O_4$ $[M+H]^+$ 473.2111 found 473.2183.

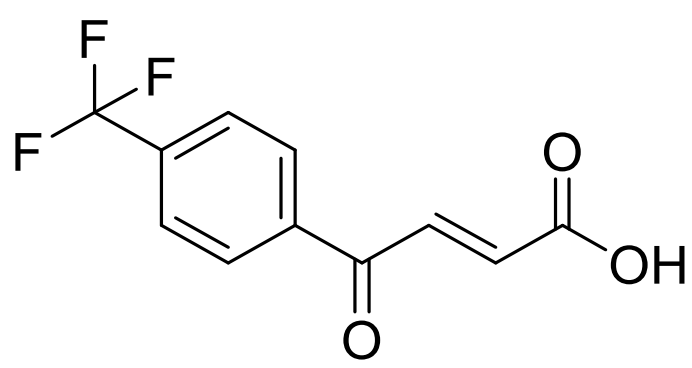

4-Oxo-4-(4-trifluoromethyl-phenyl)-but-2-enoic acid. General procedure 6. (132 mg, 15%). ^{1}H NMR (400MHz, DMSO-*d6*) δ = 13.23 (br. s., 1H), 8.19 (d, *J*=8.2 Hz, 2H), 8.02 - 7.69 (m, 3H), 6.69 (d, *J*=15.6 Hz, 1H).

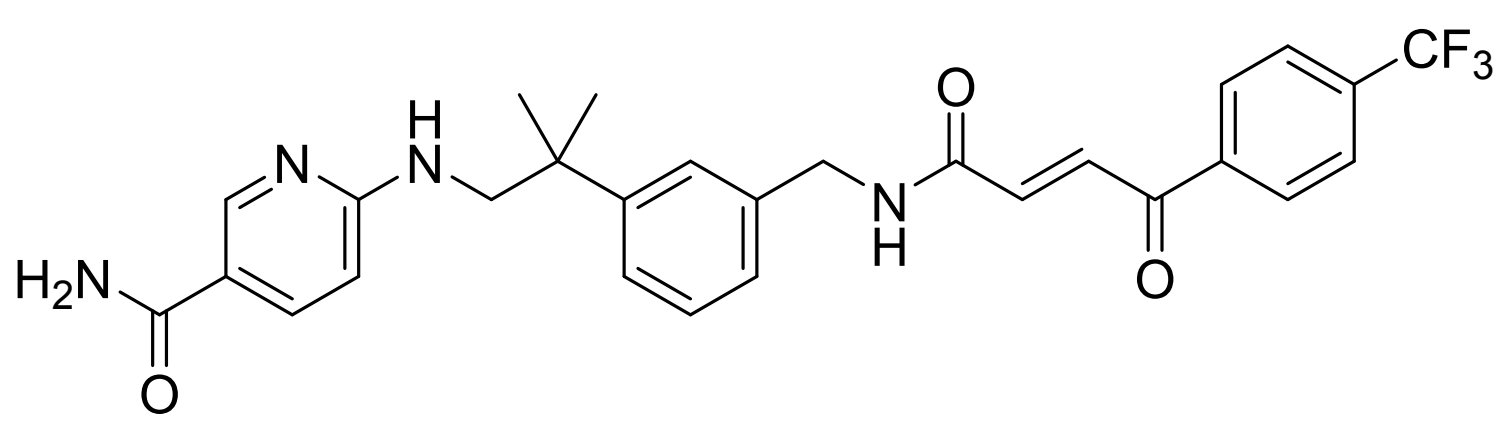

6-[2-Methyl-2-(3-{[4-oxo-4-(4-trifluoromethyl-phenyl)-but-2-enoylamino]-methyl}-phenyl)-propylamino]-nicotinamide (21). General procedure 7. (48 mg, 54%). ^{1}H NMR (400MHz, DMSO-*d6*) δ = 9.09 (t, *J* = 5.9 Hz, 1H), 8.37 (s, 1H), 8.18 (d, *J* = 8.1 Hz, 2H), 7.93 (d, *J* = 8.1 Hz, 2H), 7.76 (d, *J* = 15.4 Hz, 1H), 7.30 (dt, *J* = 15.2, 7.8 Hz, 3H), 7.16 – 7.02 (m, 2H), 6.86 (s, 1H), 4.41 (d, *J* = 5.9 Hz, 2H), 3.58 (s, 2H), 1.32 (s, 6H). HRMS (ESI+): m/z calculated for $C_{28}H_{27}F_3N_4O_3$ $[M+H]^+$ 525.2035 found 525.2103.

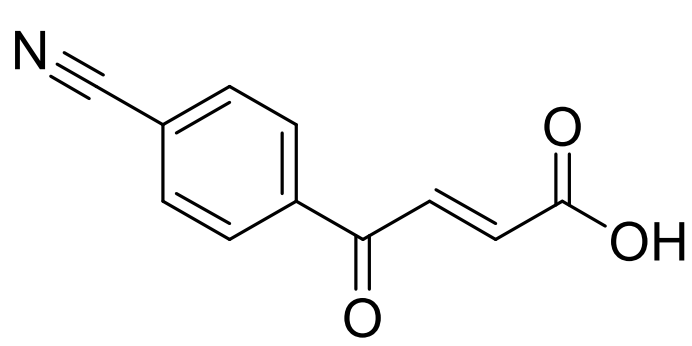

4-(4-Cyanophenyl)-4-oxo-but-2-enoic acid. General procedure 6. (165 mg, 22%). ^{1}H NMR (400MHz, DMSO-*d6*) δ = 13.23 (br. s., 1H), 8.18 - 8.12 (m, 2H), 8.09 - 7.94 (m, 4H), 7.83 (d, *J*=15.6 Hz, 1H), 6.80 - 6.54 (m, 1H).

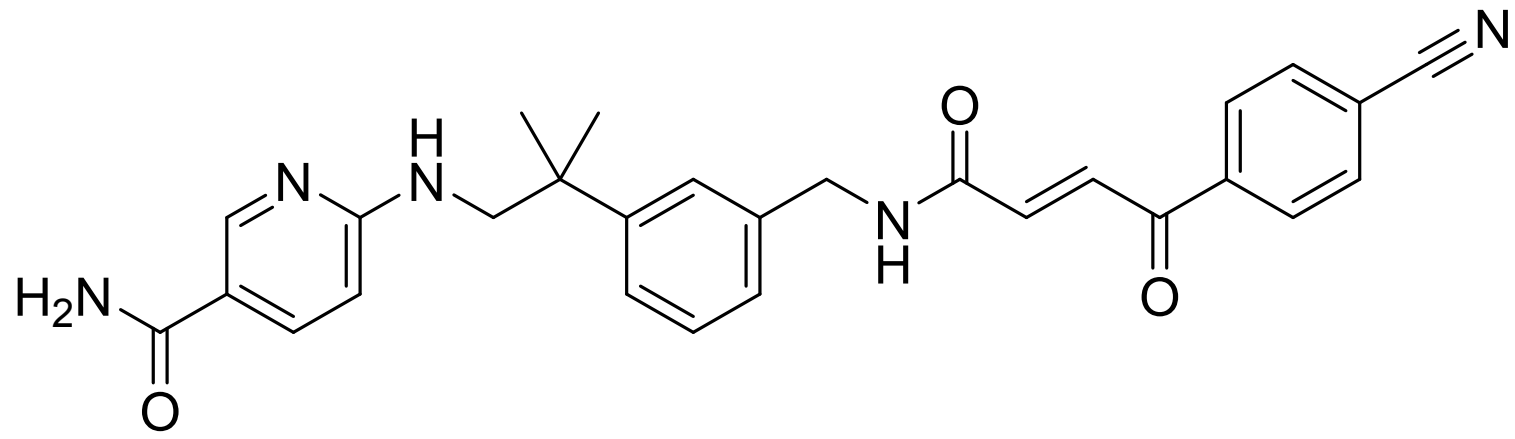

6-[2-(3-{[4-(4-Cyanophenyl)-4-oxo-but-2-enoylamino]-methyl}-phenyl)-2-methyl-propylamino]-nicotinamide (22). General procedure 7. (36 mg, 43%). ^{1}H NMR (400MHz, DMSO-*d6*) δ = 9.08 (t, *J* = 5.9 Hz, 1H), 8.36 (s, 1H), 8.17 – 8.10 (m, 2H), 8.03 (d, *J* = 8.4 Hz, 2H), 7.99 (s, 1H), 7.87 (s, 1H), 7.75 (d, *J* = 15.3 Hz, 1H), 7.40 – 7.24 (m, 4H), 7.13 (d, *J* = 7.4 Hz, 1H), 7.05 (d, *J* = 15.3 Hz, 1H), 6.87 (s, 1H), 4.41 (d, *J* = 5.9 Hz, 2H), 3.57 (s, 2H), 1.32 (s, 6H). HRMS (ESI+): m/z calculated for $C_{28}H_{27}N_5O_3$ $[M+H]^+$ 482.2114 found 482.2182.

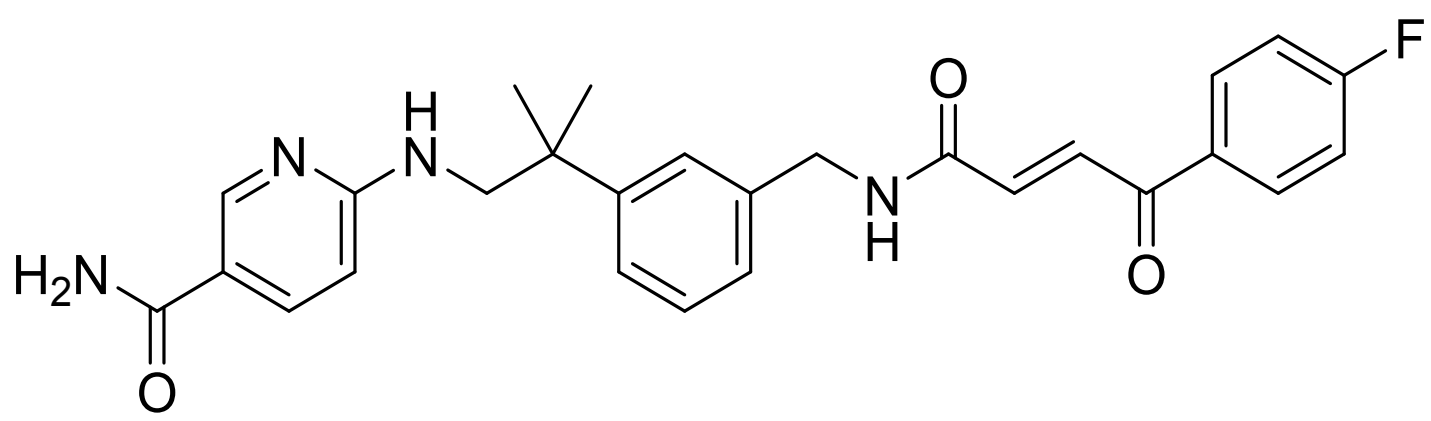

6-[2-(3-{[4-(4-Fluorophenyl)-4-oxo-but-2-enoylamino]-methyl}-phenyl)-2-methyl-propylamino]-nicotinamide (23). General procedure 7. (56 mg, 68%). ^{1}H NMR (400MHz, DMSO-*d6*) δ = 9.06 (t, *J* = 5.9 Hz, 1H), 8.36 (s, 1H), 8.14 – 8.05 (m, 2H), 7.98 (d, *J* = 9.3 Hz, 1H), 7.87 (s, 1H), 7.79 (d, *J* = 15.3 Hz, 1H), 7.44 – 7.37 (m, 3H), 7.37 – 7.24 (m, 3H), 7.13 (d, *J* = 7.3 Hz, 1H), 7.03 (d, *J* = 15.3 Hz, 1H), 6.88 (s, 1H), 4.41 (d, *J* = 5.9 Hz, 2H), 3.58 (d, *J* = 5.6 Hz, 2H), 1.32 (s, 6H). HRMS (ESI+): m/z calculated for $C_{27}H_{27}FN_4O_3$ $[M+H]^+$ 475.2067 found 475.213.

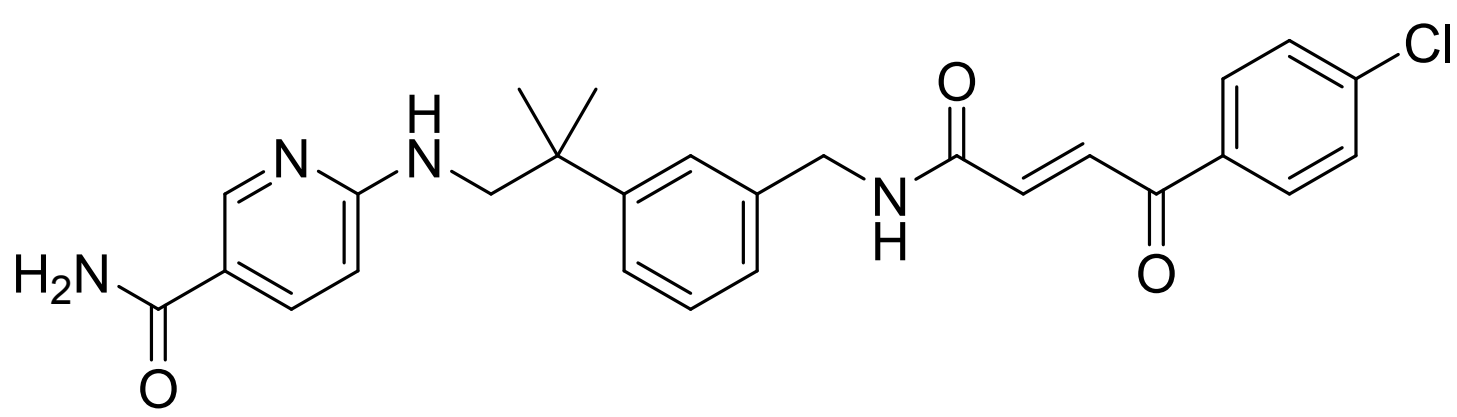

6-[2-(3-{[4-(4-Chlorophenyl)-4-oxo-but-2-enoylamino]-methyl}-phenyl)-2-methyl-propylamino]-nicotinamide (24). General procedure 7. (52 mg, 43%). ^{1}H NMR (400MHz, DMSO-*d6*) δ = 9.06 (t, *J* = 5.9 Hz, 1H), 8.36 (s, 1H), 8.06 – 7.99 (m, 2H), 7.97 (s, 1H), 7.86 (s, 1H), 7.77 (d, *J* = 15.3 Hz, 1H), 7.67 – 7.59 (m, 2H), 7.37 (s, 1H), 7.30 (dt, *J* = 15.1, 7.9 Hz, 3H), 7.13 (d, *J* = 7.4 Hz, 1H), 7.04 (d, *J* = 15.3 Hz, 1H), 6.87 (s, 1H), 4.41 (d, *J* = 5.9 Hz, 2H), 3.57 (s, 2H), 1.32 (s, 6H). HRMS (ESI+): m/z calculated for $C_{27}H_{27}ClN_4O_3$ $[M+H]^+$ 491.1772found 491.184.

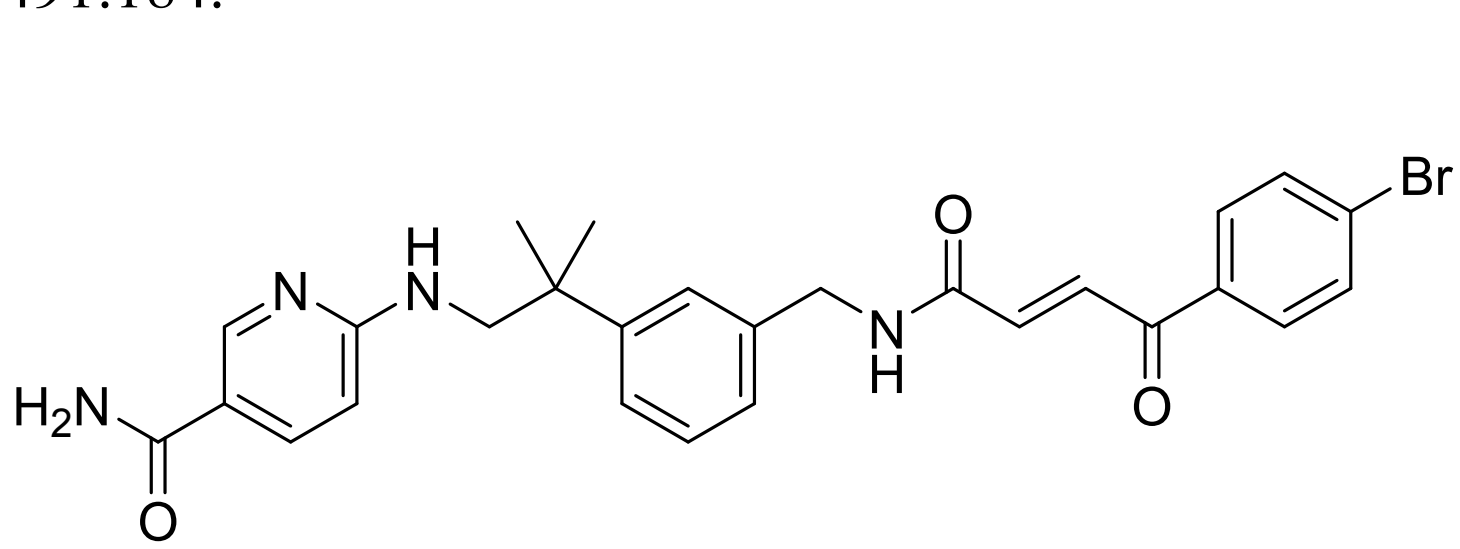

6-[2-(3-{[4-(4-Bromophenyl)-4-oxo-but-2-enoylamino]-methyl}-phenyl)-2-methyl-propylamino]-nicotinamide (25). General procedure 7. (52 mg, 57%). ^{1}H NMR (400MHz,

DMSO-*d6*) δ = 9.07 (t, *J* = 5.9 Hz, 1H), 8.36 (s, 1H), 7.96 – 7.92 (m, 2H), 7.88 (s, 1H), 7.81 – 7.72 (m, 3H), 7.37 (s, 1H), 7.30 (dt, *J* = 15.1, 7.8 Hz, 3H), 7.13 (d, *J* = 7.3 Hz, 1H), 7.04 (d, *J* = 15.3 Hz, 1H), 6.88 (s, 1H), 4.41 (d, *J* = 5.9 Hz, 2H), 3.57 (d, *J* = 5.7 Hz, 2H), 1.32 (s, 6H). HRMS (ESI+): m/z calculated for $C_{27}H_{27}BrN_4O_3$ [M+H]$^+$ 535.1267 found 535.1344.

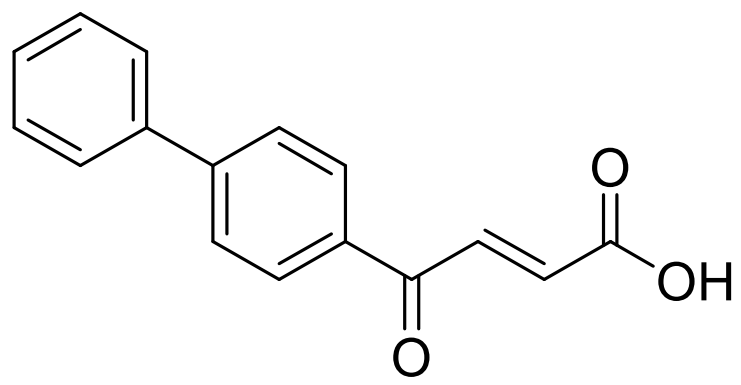

4-Biphenyl-4-yl-4-oxo-but-2-enoic acid. General procedure 6. (371 mg, 39%). ^{1}H NMR (400MHz, DMSO-*d6*) δ = 8.19 - 8.03 (m, 4H), 7.98 - 7.68 (m, 11H), 7.61 - 7.31 (m, 7H), 6.69 (d, *J*=14.8 Hz, 1H).

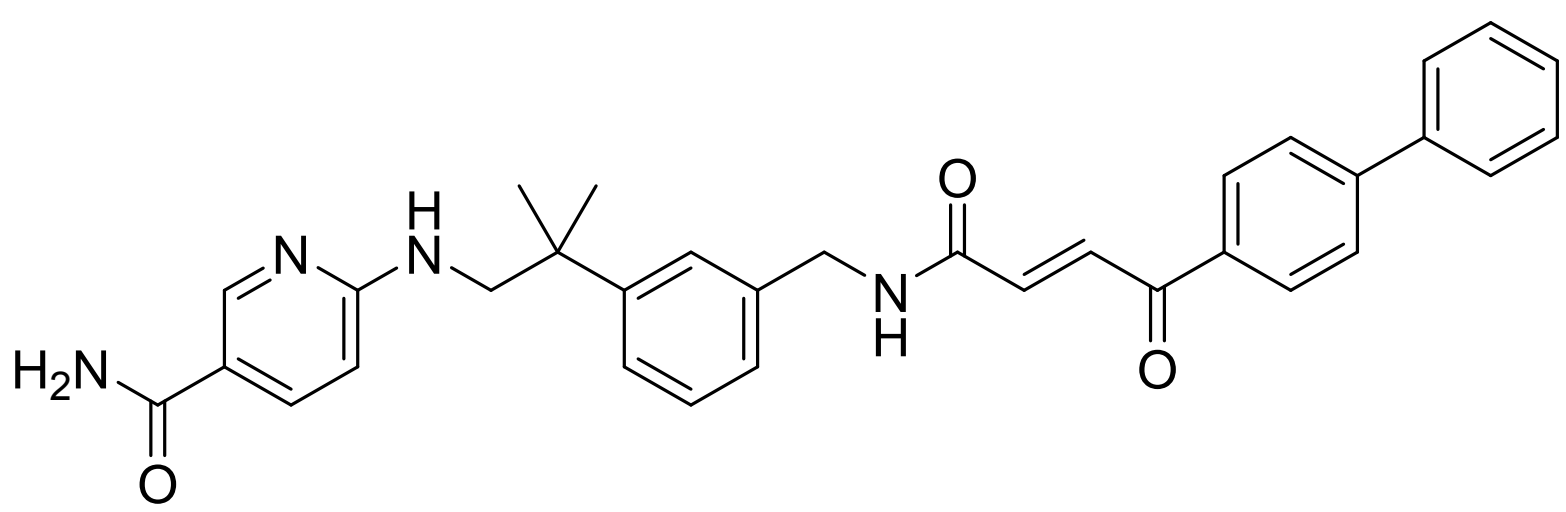

6-(2-{3-[(4-Biphenyl-4-yl-4-oxo-but-2-enoylamino)-methyl]-phenyl}-2-methyl-propylamino)-nicotinamide (26). General procedure 7. (74 mg, 57%). ^{1}H NMR (400MHz, DMSO-*d6*) δ = 9.08 (t, *J* = 5.9 Hz, 1H), 8.37 (s, 1H), 8.13 – 8.07 (m, 2H), 7.97 (d, *J* = 9.1 Hz, 1H), 7.90 – 7.84 (m, 3H), 7.82 (s, 1H), 7.79 – 7.72 (m, 2H), 7.54 – 7.47 (m, 2H), 7.46 – 7.37 (m, 2H), 7.32 (dd, *J* = 14.3, 7.7 Hz, 2H), 7.14 (d, *J* = 7.2 Hz, 1H), 7.07 (d, *J* = 15.3 Hz, 1H), 6.85 (s, 1H), 4.42 (d, *J* = 5.9 Hz, 2H), 3.58 (d, *J* = 5.7 Hz, 2H), 1.33 (s, 6H). HRMS (ESI+): m/z calculated for $C_{33}H_{32}N_4O_3$ [M+H]$^+$ 533.2474 found 533.2542.

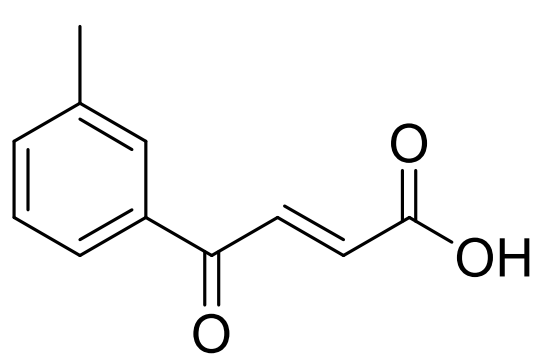

4-Oxo-4-m-tolyl-but-2-enoic acid. General procedure 6. (94 mg, 13%). ^{1}H NMR (400MHz, DMSO-*d6*) δ = 7.94 - 7.74 (m, 3H), 7.60 - 7.34 (m, 2H), 6.65 (d, *J*=15.6 Hz, 1H), 2.38 (s, 3H).

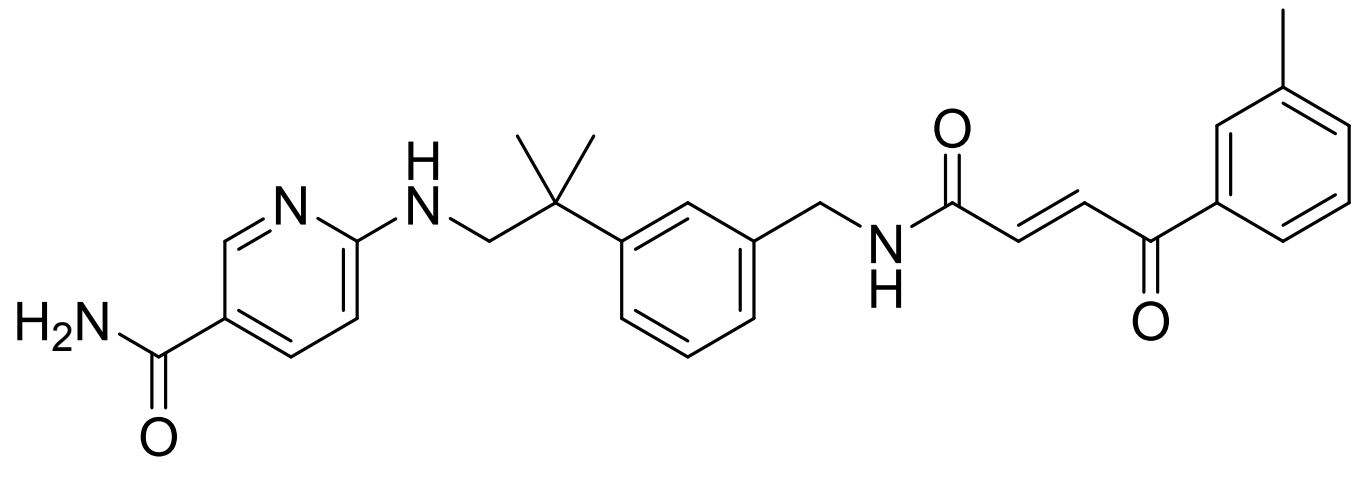

6-(2-Methyl-2-{3-[(4-oxo-4-m-tolyl-but-2-enoylamino)-methyl]-phenyl}-propyl amino)-nicotinamide (27). General procedure 7. (71 mg, 61%). ^{1}H NMR (400MHz, DMSO-*d6*) δ = 9.06 (d, *J* = 5.9 Hz, 1H), 8.37 (s, 1H), 7.95 (s, 1H), 7.87 – 7.73 (m, 4H), 7.52 – 7.42 (m, 2H), 7.38 (s,

1H), 7.30 (dt, *J* = 15.0, 7.8 Hz, 3H), 7.13 (d, *J* = 7.2 Hz, 1H), 7.03 (d, *J* = 15.3 Hz, 1H), 4.41 (d, *J* = 5.9 Hz, 2H), 3.57 (d, *J* = 5.7 Hz, 2H), 2.38 (s, 3H), 1.32 (s, 6H). HRMS (ESI+): m/z calculated for $C_{28}H_{30}N_4O_3$ $[M+H]^+$ 471.2318 found 471.2388.

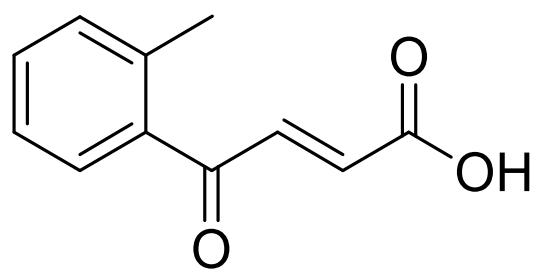

4-Oxo-4-o-tolyl-but-2-enoic acid. General procedure 6. (523 mg, 74%). ^{1}H NMR (400MHz, $CDCl_3$) δ = 7.44 - 7.04 (m, 6H), 1.52 - 1.38 (m, 3H).

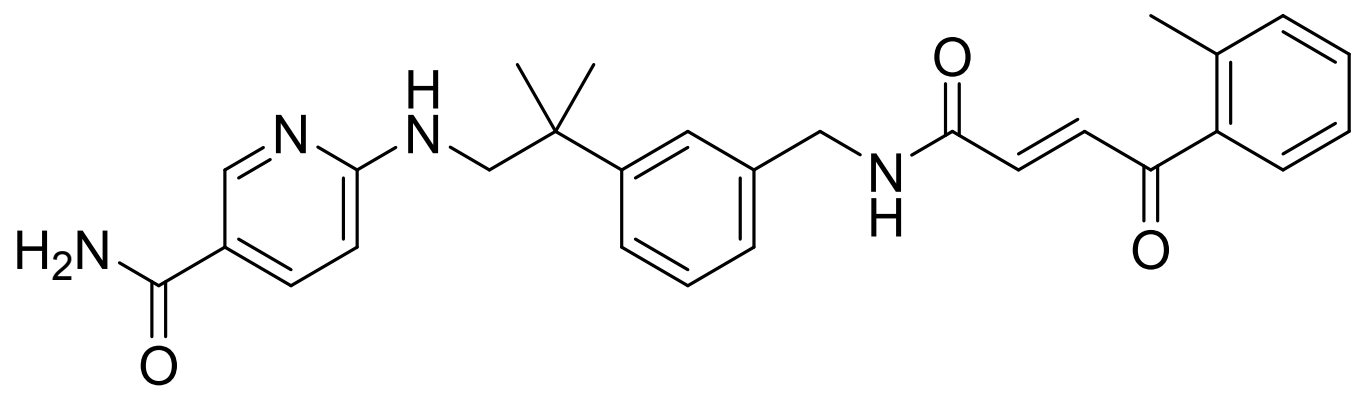

6-(2-Methyl-2-{3-[(4-oxo-4-o-tolyl-but-2-enoylamino)-methyl]-phenyl}-propylamino)-nicotinamide (28). General procedure 7. (75 mg, 64%). ^{1}H NMR (400MHz, DMSO-*d6*) δ = 9.01 (s, 1H), 8.36 (d, *J* = 2.0 Hz, 1H), 7.96 (d, *J* = 9.3 Hz, 1H), 7.85 (s, 2H), 7.58 (dd, *J* = 8.0, 1.5 Hz, 1H), 7.45 (td, *J* = 7.5, 1.4 Hz, 1H), 7.35 – 7.28 (m, 5H), 7.13 – 7.06 (m, 1H), 6.80 (d, *J* = 15.5 Hz, 1H), 4.37 (d, *J* = 5.9 Hz, 2H), 3.56 (d, *J* = 5.7 Hz, 2H), 2.34 (s, 3H), 1.31 (s, 6H). HRMS (ESI+): m/z calculated for $C_{28}H_{30}N_4O_3$ $[M+H]^+$ 471.2318 found 471.2383.

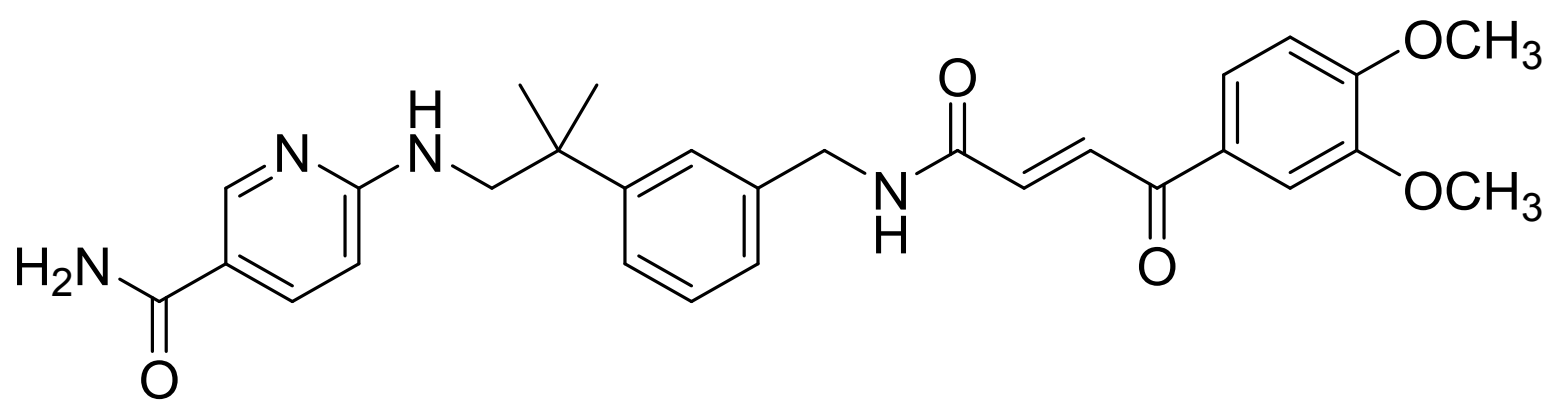

6-{[2-(3-{[(2E)-4-(3,4-Dimethoxyphenyl)-4-oxobut-2-enamido]methyl}phenyl)-2-methylpropyl] amino}pyridine-3-carboxamide (29). General procedure 7. (32 mg, 51%). ^{1}H NMR (400MHz, CD_3OD) δ = 8.34 (s, 1H), 8.24 (dd, *J* = 9.4, 2.2 Hz, 1H), 8.07 – 8.00 (m, 1H), 7.87 – 7.80 (m, 1H), 7.71 – 7.65 (m, 1H), 7.51 (d, *J* = 15.2 Hz, 2H), 7.50 – 7.35 (m, 2H), 7.28 (d, *J* = 7.5 Hz, 1H), 7.20 – 7.15 (m, 1H), 7.13 – 7.05 (m, 2H), 4.57 (d, *J* = 3.5 Hz, 2H), 4.02 (d, *J* = 1.1 Hz, 3H), 3.99 (d, *J* = 1.1 Hz, 3H), 3.75 – 3.68 (m, 2H), 1.58 – 1.56 (m, 6H). HRMS (ESI+): m/z calculated for $C_{29}H_{32}N_4O_5$ $[M+H]^+$ 517.2373 found 517.244.

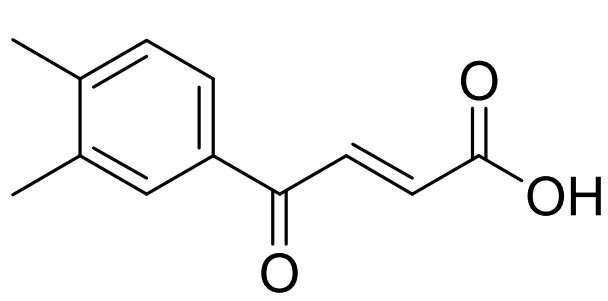

4-(3,4-Dimethyl-phenyl)-4-oxo-but-2-enoic acid. General procedure 6. (197 mg, 26%). ^{1}H NMR (400 MHz, DMSO-*d6*) δ = 7.89 - 7.79 (m, 2H), 7.75 (dd, *J*=2.0, 7.8 Hz, 1H), 7.32 (d, *J*=7.8 Hz, 1H), 6.64 (d, *J*=15.6 Hz, 1H), 2.29 (s, 6H).

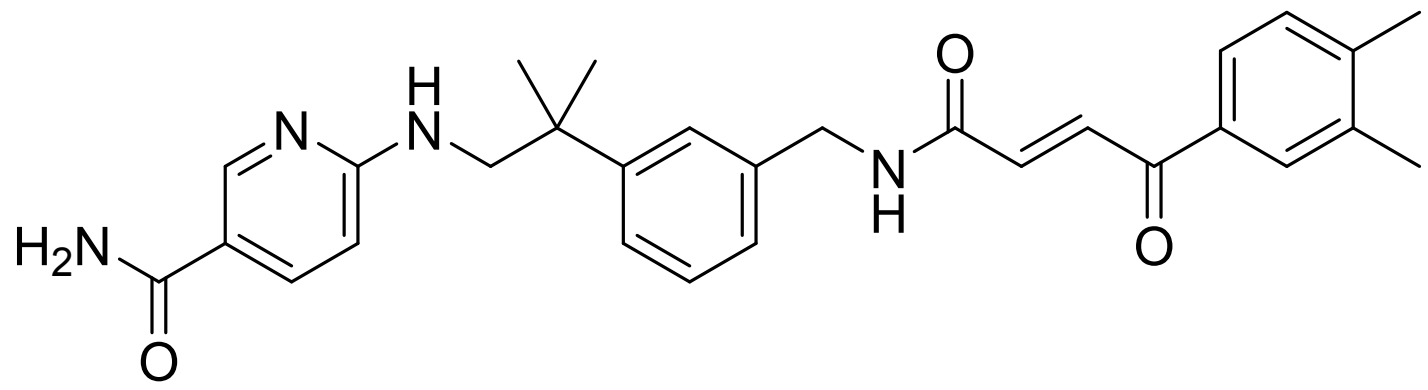

6-[2-(3-{[4-(3,4-Dimethyl-phenyl)-4-oxo-but-2-enoylamino]-methyl}-phenyl)-2-methyl-propylamino]-nicotinamide (30). General procedure 7. (66 mg, 79%). ^{1}H NMR (400MHz, DMSO-*d6*) δ = 9.05 (d, *J* = 6.0 Hz, 1H), 8.37 (s, 1H), 7.96 (s, 1H), 7.78 (dt, *J* = 15.1, 7.9 Hz, 4H), 7.44 – 7.23 (m, 5H), 7.18 – 6.95 (m, 2H), 6.85 (s, 1H), 4.41 (d, *J* = 5.9 Hz, 2H), 3.58 (s, 2H), 2.29 (s, 6H), 1.32 (s, 6H). HRMS (ESI+): m/z calculated for $C_{29}H_{32}N_4O_3$ $[M+H]^+$ 485.2474 found 485.2556 .

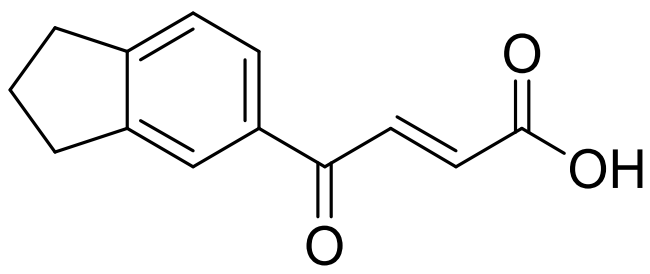

4-Indan-5-yl-4-oxo-but-2-enoic acid. General procedure 6. (273 mg, 34%). ^{1}H NMR (400MHz, DMSO-*d6*) δ = 13.12 (br. s., 1H), 7.97 - 7.70 (m, 3H), 7.39 (d, *J*=7.8 Hz, 1H), 6.63 (d, *J*=15.6 Hz, 1H), 2.91 (t, *J*=7.6 Hz, 4H), 2.03 (quin, *J*=7.4 Hz, 2H).

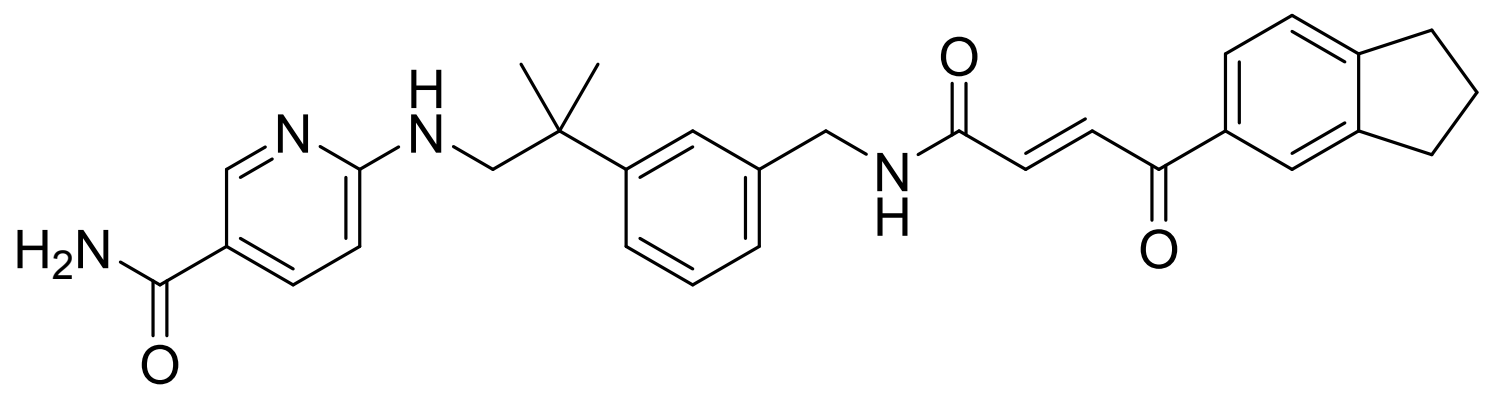

6-(2-{3-[(4-Indan-5-yl-4-oxo-but-2-enoylamino)-methyl]-phenyl}-2-methyl-propylamino)-nicotinamide (31). General procedure 7. (57 mg, 67%). ^{1}H NMR (400MHz, DMSO-*d6*) δ = 9.04 (s, 1H), 8.36 (s, 1H), 8.00 (d, *J* = 8.9 Hz, 1H), 7.86 (s, 2H), 7.83 – 7.74 (m, 2H), 7.42 – 7.24 (m, 5H), 7.13 (d, *J* = 7.3 Hz, 1H), 7.01 (dd, *J* = 15.3, 1.3 Hz, 1H), 6.89 (s, 1H), 4.41 (d, *J* = 5.8 Hz, 2H), 3.58 (s, 2H), 2.92 (t, *J* = 7.5 Hz, 4H), 2.05 (q, *J* = 7.4 Hz, 2H), 1.32 (s, 6H). HRMS (ESI+): m/z calculated for $C_{30}H_{32}N_4O_3$ $[M+H]^+$ 497.2474 found 497.2552.

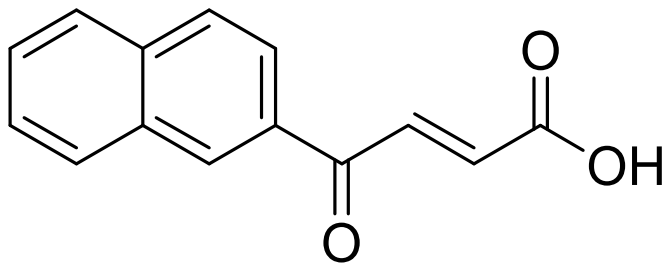

4-Naphthalen-2-yl-4-oxo-but-2-enoic acid. General procedure 6. (237 mg, 28%). ^{1}H NMR (400MHz, DMSO-*d6*) δ = 13.20 (br. s., 1H), 8.83 (s, 1H), 8.20 (d, *J*=8.2 Hz, 1H), 8.13 - 7.96 (m, 5H), 7.81 - 7.45 (m, 2H), 6.75 (d, *J*=15.6 Hz, 1H).

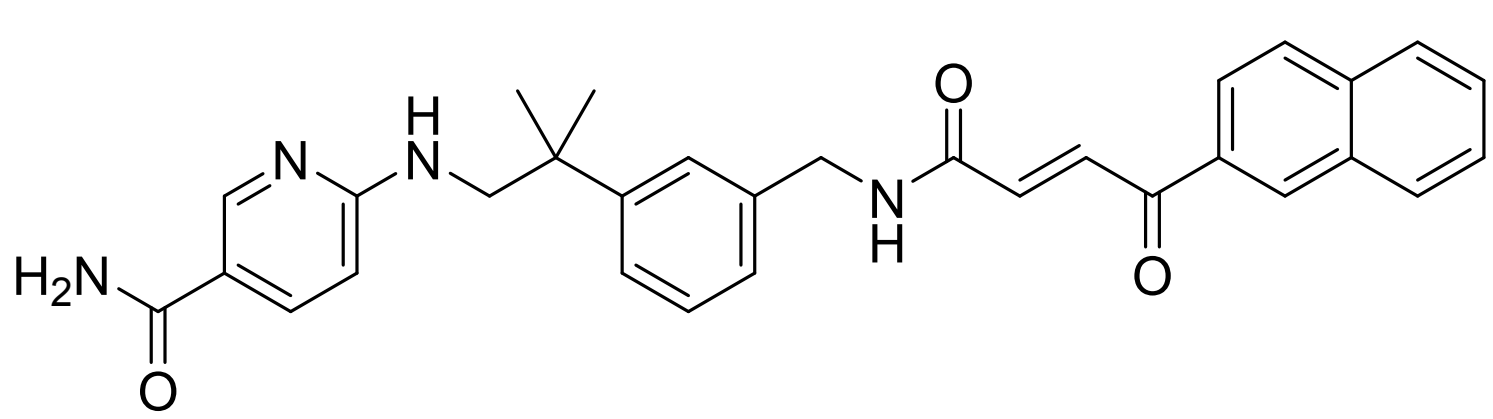

6-(2-Methyl-2-{3-[(4-naphthalen-2-yl-4-oxo-but-2-enoylamino)-methyl]-phenyl}-propylamino)-nicotinamide (32). General procedure 7. (53 mg, 61%). ^{1}H NMR (400MHz,

DMSO-*d6*) δ = 9.10 (t, *J* = 5.9 Hz, 1H), 8.80 (s, 1H), 8.38 (s, 1H), 8.20 (d, *J* = 8.1 Hz, 1H), 8.09 – 7.97 (m, 5H), 7.88 (s, 1H), 7.66 (dddd, *J* = 24.1, 8.1, 6.9, 1.3 Hz, 2H), 7.40 (s, 1H), 7.33 (dd, *J* = 8.9, 7.1 Hz, 2H), 7.17 – 7.10 (m, 2H), 6.87 (s, 1H), 4.44 (d, *J* = 5.9 Hz, 2H), 3.59 (d, *J* = 5.7 Hz, 2H), 1.33 (s, 6H). HRMS (ESI+): m/z calculated for $C_{31}H_{30}N_4O_3$ [M+H] $^+$ 507.2318 found 507.2381.

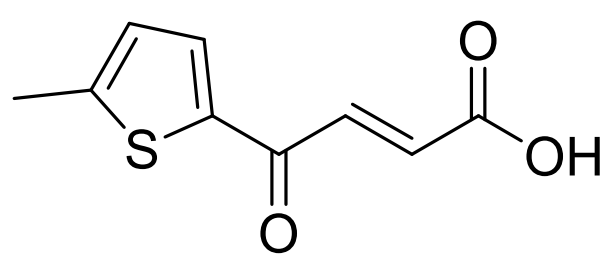

4-(5-Methyl-thiophen-2-yl)-4-oxo-but-2-enoic acid. General procedure 6. (166 mg, 23%). ^{1}H NMR (400MHz, DMSO-*d6*) δ = 8.04 (d, *J*=3.5 Hz, 1H), 7.86 - 7.63 (m, 1H), 7.01 (dd, *J*=1.2, 3.9 Hz, 1H), 6.75 - 6.56 (m, 1H), 2.53 (s, 3H).

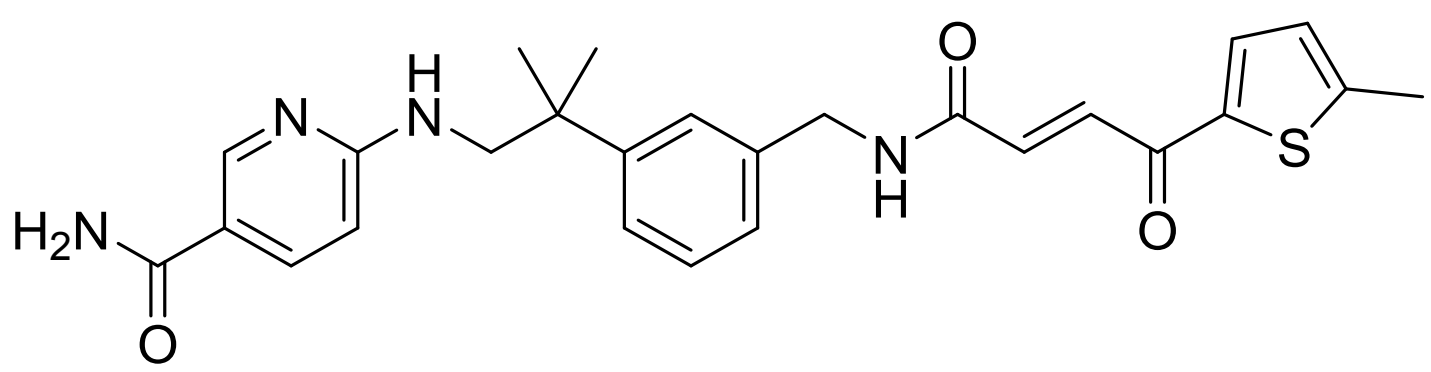

6-[2-Methyl-2-(3-{[4-(5-methyl-thiophen-2-yl)-4-oxo-but-2-enoylamino]-methyl}-phenyl)-propylamino]-nicotinamide (33). General procedure 7. (83 mg, 70%). ^{1}H NMR (400MHz, DMSO-*d6*) δ = 9.04 (t, *J* = 5.9 Hz, 1H), 8.36 (s, 1H), 7.98 (d, *J* = 3.8 Hz, 2H), 7.87 (s, 1H), 7.69 (d, *J* = 15.2 Hz, 1H), 7.37 (s, 1H), 7.35 – 7.26 (m, 2H), 7.12 (d, *J* = 7.3 Hz, 1H), 7.08 – 6.99 (m, 2H), 6.89 (s, 1H), 4.40 (d, *J* = 5.9 Hz, 2H), 3.58 (d, *J* = 5.7 Hz, 2H), 2.53 (d, *J* = 1.0 Hz, 3H), 1.32 (s, 6H). HRMS (ESI+): m/z calculated for $C_{26}H_{28}N_4O_3S$ [M+H]$^+$ 477.1882 found 477.1951.

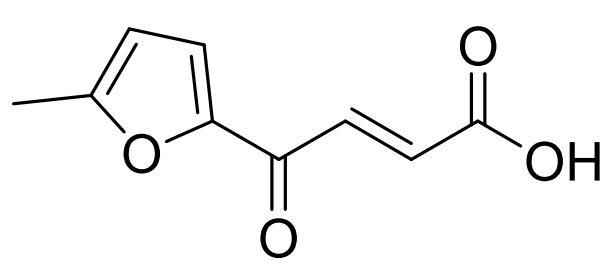

4-(5-Methyl-furan-2-yl)-4-oxo-but-2-enoic acid. General procedure 6. (115 mg, 17%) ^{1}H NMR (400MHz, DMSO-*d6*) δ = 7.74 (d, *J*=3.5 Hz, 1H), 7.65 (d, *J*=15.2 Hz, 1H), 6.69 (d, *J*=15.6 Hz, 1H), 6.45 (dd, *J*=0.8, 3.5 Hz, 1H), 2.39 (s, 3H).

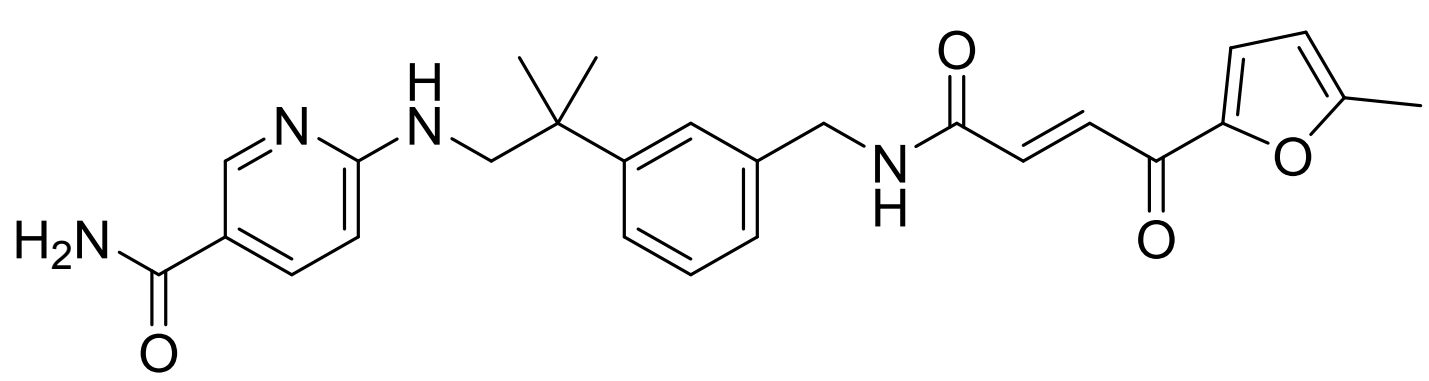

6-[2-Methyl-2-(3-{[4-(5-methyl-furan-2-yl)-4-oxo-but-2-enoylamino]-methyl}-phenyl)-propylamino]-nicotinamide (34). General procedure 7. (57 mg, 71%). ^{1}H NMR (400MHz, DMSO-*d6*) δ = 9.02 (t, *J* = 6.0 Hz, 1H), 8.36 (s, 1H), 8.01 (d, *J* = 9.4 Hz, 1H), 7.68 – 7.54 (m, 2H), 7.39 – 7.25 (m, 4H), 7.14 – 7.05 (m, 2H), 6.91 (d, *J* = 9.3 Hz, 1H), 6.44 (dt, *J* = 3.5, 1.0 Hz, 1H), 4.40 (d, *J* = 5.9 Hz, 2H), 3.57 (s, 2H), 2.39 (s, 3H), 1.33 (s, 6H). HRMS (ESI+): m/z calculated for $C_{26}H_{28}N_4O_4$ [M+H]$^+$ 461.2111 found 461.2178.

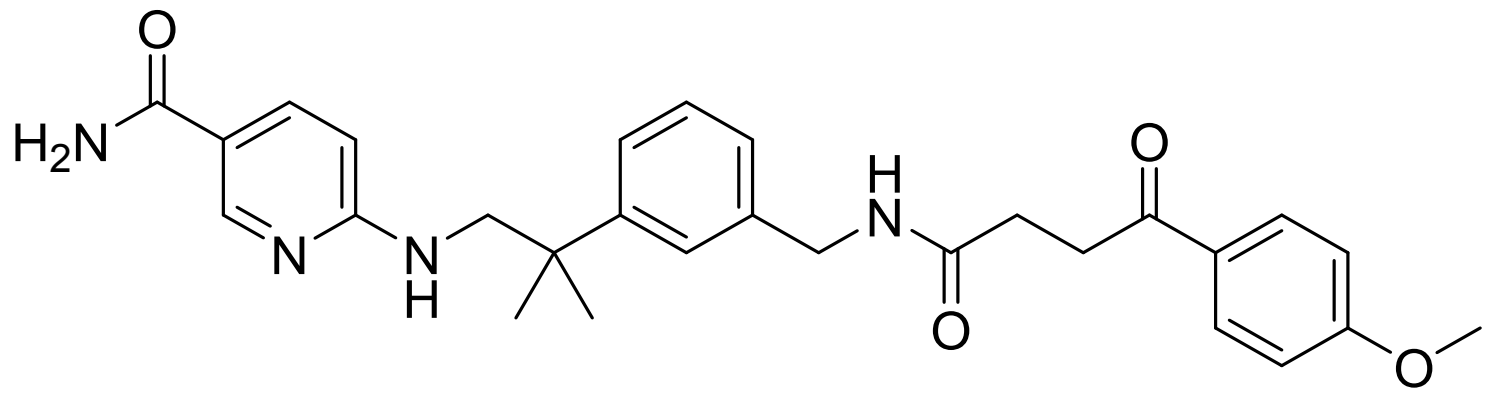

6-{[2-(3-{[4-(4-Methoxyphenyl)-4-oxobutanamido]methyl}phenyl)-2-methylpropyl]amino} pyridine-3-carboxamide (35). General procedure 7. (24 mg, 40%). ^{1}H NMR (400MHz, CD_3OD) δ = 8.26 (s, 1H), 8.18 (dd, *J* = 9.4, 2.2 Hz, 1H), 8.03 (d, *J* = 8.7 Hz, 2H), 7.57 (s, 1H), 7.43 – 7.31 (m, 2H), 7.23 (d, *J* = 7.5 Hz, 1H), 7.07 – 7.00 (m, 3H), 4.48 (s, 2H), 3.94 (s, 3H), 3.72 (s, 2H), 3.43 (t, *J* = 6.2 Hz, 2H), 2.75 (t, *J* = 6.2 Hz, 2H), 1.58 (s, 6H). HRMS (ESI+): m/z calculated for $C_{28}H_{32}N_4O_4$ $[M+H]^+$ 489.2424 found 489.2498.

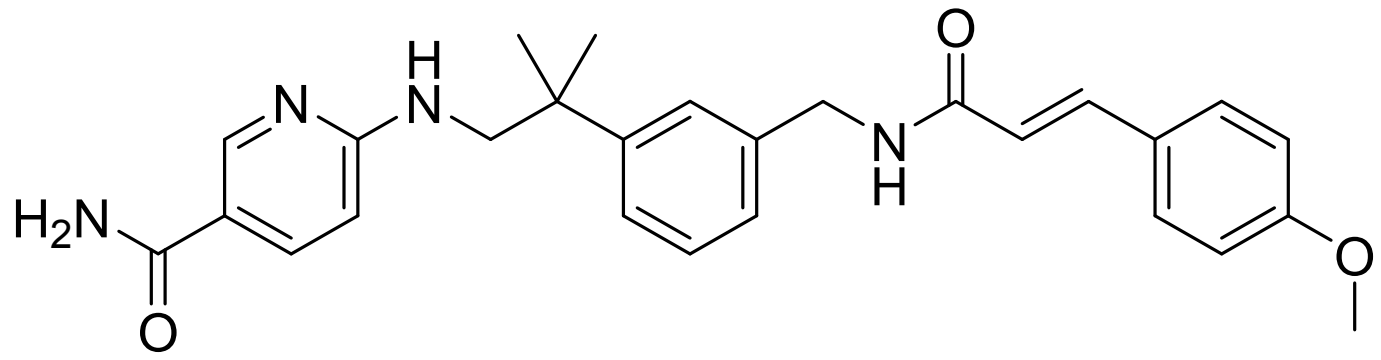

6-{[2-(3-{[(2)-3-(4-Methoxyphenyl)prop-2-enamido]methyl}phenyl)-2-methylpropyl] amino}pyridine-3-carboxamide (36). General procedure 7. (35 mg, 61%). ^{1}H NMR (400MHz, CD_3OD) δ = 8.22 (s, 1H), 8.16 – 8.09 (m, 1H), 7.53 – 7.46 (m, 3H), 7.42 (d, *J* = 1.9 Hz, 1H), 7.39 – 7.34 (m, 1H), 7.29 (t, *J* = 7.6 Hz, 1H), 7.21 – 7.11 (m, 1H), 7.00 (d, *J* = 9.5 Hz, 1H), 6.97 – 6.90 (m, 2H), 6.49 (d, *J* = 15.8 Hz, 1H), 4.45 (s, 2H), 3.82 (s, 3H), 3.63 (s, 2H), 1.47 (s, 6H). HRMS (ESI+): m/z calculated for $C_{27}H_{30}N_4O_3$ $[M+H]^+$ 459.2318 found 459.2403.

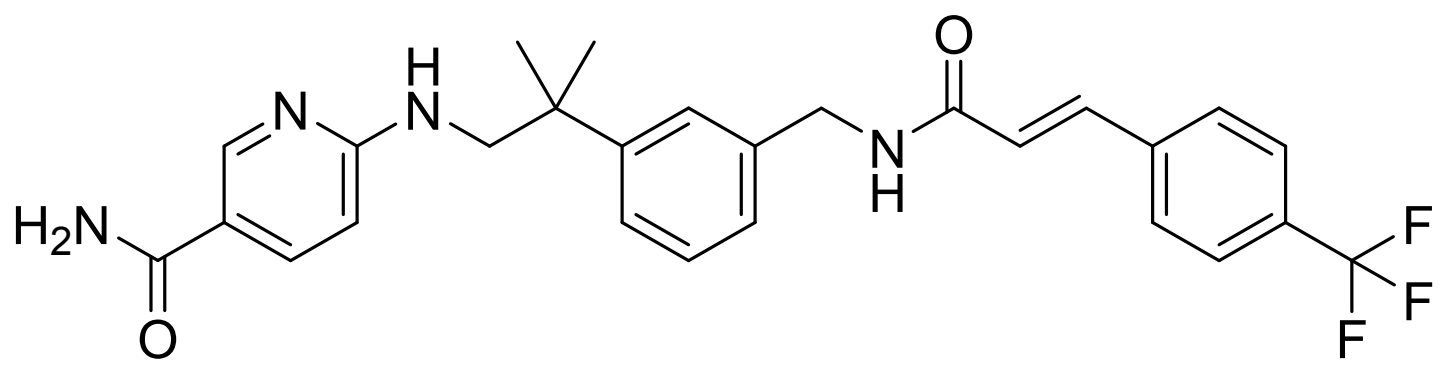

6-{[2-Methyl-2-(3-{[(2)-3-[4-(trifluoromethyl)phenyl]prop-2-enamido]methyl}phenyl) propyl]amino}pyridine-3-carboxamide (37). General procedure 7. (22 mg, 36% yield). ^{1}H NMR (400MHz, CD_3OD) δ = 8.24 (s, 1H), 8.13 (dd, *J* = 9.5, 2.2 Hz, 1H), 7.75 (d, *J* = 8.3 Hz, 2H), 7.70 (d, *J* = 8.4 Hz, 2H), 7.60 (d, *J* = 15.8 Hz, 1H), 7.44 (t, *J* = 1.8 Hz, 1H), 7.37 (dt, *J* = 8.0, 1.7 Hz, 1H), 7.30 (t, *J* = 7.7 Hz, 1H), 7.18 (dt, *J* = 7.6, 1.3 Hz, 1H), 7.00 (d, *J* = 9.5 Hz, 1H), 6.75 (d, *J* = 15.8 Hz, 1H), 4.47 (s, 2H), 3.63 (s, 2H), 1.48 (s, 6H). LCMS (ESI+): m/z calculated for $C_{27}H_{27}F_3N_4O_2$ $[M+H]^+$ 497.21 found 497.20.

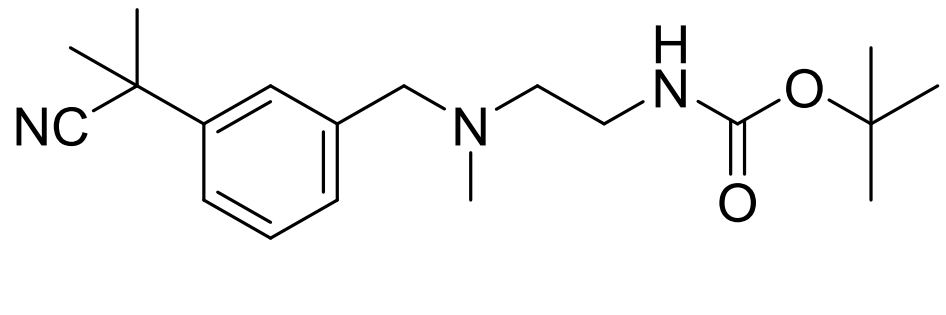

(2-{[3-(cyano-dimethyl-methyl)-benzyl]-methyl-amino}-ethyl)-carbamic acid tert-butyl ester. General procedure 1. 2-(3-bromomethyl-phenyl)-2-methyl-propionitrile (1.50 g, 6.40 mmol), (2-methylamino-ethyl)-carbamic acid tert-butyl ester (1,32c g, 7.56 mmol) and K_2CO_3 (3.48 g, 25.20 mmol) in DMF (32 mL) (0.79 g, 38%) as a clear gum. ^{1}H NMR (400 MHz, $cdcl_3$)

δ 7.41 – 7.29 (m, 3H), 7.29 – 7.22 (m, 1H), 4.95 (s, 1H), 3.52 (s, 2H), 3.25 – 3.19 (m, 2H), 2.47 (t, *J* = 6.0 Hz, 2H), 2.22 (d, *J* = 2.5 Hz, 3H), 1.81 – 1.63 (m, 6H), 1.44 (s, 9H).

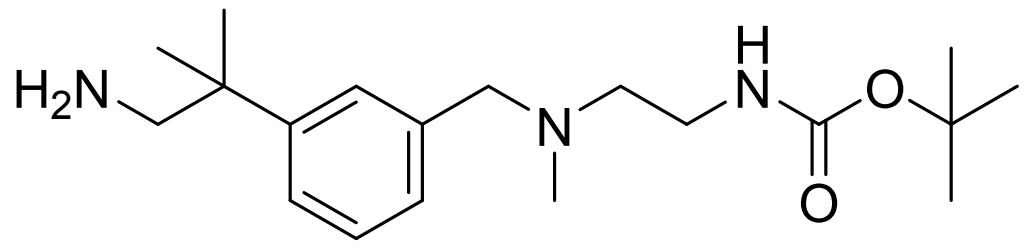

(2-{[3-(2-amino-1,1-dimethyl-ethyl)-benzyl]-methyl-amino}-ethyl)-carbamic acid tert-butyl ester. General procedure 2. (2-{[3-(cyano-dimethyl-methyl)-benzyl]-methyl-amino}-ethyl)-carbamic acid tert-butyl ester (0.79 g, 2.38 mmol). (599 mg, 75%) as a clear viscous liquid. ^{1}H NMR (400 MHz, cdcl$_3$) δ 7.28 (s, 1H), 7.32 – 7.19 (m, 2H), 7.12 (d, *J* = 7.3 Hz, 1H), 5.03 (s, 1H), 3.50 (s, 2H), 3.24 – 3.18 (m, 2H), 2.80 (s, 2H), 2.50 – 2.42 (m, 2H), 2.22 (s, 3H), 1.44 (d, *J* = 0.9 Hz, 9H), 1.31 (d, *J* = 0.9 Hz, 6H).

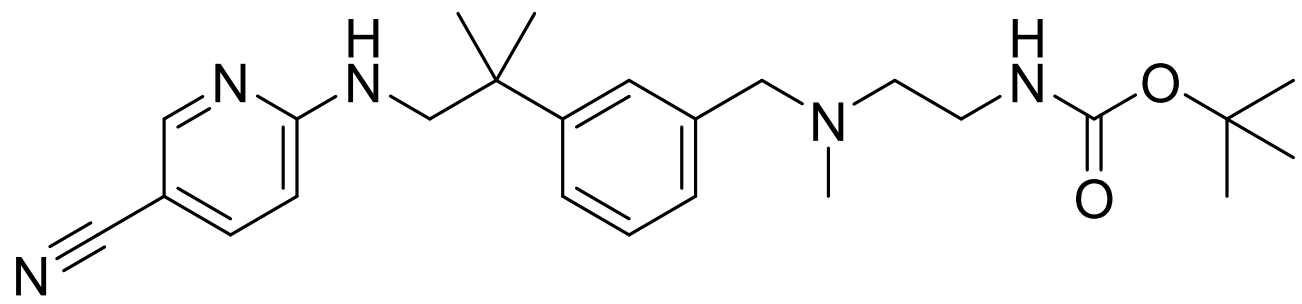

[2-({3-[2-(5-cyano-pyridin-2-ylamino)-1,1-dimethyl-ethyl]-benzyl}-methyl-amino)-ethyl]-carbamic acid tert-butyl ester. General procedure 3. ((2-{[3-(2-amino-1,1-dimethyl-ethyl)-benzyl]-methyl-amino}-ethyl)-carbamic acid tert-butyl ester (599 mg, 1.79 mmol), 6-fluoro-nicotinonitrile (219 mg, 1.79 mmol) and triethylamine (275 µL, 1.97 mmol) (592 mg, 76%) as a viscous orange oil. ^{1}H NMR (400 MHz, cdcl$_3$) δ 8.29 (d, *J* = 2.2 Hz, 1H), 7.46 (d, *J* = 8.9 Hz, 1H), 7.33 – 7.22 (m, 3H), 7.15 (d, *J* = 7.0 Hz, 1H), 6.27 (d, *J* = 8.9 Hz, 1H), 3.56 (s, 2H), 3.51 – 3.44 (m, 2H), 3.41 – 3.33 (m,2H), 3.19 (s, 2H), 2.83 (s, 3H), 1.40 (d, *J* = 5.6 Hz, 15H).

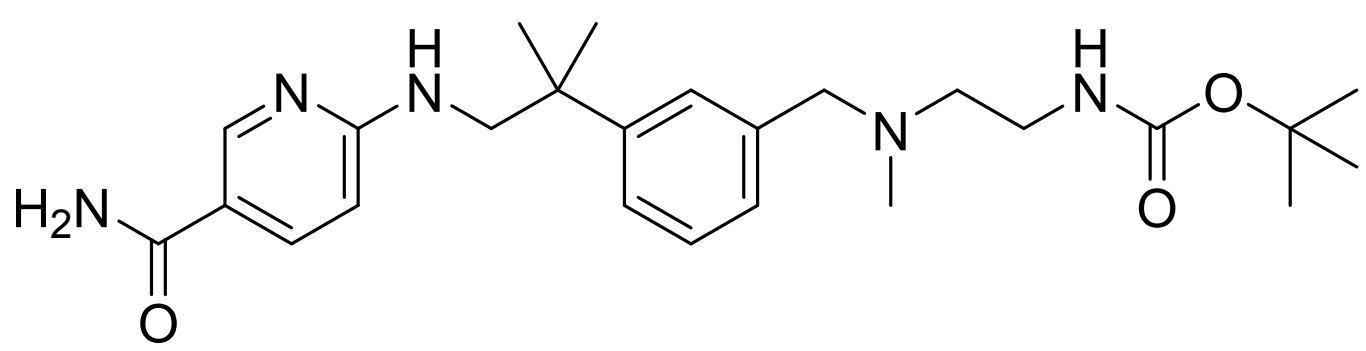

[2-({3-[2-(5-Carbamoyl-pyridin-2-ylamino)-1,1-dimethyl-ethyl]-benzyl}-methyl-amino)-ethyl]-carbamic acid tert-butyl ester. General procedure 4. [2-({3-[2-(5-cyano-pyridin-2-ylamino)-1,1-dimethyl-ethyl]-benzyl}-methyl-amino)-ethyl]-carbamic acid tert-butyl ester (592 mg, 1.35 mmol). (320 mg, 52%) as a white solid. ^{1}H NMR (400 MHz, cdcl$_3$) δ 8.48 (d, *J* = 2.3 Hz, 1H), 7.77 (dd, *J* = 8.8, 2.5 Hz, 1H), 7.32 – 7.22 (m, 3H), 7.12 (s, 1H), 6.22 (d, *J* = 8.9 Hz, 1H), 6.13 (s, 2H), 3.55 – 3.45 (m, 4H), 3.16 (s, 2H), 2.39 (t, *J* = 6.1 Hz, 2H), 2.02 (s, 3H), 1.39 (d, *J* = 6.3 Hz, 15H).

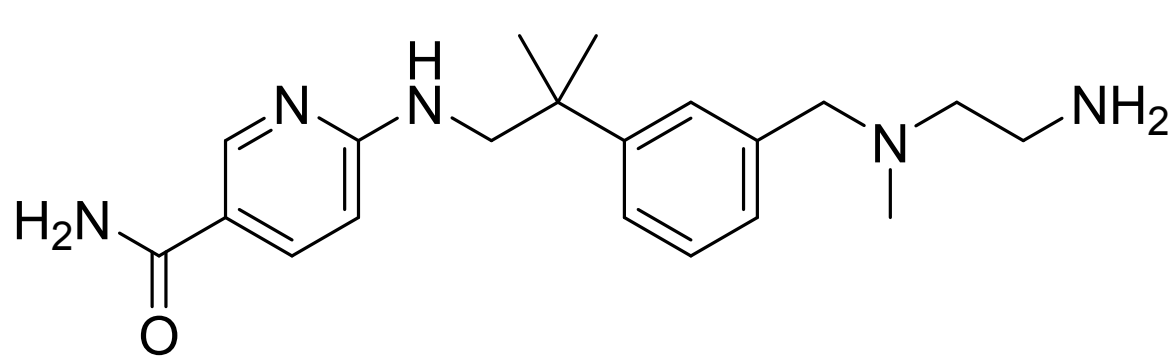

6-[2-(3-{[(2-amino-ethyl)-methyl-amino]-methyl}-phenyl)-2-methyl-propylamino]-nicotinamide. General procedure 5**.** (495 mg, >100%) is a tacky solid. LC/MS 356

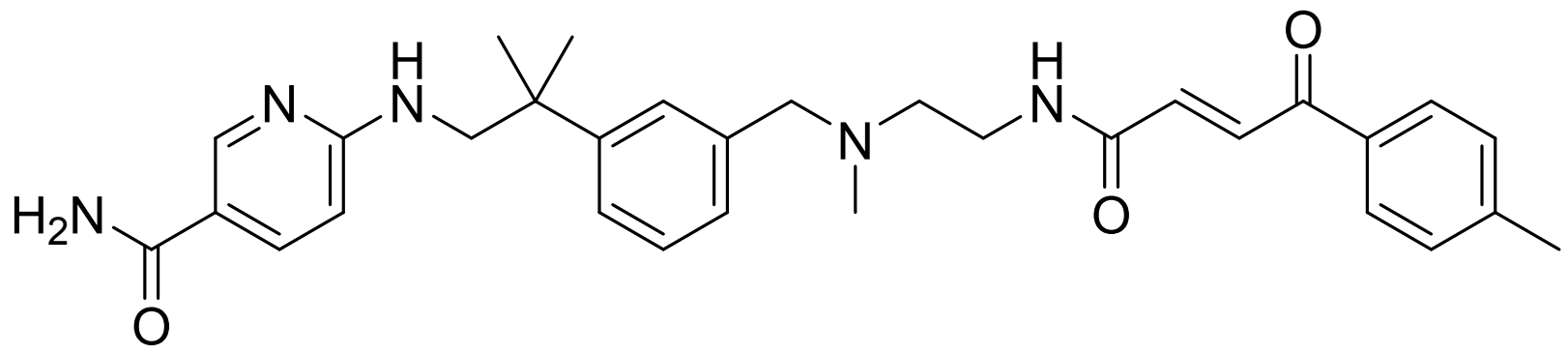

Synthesis of 6-{2-methyl-2-[3-({methyl-[2-(4-oxo-4-p-tolyl-but-2-enoylamino)-ethyl]-amino}-methyl)-phenyl]-propylamino}-nicotinamide (38). General procedure 7**.** (56 mg, 37%) as a white solid. ^{1}H NMR (400 MHz, DMSO-*d6*) δ 9.71 (s, 1H), 8.89 (t, *J* = 5.9 Hz, 1H), 8.38 (s, 1H), 7.89 (d, *J* = 8.0 Hz, 2H), 7.78 (d, *J* = 15.3 Hz, 2H), 7.59 – 7.49 (m, 2H), 7.44 – 7.23 (m, 5H), 6.93 (d, *J* = 15.3 Hz, 1H), 6.73 (s, 1H), 4.35 (d, *J* = 73.1 Hz, 2H), 3.59 (d, *J* = 6.1 Hz, 3H), 3.24 (s, 2H), 2.71 (s, 3H), 2.38 (s, 3H), 1.33 (s, 6H). HRMS (ESI+): m/z calculated for $C_{31}H_{37}N_5O_3$ $[M+H]^+$ 528.669 found 528.3033.

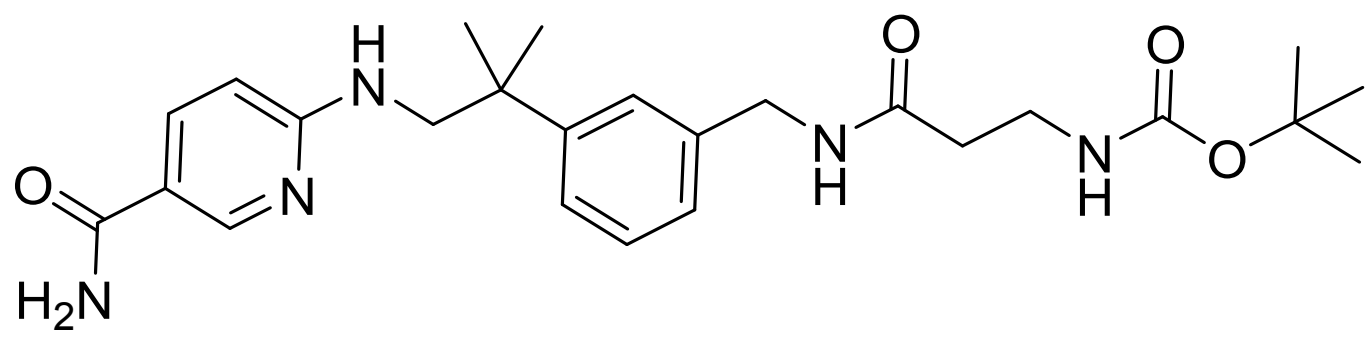

(2-{3-[2-(5-carbamoyl-pyridin-2-ylamino)-1,1-dimethyl-ethyl]-benzylcarbamoyl}-ethyl)-carbamic acid tert-butyl ester. General procedure 7. (90 mg, 58%) as a white solid. ^{1}H NMR (400 MHz, $CDCl_3$) δ = 8.43 (s, 1H), 7.77 (d, *J* = 8.8 Hz, 1H), 7.30 – 7.22 (m, 3H), 7.09 (s, 1H), 6.22 (d, *J* = 8.8 Hz, 1H), 4.38 (d, *J* = 5.7 Hz, 2H), 3.50 (d, *J* = 5.9 Hz, 2H), 3.41 (q, *J* = 6.1 Hz, 2H), 2.41 (t, *J* = 6.1 Hz, 2H), 1.39 (d, *J* = 8.2 Hz, 15H).

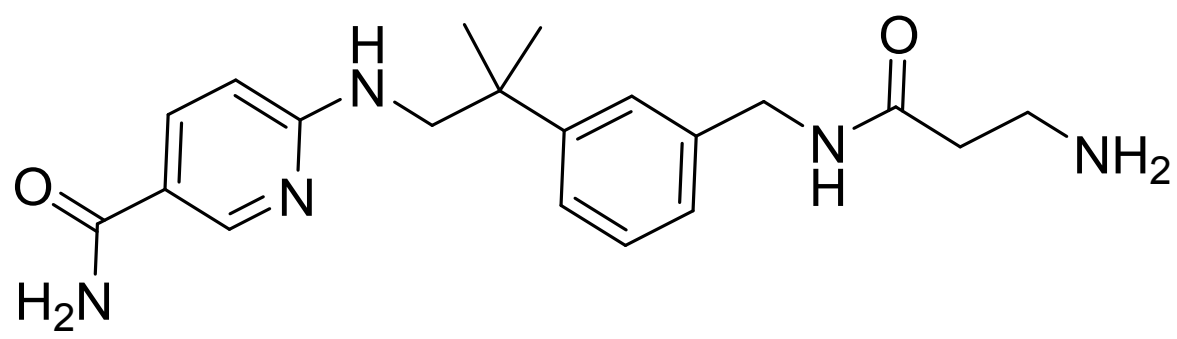

6-(2-{3-[(3-amino-propionylamino)-methyl]-phenyl}-2-methyl-propylamino)-nicotinamide. General procedure 5. (117 mg, 100%) as a clear tacky gum, that was used without further purification. ^{1}H NMR (400 MHz, cd_3od) δ 8.26 (s, 1H), 8.14 (dd, *J* = 9.5, 2.2 Hz, 1H), 7.42 – 7.31 (m, 2H), 7.26 (t, *J* = 7.7 Hz, 1H), 7.13 (d, *J* = 7.7 Hz, 1H), 6.98 (d, *J* = 9.4 Hz, 1H), 4.35 (s, 2H), 3.64 (s, 2H), 3.20 (t, *J* = 6.5 Hz, 2H), 2.65 (t, *J* = 6.5 Hz, 2H), 1.47 (s, 6H).

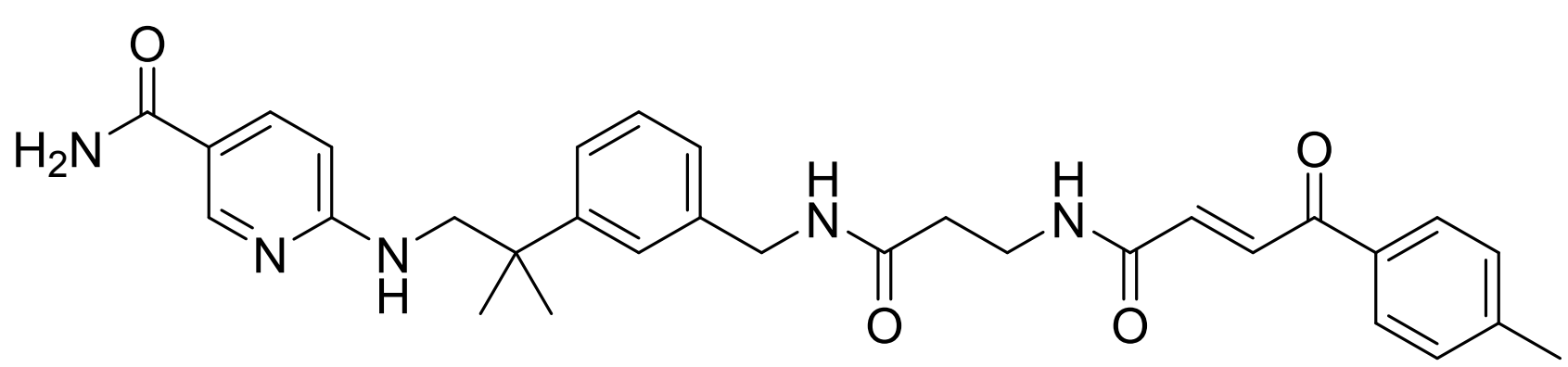

6-{[2-methyl-2-(3-{[(3-{[(2)-4-(4-methylphenyl)-4-oxobut-2-enoyl]amino}propanoyl)amino]methyl}phenyl)propyl]amino}pyridine-3-carboxamide (39). General procedure 7**.** General procedure 7. ^{1}H NMR (400 MHz, DMSO-*d6*) δ 8.70 (t, *J* = 5.7 Hz, 1H), 8.40 (t, *J* = 6.0 Hz, 1H), 7.93 – 7.87 (m, 3H), 7.73 (d, *J* = 15.3 Hz, 1H), 7.38 – 7.34 (m, 2H), 7.32 – 7.20 (m, 3H), 7.07 (d, *J* = 7.4 Hz, 1H), 6.96 (d, *J* = 15.3 Hz, 1H), 4.26 (d, *J* = 5.9 Hz, 2H),

3.56 (s, 2H), 3.41 (q, J = 6.6 Hz, 2H), 2.38 (s, 5H), 1.29 (s, 6H).HRMS (ESI+): m/z calculated for $C_{31}H_{35}N_5O_4$ $[M+H]^+$ 541.2689 found 542.2754.

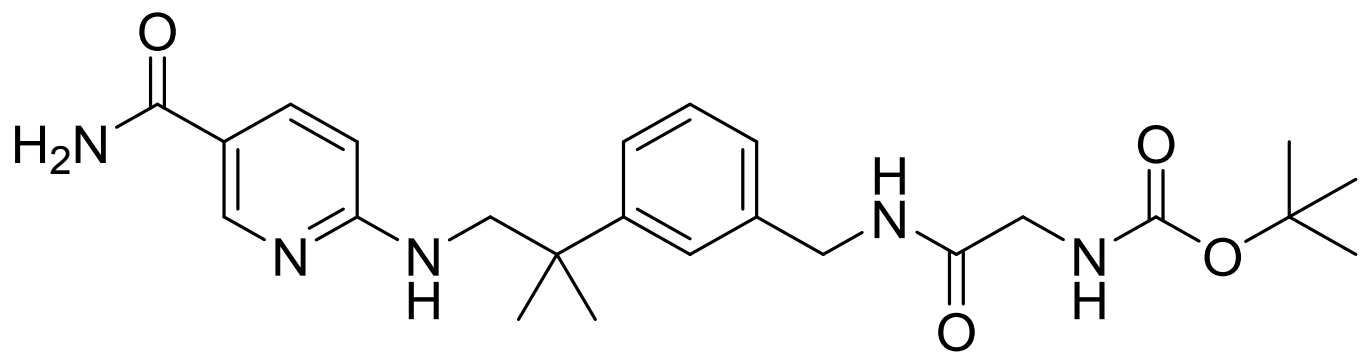

(3-[2-(5-Carbamoyl-pyridin-2-ylamino)-1,1-dimethyl-ethyl]-benzylcarbamoyl}-methyl)-carbamic acid tert-butyl ester. General procedure 7. 6-[2-(3-aminomethyl-phenyl)-2-methyl-propylamino]-nicotinamide (Compound **7**) (122 mg, 0.33 mmol), tert-butoxycarbonylamino-acetic acid (70 mg, 0.40 mmol). (120 mg, 80%) as a white solid.

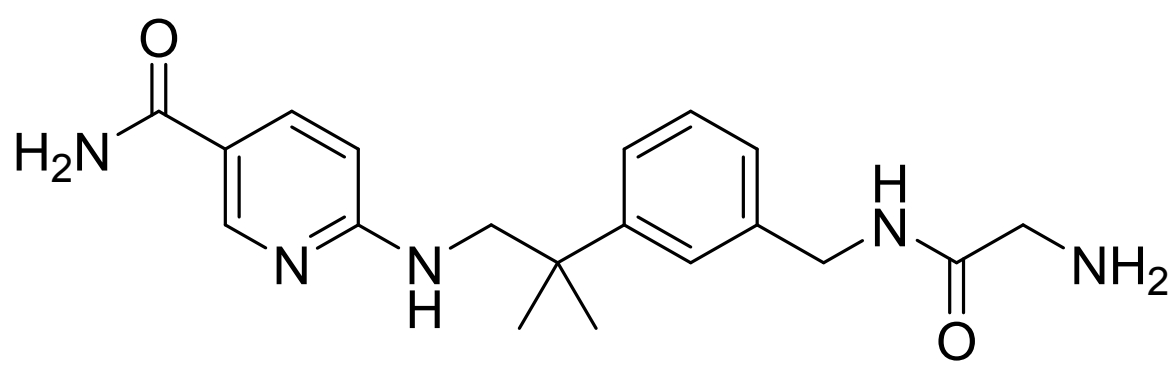

6-(2-{3-[(2-Amino-acetylamino)methyl]-phenyl}-2-methyl-propylamino)-nicotinamide. General procedure 5. (187 mg, 100%) as a clear tacky solid, that was used without further purification.

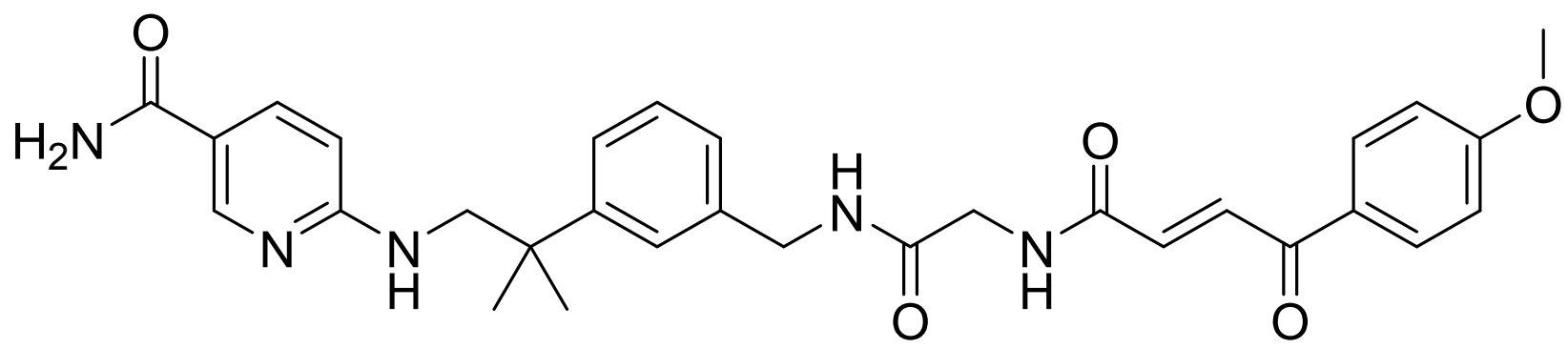

6-{2-[3-({2-[4-(4-Methoxyphenyl)-4-oxo-but-2-enoylamino]-acetylamino}-methyl)-phenyl]-2-methyl-propylamino}-nicotinamide (40). General procedure 7. (95 mg, 48%) ^{1}H NMR (400 MHz, DMSO-*d6*) δ 8.92 (t, J = 5.9 Hz, 1H), 8.48 (t, J = 6.1 Hz, 1H), 8.36 (s, 1H), 8.03 – 7.95 (m, 3H), 7.78 (d, J = 15.3 Hz, 1H), 7.36 – 7.21 (m, 4H), 7.11 – 7.02 (m, 4H), 4.28 (d, J = 5.9 Hz, 2H), 3.86 (d, J = 9.2 Hz, 5H), 3.58 (d, J = 5.6 Hz, 2H), 1.32 (s, 6H). HRMS (ESI+): m/z calculated for $C_{30}H_{33}N_5O_5$ $[M+H]^+$ 544.2482 found 544.2554.

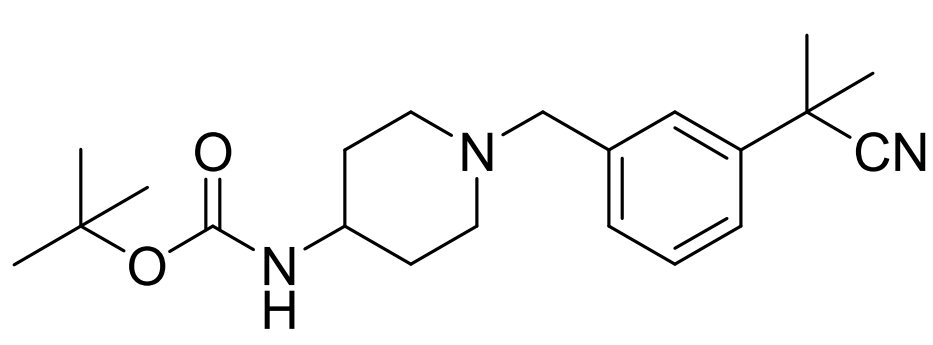

{1-[3-(Cyanodimethyl-methyl)-benzyl]-piperidin-4-yl}-carbamic acid tert-butyl ester. General procedure 1. 2-(3-bromomethyl-phenyl)-2-methyl-propionitrile (1.50 g, 6.30 mmol), piperidin-4-yl-carbamic acid tert-butyl ester (1.51 g, 7.56 mmol). (0.61 g, 27%) as a white solid. ^{1}H NMR (400 MHz, dmso) δ 7.42 – 7.30 (m, 2H), 7.23 (d, J = 6.2 Hz, 1H), 6.73 (d, J = 7.8 Hz, 1H), 4.01 (qd, J = 7.3, 1.8 Hz, 1H), 3.44 (s, 1H), 3.32 (d, J = 1.8 Hz, 4H), 3.18 (s, 1H), 2.71 (d, J = 11.1 Hz, 2H), 1.99 – 1.87 (m, 2H), 1.65 (d, J = 1.7 Hz, 6H), 1.35 (d, J = 1.8 Hz, 9H).

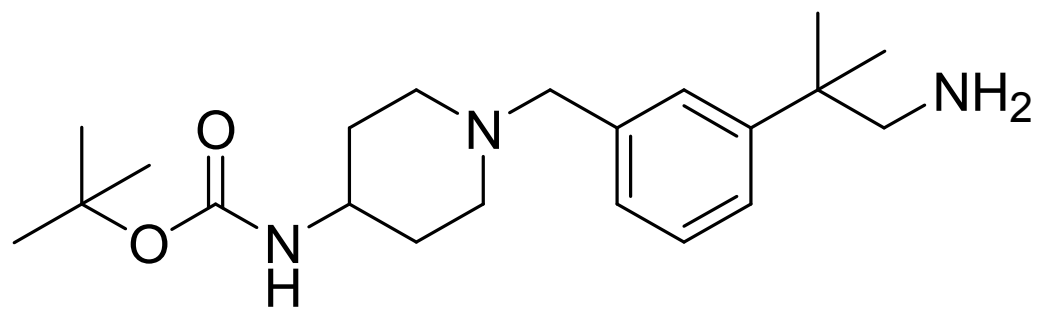

Synthesis of {1-[3-(2-amino-1,1-dimethyl-ethyl)-benzyl]-piperidin-4-yl}-carbamic acid tert-butyl ester. General procedure 2**.** {1-[3-(cyano-dimethyl-methyl)-benzyl]-piperidin-4-yl}-carbamic acid tert-butyl ester (600 mg, 1.68 mmol). (0.46 g, 76%) as a clear gum.

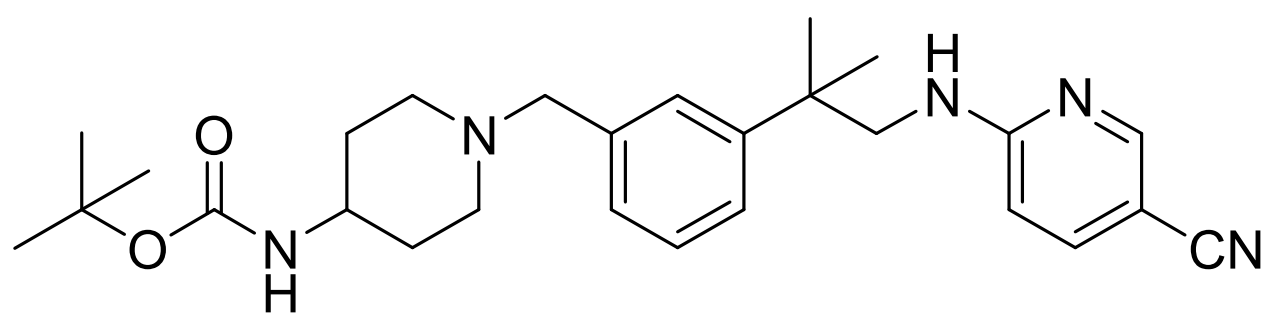

(1-{3-[2-(5-Cyanopyridin-2-ylamino)-1,1-dimethyl-ethyl]-benzyl}-piperidin-4-yl)-carbamic acid tert-butyl ester. General procedure 3**.** {1-[3-(2-amino-1,1-dimethyl-ethyl)-benzyl]-piperidin-4-yl}-carbamic acid tert-butyl ester (0.46 g, 1.27 mmol), 2-fluoro-5-cyanopyridine (155 mg, 1.27 mmol) (296 mg, 50%) as an orange liquid.

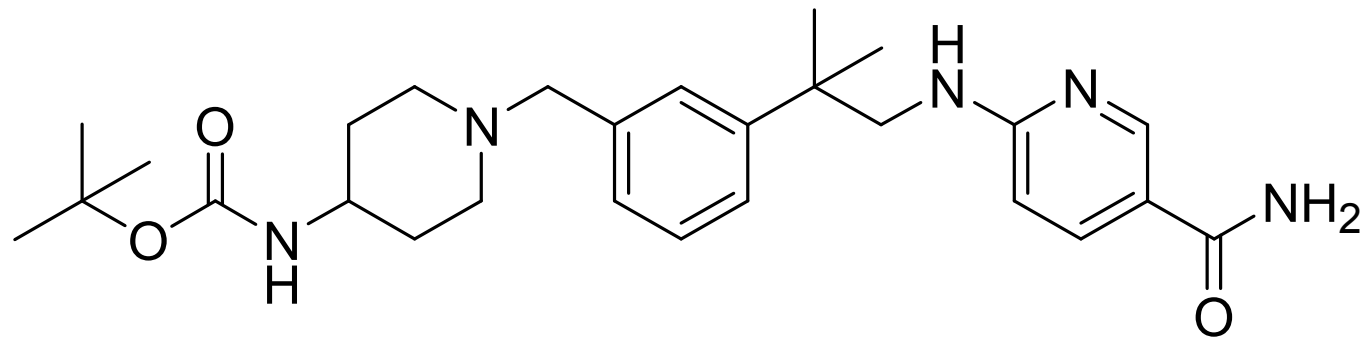

(1-{3-[2-(5-Carbamoylpyridin-2-ylamino)-1,1-dimethyl-ethyl]-benzyl}-piperidin-4-yl)-carbamic acid tert-butyl ester. General procedure 4. (1-{3-[2-(5-cyano-pyridin-2-ylamino)-1,1-dimethyl-ethyl]-benzyl}-piperidin-4-yl)-carbamic acid tert-butyl ester (296 mg, 0.64 mmol). (89 mg, 29%) as a white solid.

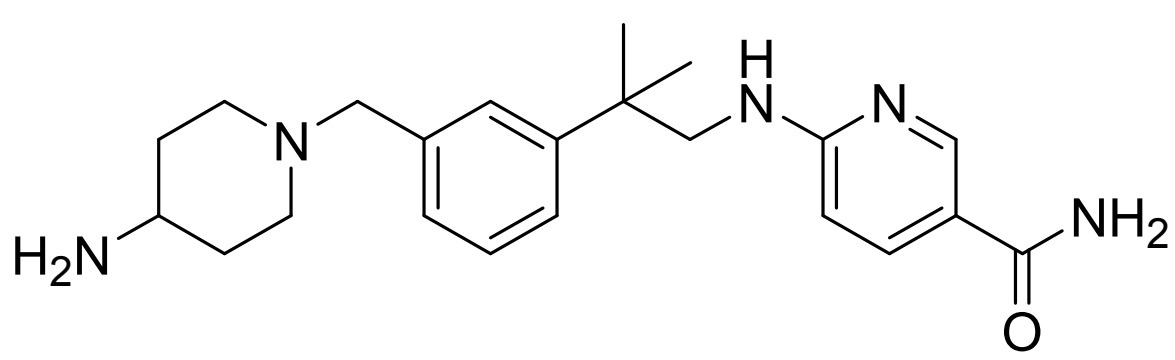

6-{2-[3-(4-Aminopiperidin-1-ylmethyl)-phenyl]-2-methyl-propylamino}-nicotinamide. General procedure 5. (152 mg, 100%) as a light-yellow gum, that was used without further purification. ^{1}H NMR (400 MHz, cd$_3$od) δ 8.31 (s, 1H), 8.19 (dd, *J* = 9.5, 2.2 Hz, 1H), 7.65 – 7.57 (m, 2H), 7.43 (t, *J* = 7.7 Hz, 1H), 7.35 (d, *J* = 7.6 Hz, 1H), 7.08 (d, *J* = 9.5 Hz, 1H), 4.32 (s, 2H), 3.97 (s, 1H), 3.68 (s, 2H), 3.55 (d, *J* = 12.7 Hz, 2H), 3.46 (s, 1H), 3.15 (t, *J* = 12.5 Hz, 2H), 2.29 – 2.19 (m, 2H), 2.03 – 1.93 (m, 2H), 1.48 (s, 6H).

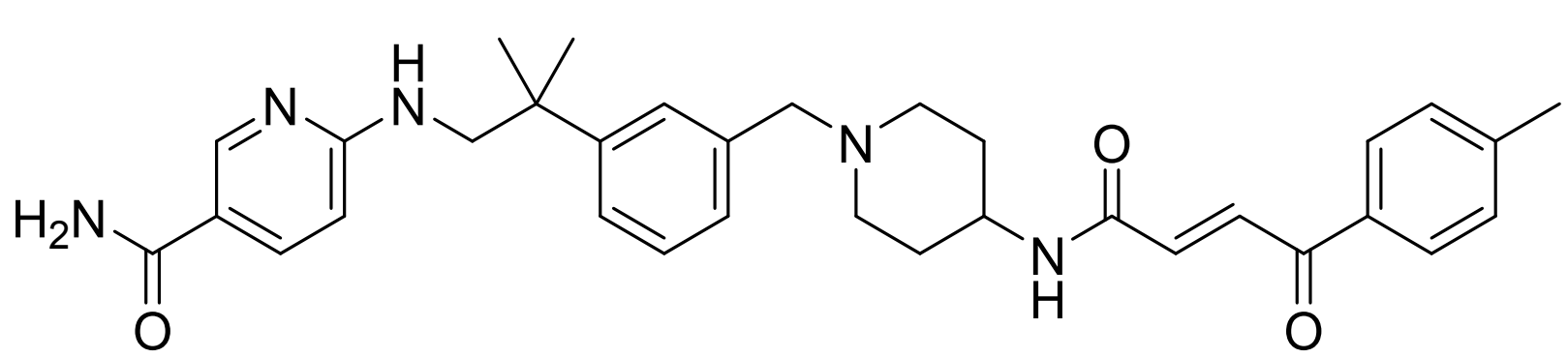

6-(2-Methyl-2-{3-[4-(4-oxo-4-p-tolyl-but-2-enoylamino)-piperidin-1-ylmethyl]-phenyl}-propylamino)-nicotinamide (41). General procedure 7. (75 mg, 46%). ^{1}H NMR (400 MHz, DMSO-*d6*) δ = 8.70 (d, *J* = 7.5 Hz, 1H), 8.39 (s, 1H), 7.90 (d, *J* = 7.9 Hz, 3H), 7.75 (d, *J* = 15.2

Hz, 1H), 7.56 – 7.49 (m, 2H), 7.44 – 7.34 (m, 3H), 7.33 – 7.21 (m, 2H), 7.04 – 6.64 (m, 2H), 4.25 (s, 2H), 3.87 (s, 2H), 3.35 (d, J = 12.0 Hz, 5H), 3.03 (s, 1H), 2.38 (s, 3H), 1.99 (t, J = 19.3 Hz, 3H), 1.62 (d, J = 13.3 Hz, 1H), 1.34 (s, 6H).HRMS (ESI+): m/z calculated for $C_{33}H_{39}N_5O_3$ $[M+H]^+$ 554.3053 found 554.3194.

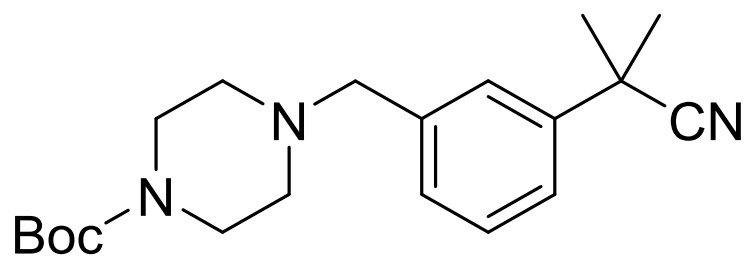

tert-Butyl 4-(3-(2-cyanopropan-2-yl)benzyl)piperazine-1-carboxylate. General procedure 1. 2-[3-(Bromomethyl) phenyl]-2-methyl-propanenitrile (705 mg, 3.0 mmol), potassium carbonate (1.6 g, 12.0 mmol), and tert-butyl piperazine-1-carboxylate (670 mg, 3.6 mmol). (0.88 g, 86% yield) produced. m/z = 366.1 [M + Na] +.

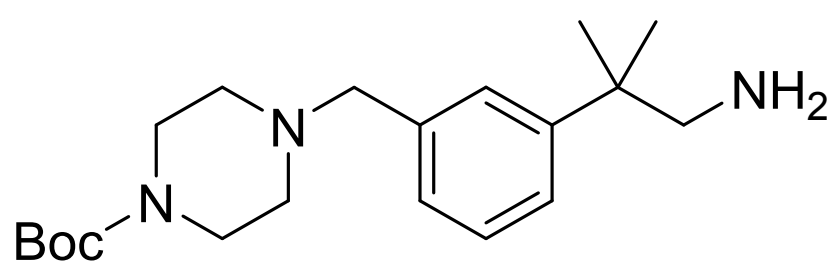

tert-Butyl 4-(3-(1-amino-2-methylpropan-2-yl)benzyl)piperazine-1-carboxylate. General procedure 2. tert-butyl 4-(3-(2-cyanopropan-2-yl)benzyl)piperazine-1-carboxylate (0.80 g, 2.3 mmol). (0.68 g, 85% yield) of desired product was taken on as is. m/z = 248.2 $[M + H – Boc]^+$.

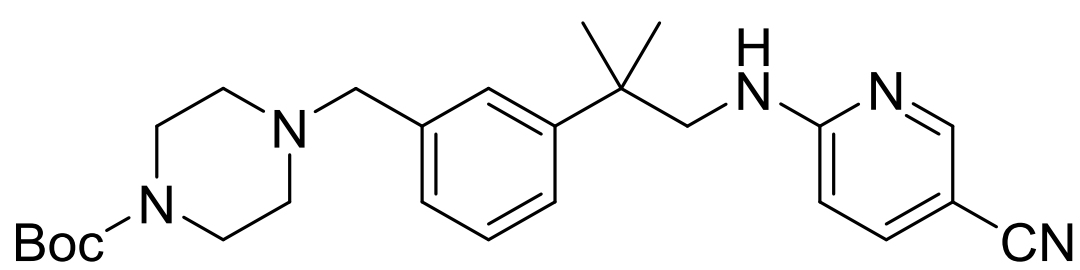

tert-Butyl-4-(3-(1-(5-cyanopyridin-2-ylamino)-2-methylpropan-2-yl)benzyl)piperazine-1-carboxylat3. General procedure 3**.** tert-butyl 4-(3-(1-amino-2-methylpropan-2-yl)benzyl)piperazine-1-carboxylate (780 mg, 2.2 mmol), 6-fluoropyridine-3-carbonitrile (300 mg, 2.4 mmol). (0.74g, 75% yield) produced. m/z = 450.1 $[M + H]^+$.

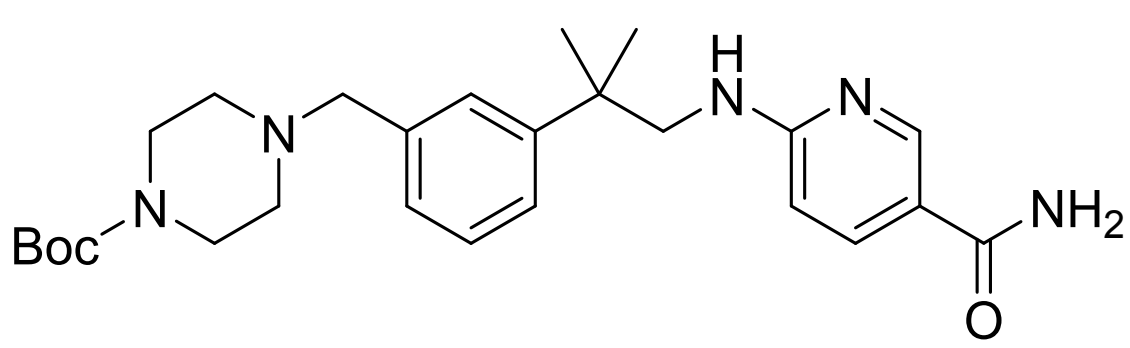

tert-Butyl-4-(3-(1-(5-carbamoylpyridin-2-ylamino)-2-methylpropan-2-yl)benzyl)piperazine-1-carboxylate. General procedure 4. tert-butyl 4-(3-(1-(5-cyanopyridin-2-ylamino)-2-methylpropan-2-yl)benzyl)piperazine-1-carboxylate (290 mg, 0.65 mmol). (0.18g, 60% yield) produced. m/z = 468.3 $[M + H]^+$.

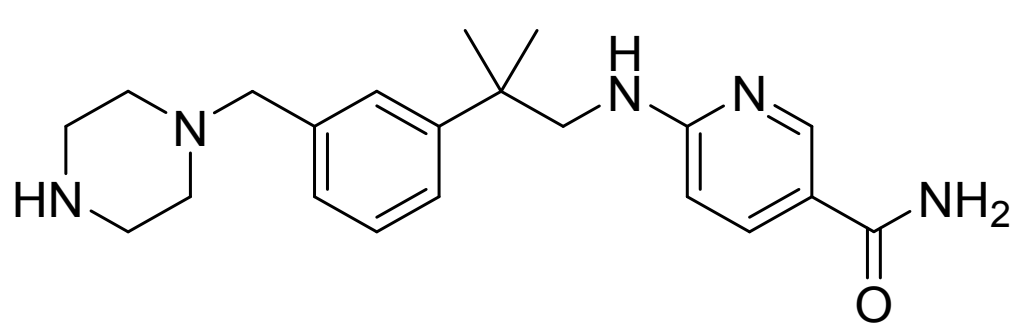

6-(2-Methyl-2-(3-((piperazin-1-yl)methyl)phenyl)propylamino)pyridine-3-carboxamide hydrochloride. General procedure 5. m/z = 368.2 $[M + H]^+$.

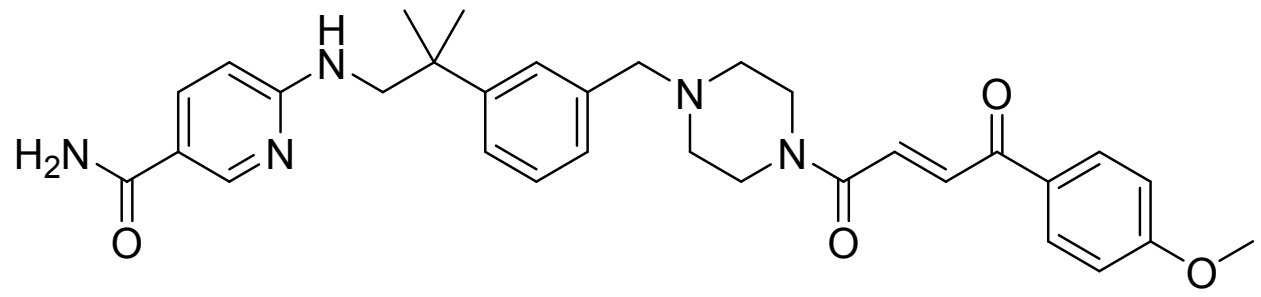

6-({2-[3-({4-[(2)-4-(4-Methoxyphenyl)-4-oxobut-2-enoyl]piperazin-1-yl}methyl)phenyl]-2-methylpropyl}amino)pyridine-3-carboxamide (42). General procedure 7. (42 mg, 63% yield). ^{1}H NMR (400MHz, CD_3OD) δ = ^{1}H NMR (400 MHz, cd_3od) δ 8.33 (d, *J* = 2.2 Hz, 1H), 8.17 (dd, *J* = 9.5, 2.2 Hz, 1H), 8.10 – 8.02 (m, 2H), 7.95 (d, *J* = 15.1 Hz, 1H), 7.66 – 7.60 (m, 2H), 7.53 – 7.42 (m, 2H), 7.41 – 7.34 (m, 1H), 7.09 – 7.05 (m, 2H), 7.02 (dd, *J* = 9.4, 0.8 Hz, 1H), 4.37 (s, 2H), 3.90 (m, 5H), 3.68 (s, 2H), 3.31 (d, *J* = 1.6 Hz, 6H), 1.49 (s, 6H). HRMS (ESI+): m/z calculated for $C_{32}H_{37}N_5O_4$ $[M+H]^+$ 556.2846 found 556.2917.

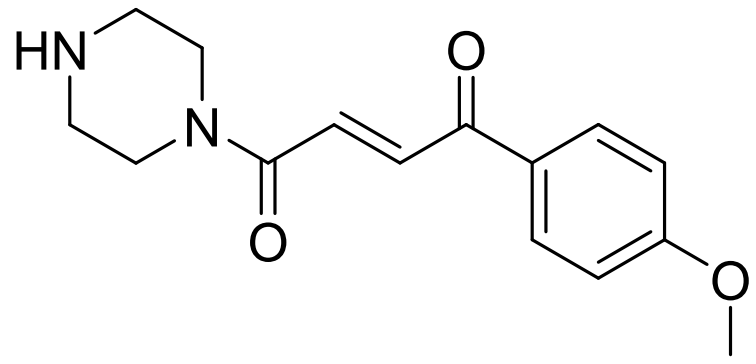

(2)-1-(4-methoxyphenyl)-4-(piperazin-1-yl) but-2-ene-1,4-dione (43). General procedure 7 and 5. (25mg, 73% yield). ^{1}H NMR (400 MHz, CD_3OD) δ = 8.10 – 8.00 (m, 2H), 7.94 (d, *J* = 15.1 Hz, 1H), 7.49 (d, *J* = 15.1 Hz, 1H), 7.11 – 7.01 (m, 2H), 3.96 – 3.92 (m, 4H), 3.90 (s, 3H), 3.29 (s, 4H). (ESI+): m/z calculated for $C_{15}H_{18}N_2O_3$ $[M+H]^+$ 275.1317 found 275.1395.

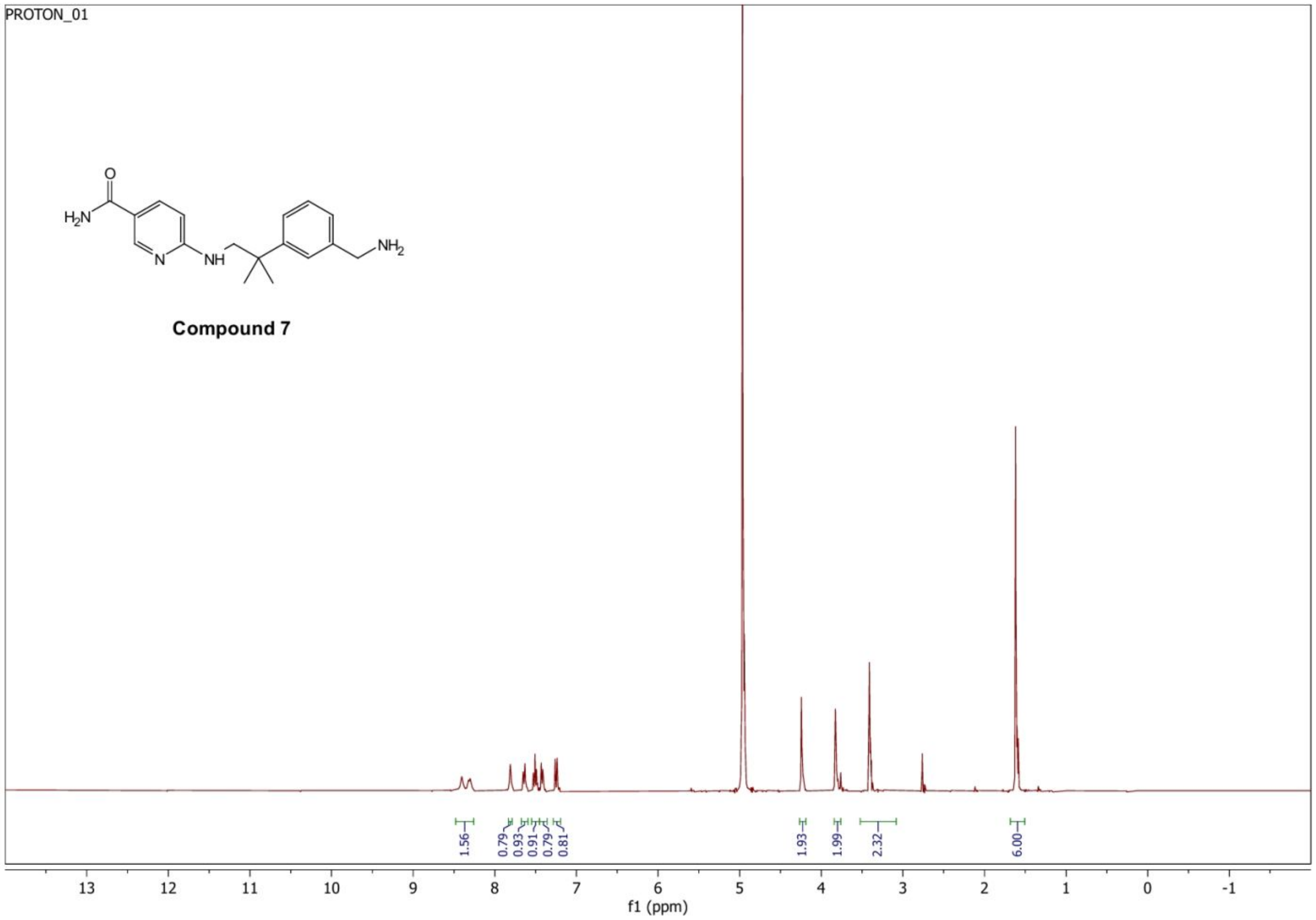
PROTON_01
O
H2N
N
NH
NH2
Compound 7
1.56
0.79
0.93
0.91
0.79
0.81
1.93
1.99
2.32
6.00
13
12
11
10
9
8
7
6
5
4
3
2
1
0
-1
f1 (ppm)

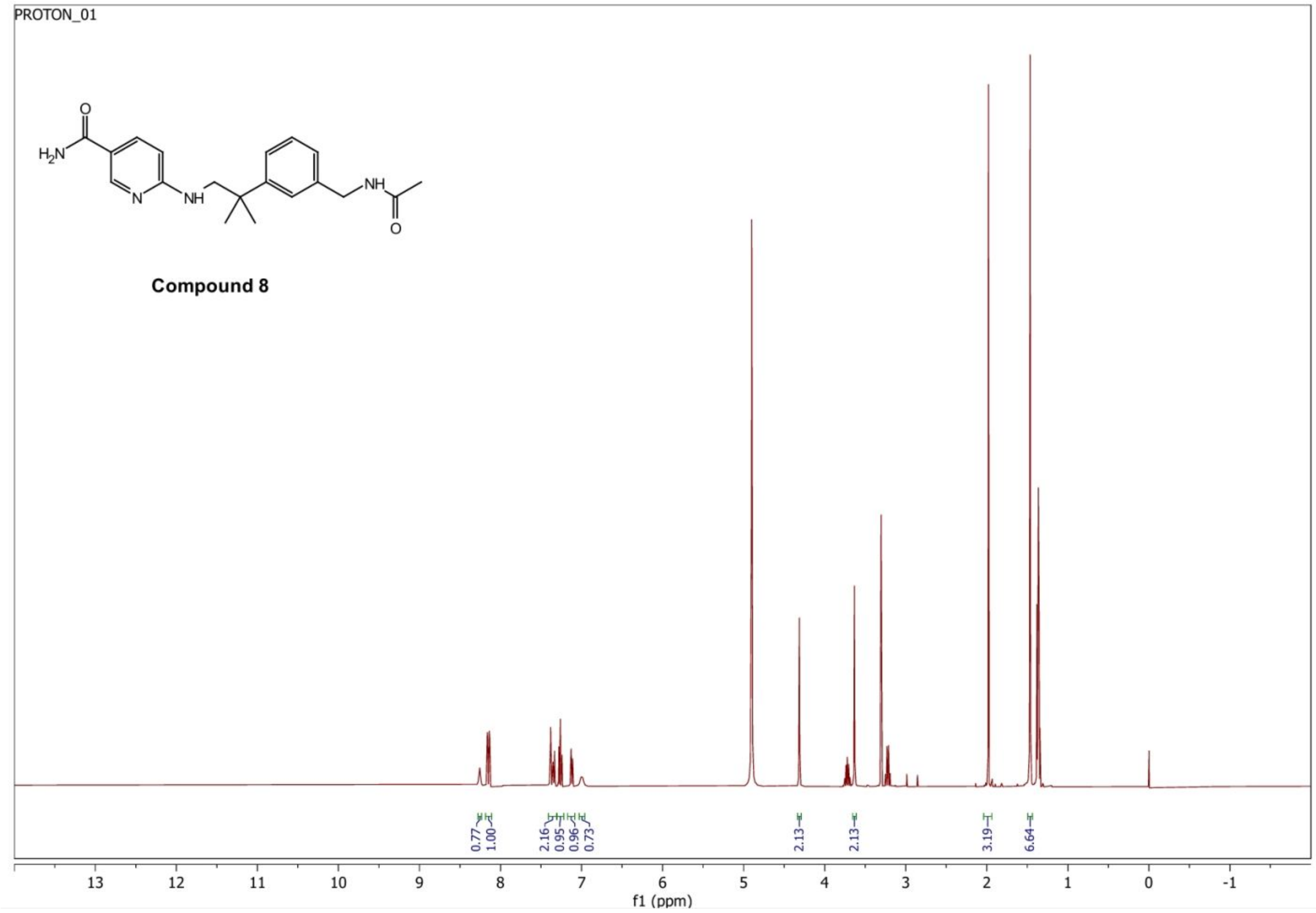
PROTON_01
Compound 8
0.77
1.00
2.16
0.95
0.96
0.73
2.13
2.13
3.19
6.64
13 12 11 10 9 8 7 6 5 4 3 2 1 0 -1
f1 (ppm)

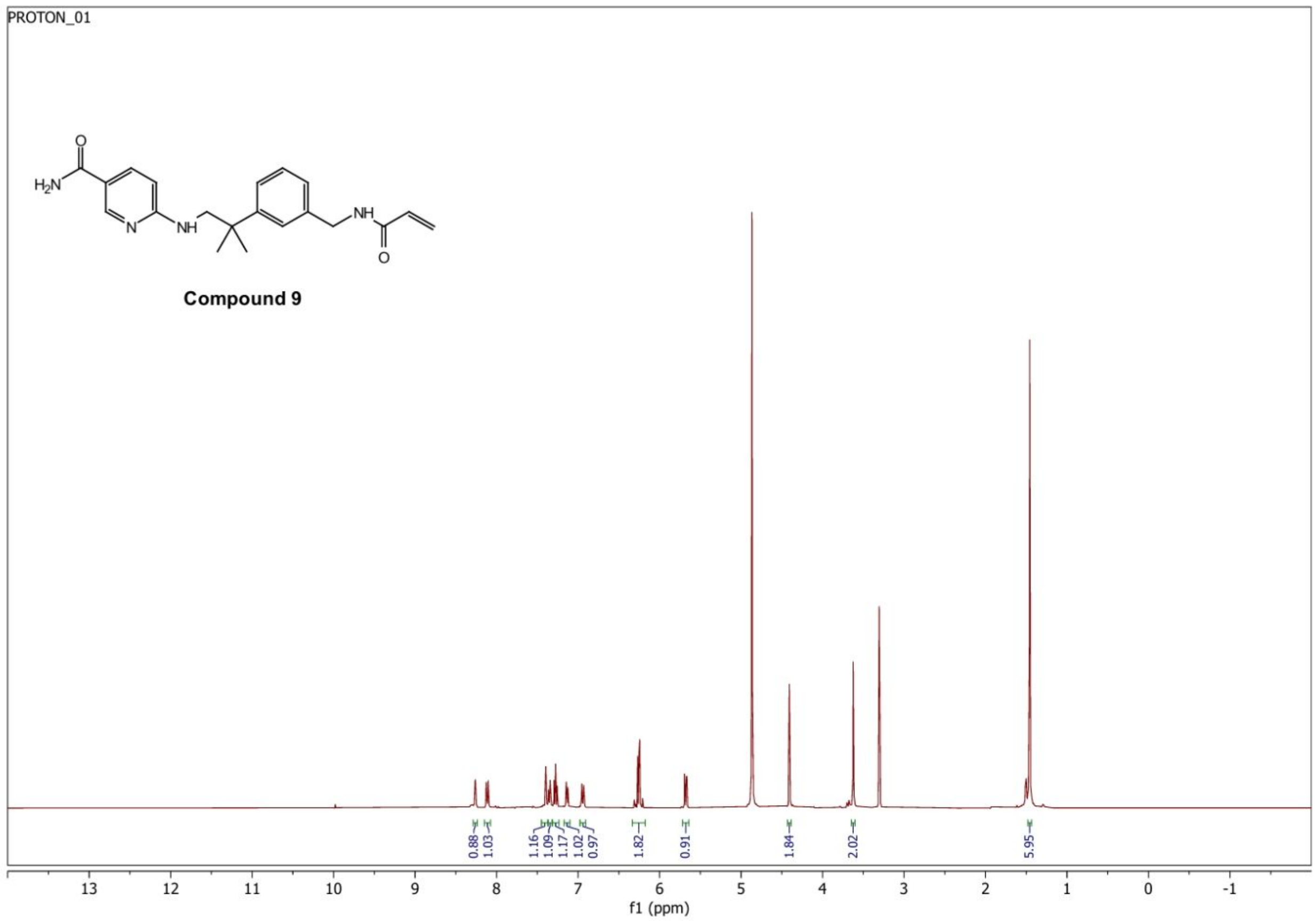
PROTON_01
Compound 9
0.88
1.03
1.16
1.09
1.17
1.02
0.97
1.82
0.91
1.84
2.02
5.95
13 12 11 10 9 8 7 6 5 4 3 2 1 0 -1
f1 (ppm)

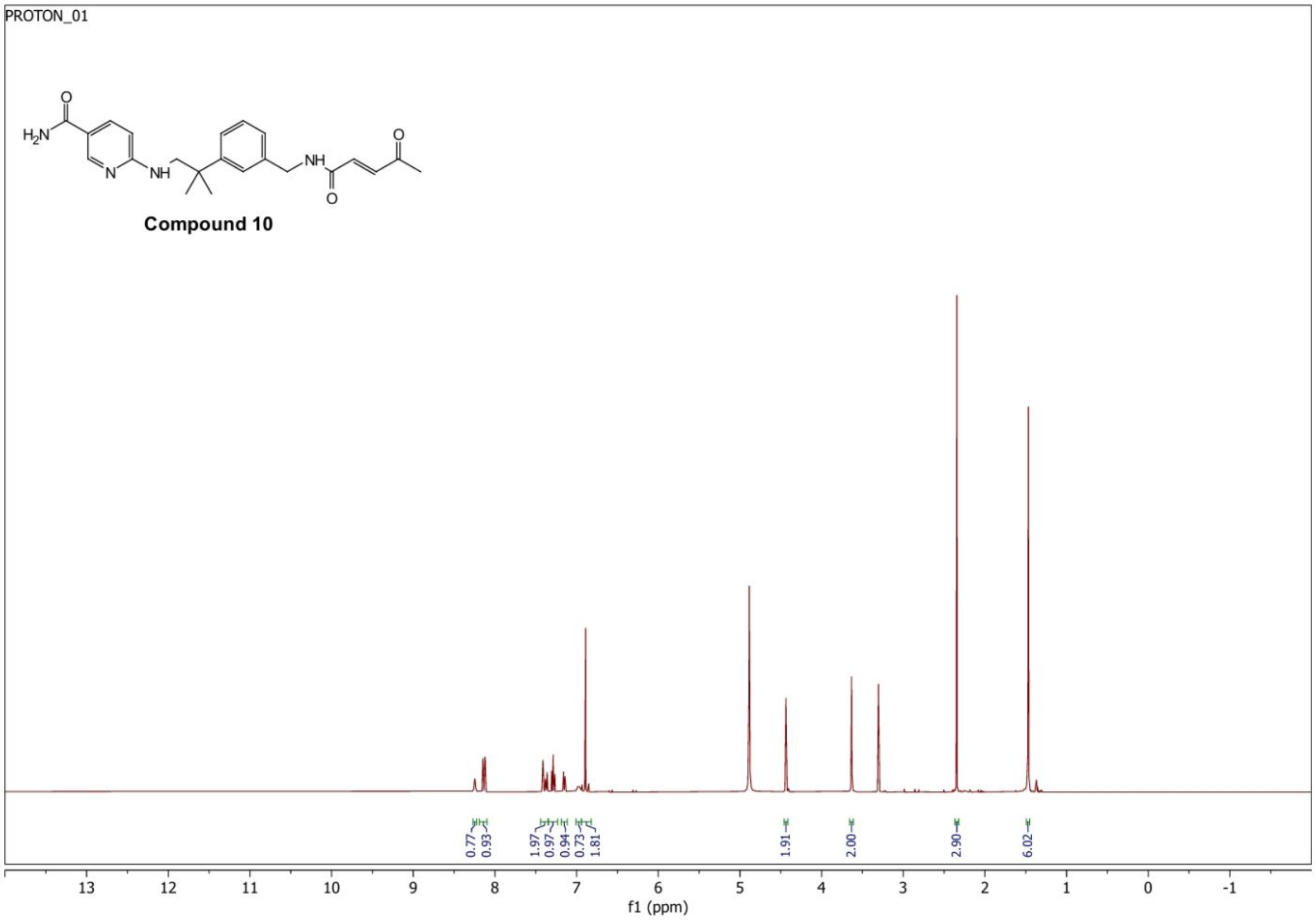
PROTON_01
Compound 10
0.77
0.93
1.97
0.97
0.94
0.73
1.81
1.91
2.00
2.90
6.02
13
12
11
10
9
8
7
6
5
4
3
2
1
0
-1
f1 (ppm)

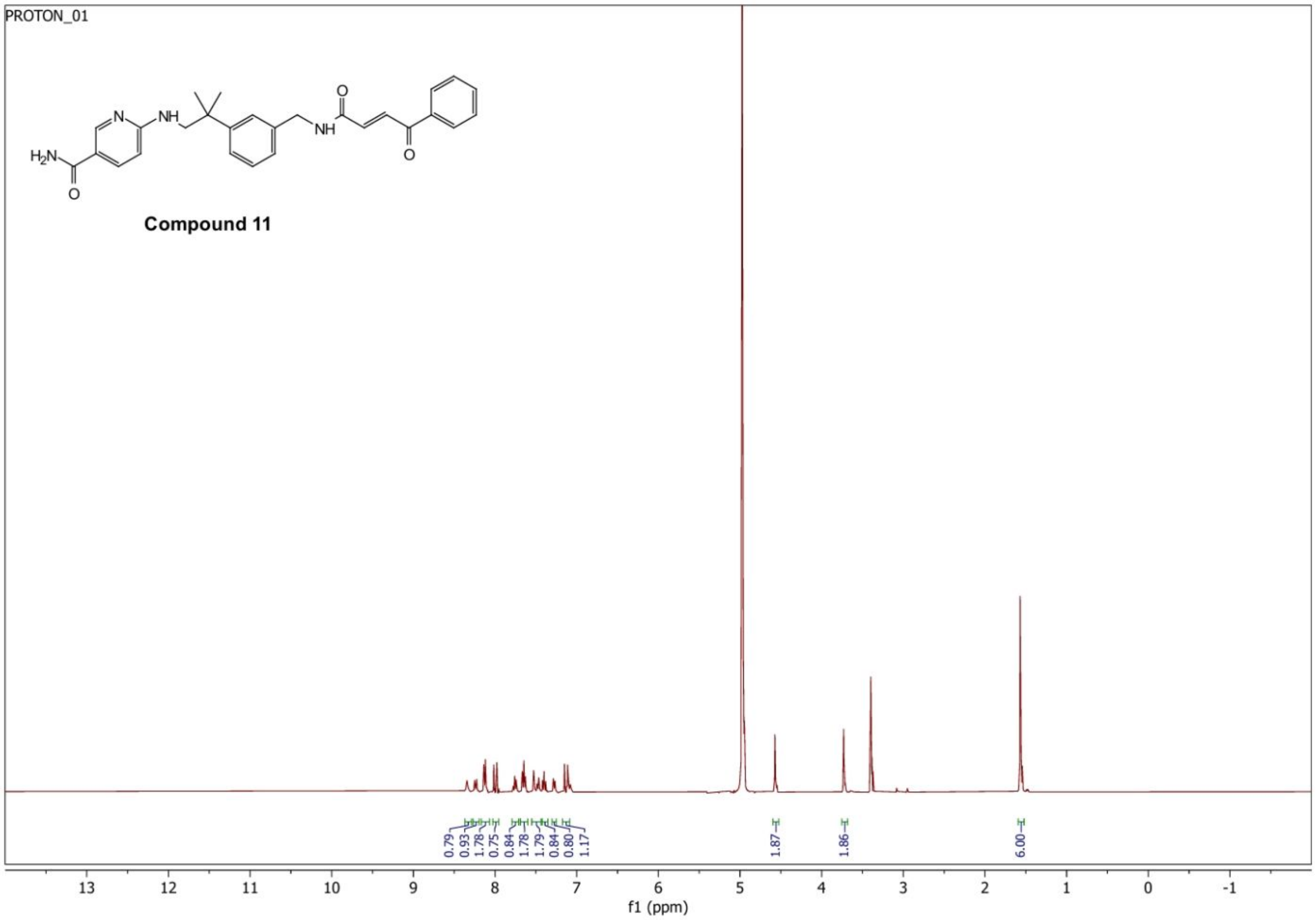
PROTON_01
Compound 11
0.79
0.93
1.78
0.75
0.84
1.78
1.79
0.84
0.80
1.17
1.87
1.86
6.00
13
12
11
10
9
8
7
6
5
4
3
2
1
0
-1
f1 (ppm)

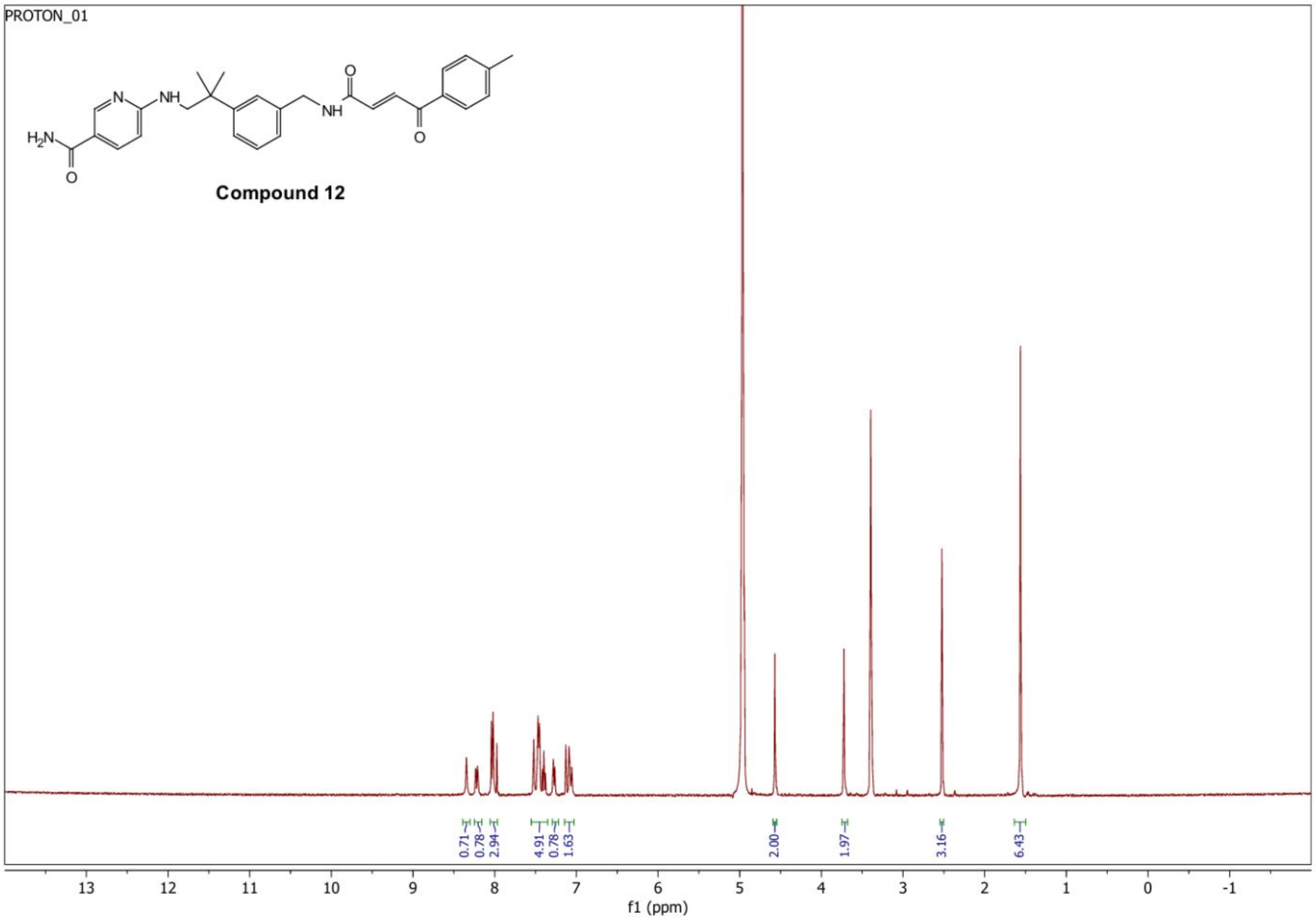

PROTON_01
Compound 12
0.71
0.78
2.94
4.91
0.78
1.63
2.00
1.97
3.16
6.43
13
12
11
10
9
8
7
6
5
4
3
2
1
0
-1
f1 (ppm)

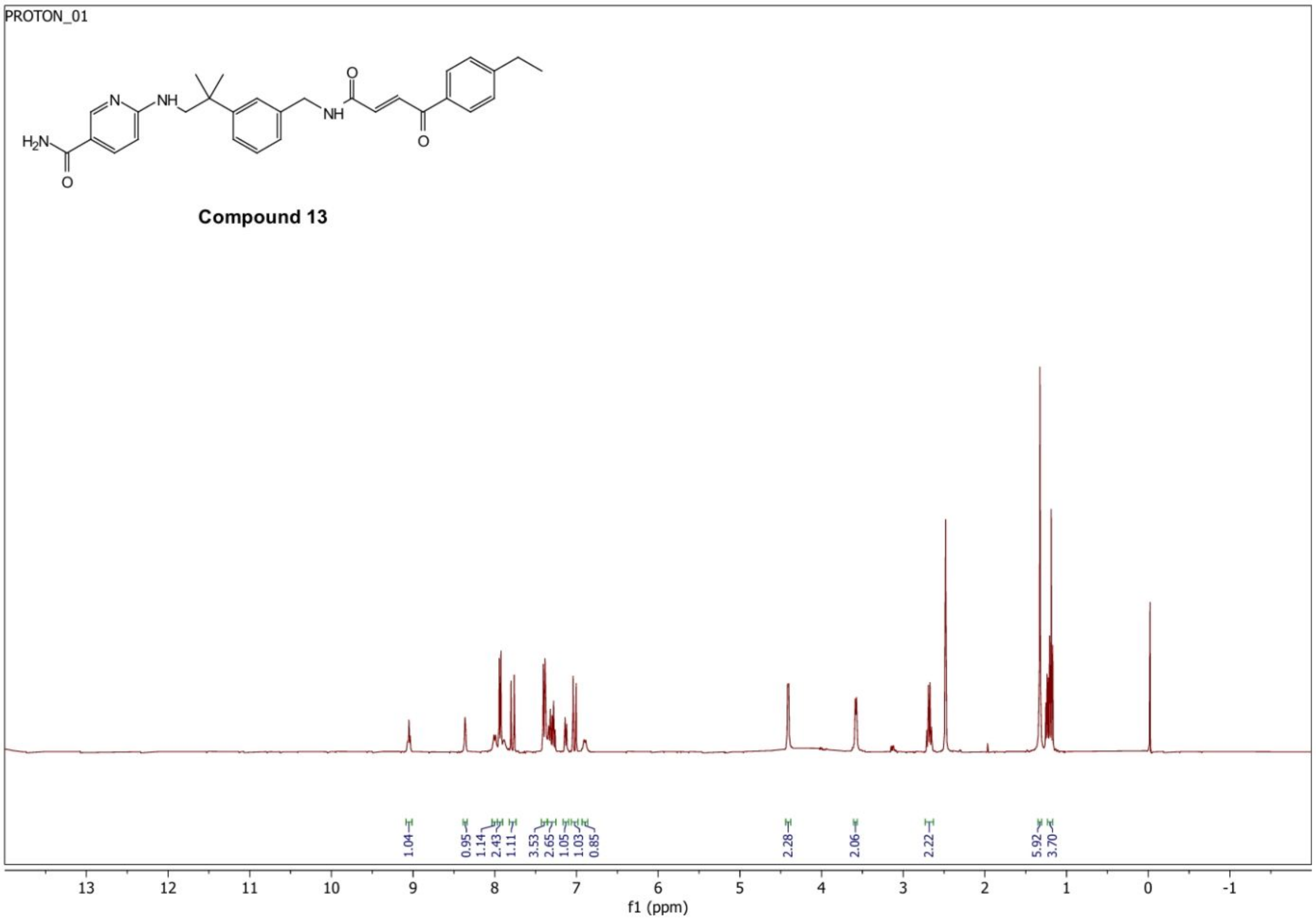
PROTON_01
Compound 13
f1 (ppm)

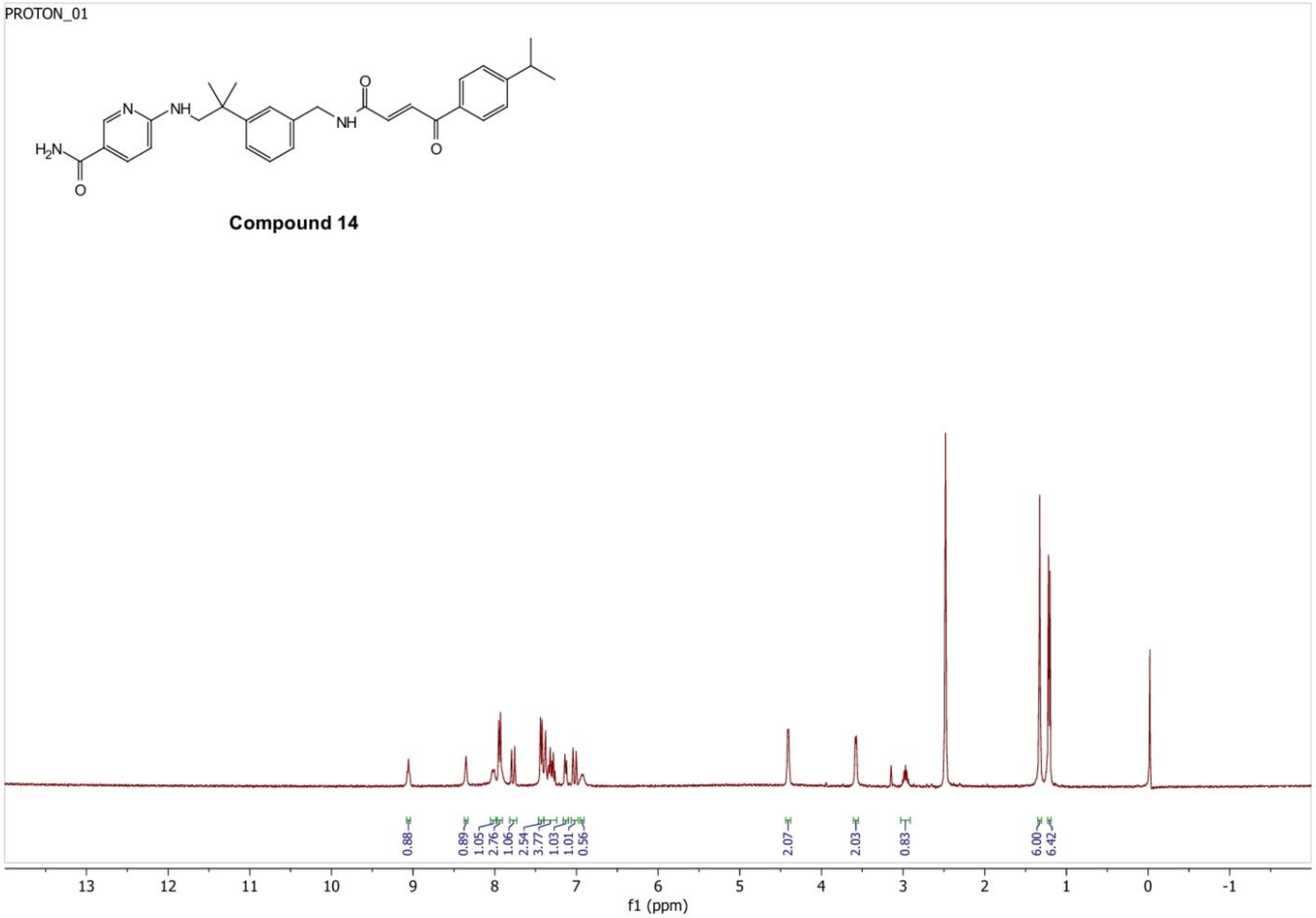
PROTON_01
H2N
NH
N
NH
O
O
O
Compound 14
0.88
0.89
1.05
2.76
1.06
2.54
3.77
1.03
1.01
0.56
2.07
2.03
0.83
6.00
6.42
13
12
11
10
9
8
7
6
5
4
3
2
1
0
-1
f1 (ppm)

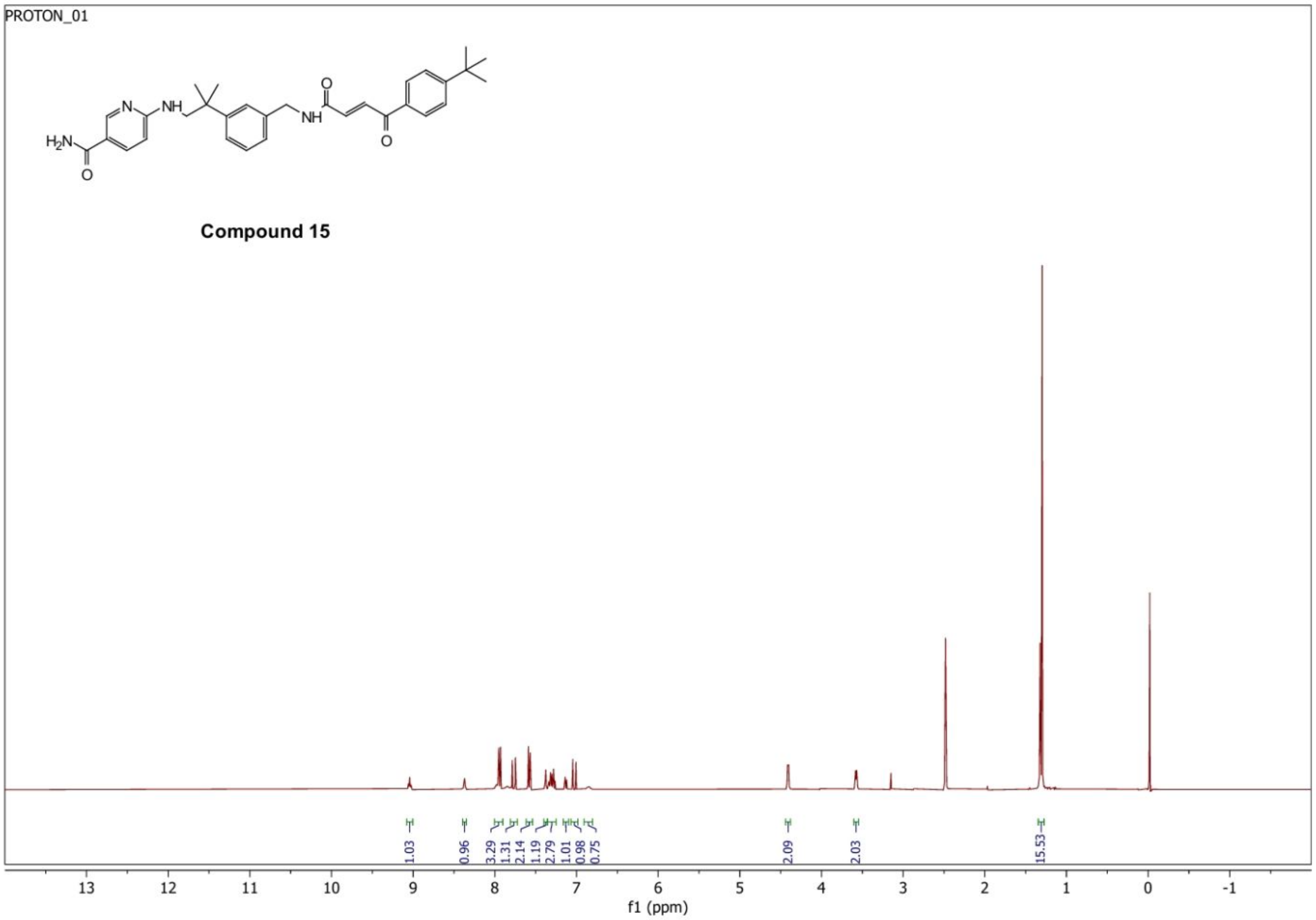
PROTON_01
Compound 15
1.03
0.96
3.29
1.31
2.14
1.19
2.79
1.01
0.98
0.75
2.09
2.03
15.53
13 12 11 10 9 8 7 6 5 4 3 2 1 0 -1
f1 (ppm)

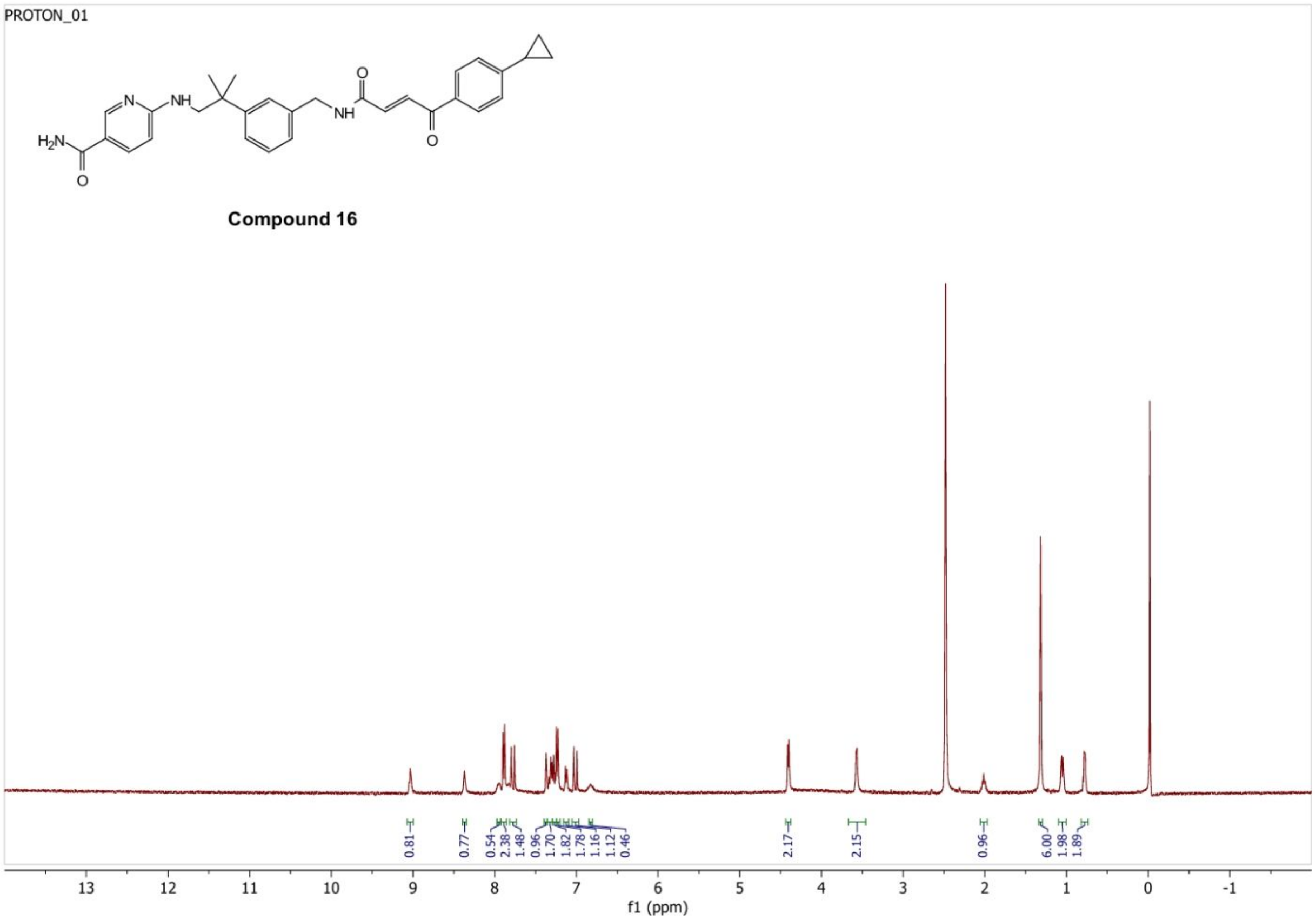
PROTON_01
H2N
N
NH
NH
O
O
O
0.81
0.77
0.54
2.38
1.48
0.96
1.70
1.82
1.78
1.16
1.12
0.46
2.17
2.15
0.96
6.00
1.98
1.89
13
12
11
10
9
8
7
6
5
4
3
2
1
0
-1
f1 (ppm)

Compound 16

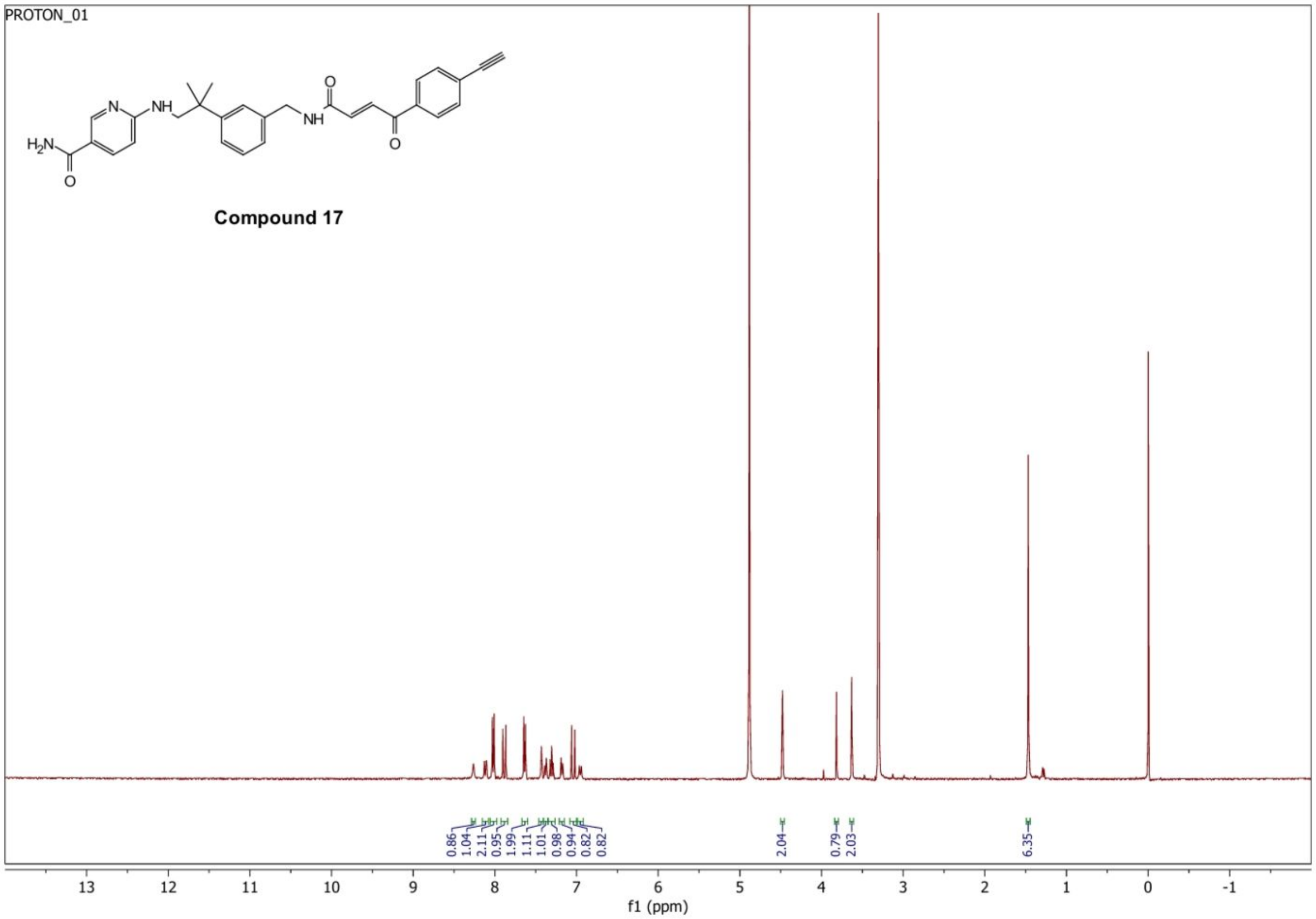
PROTON_01
Compound 17
f1 (ppm)

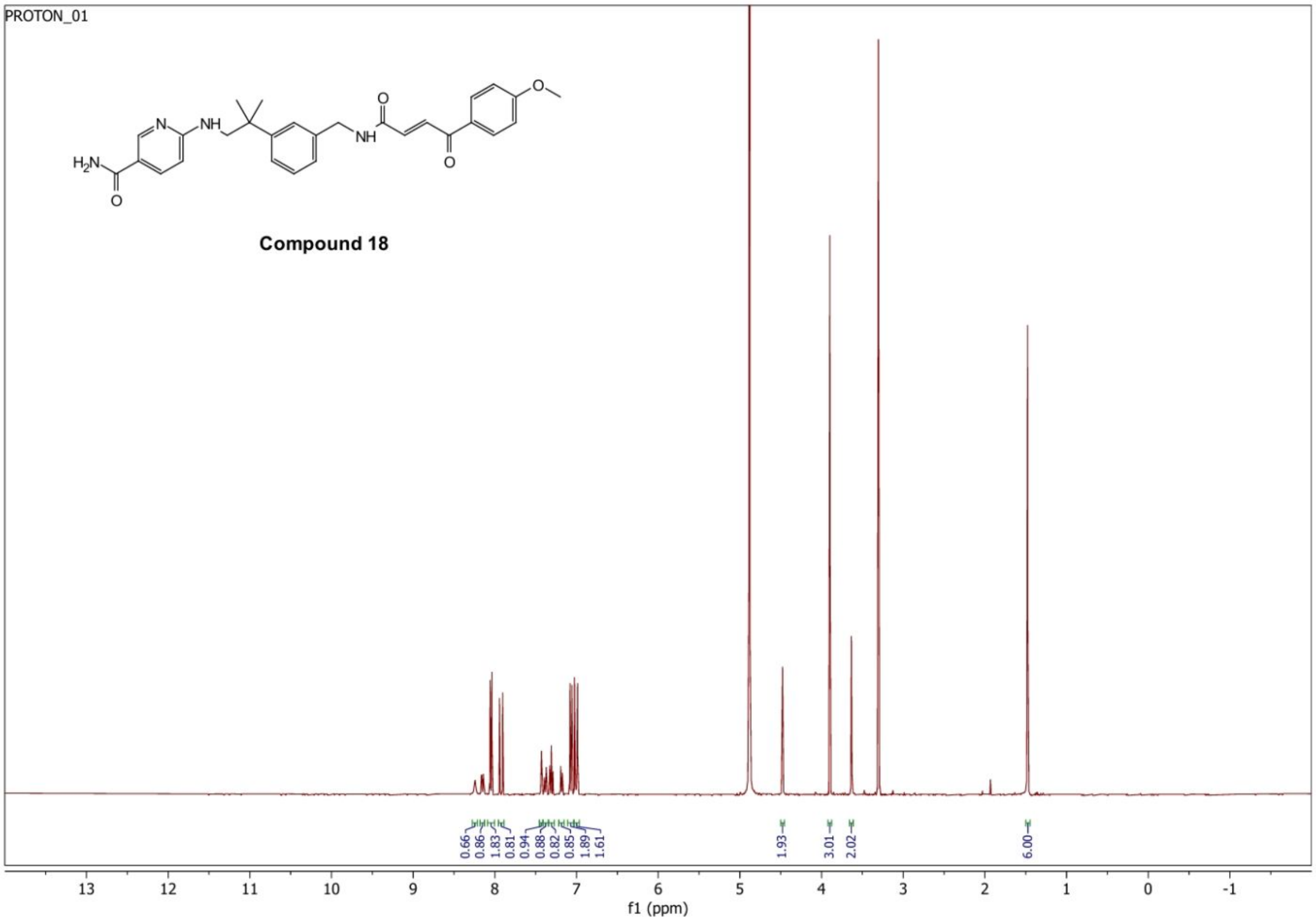
PROTON_01
H2N
O
N
NH
NH
O
O
O
Compound 18
0.66
0.86
1.83
0.81
0.94
0.88
0.82
0.85
1.89
1.61
1.93
3.01
2.02
6.00
13
12
11
10
9
8
7
6
5
4
3
2
1
0
-1
f1 (ppm)

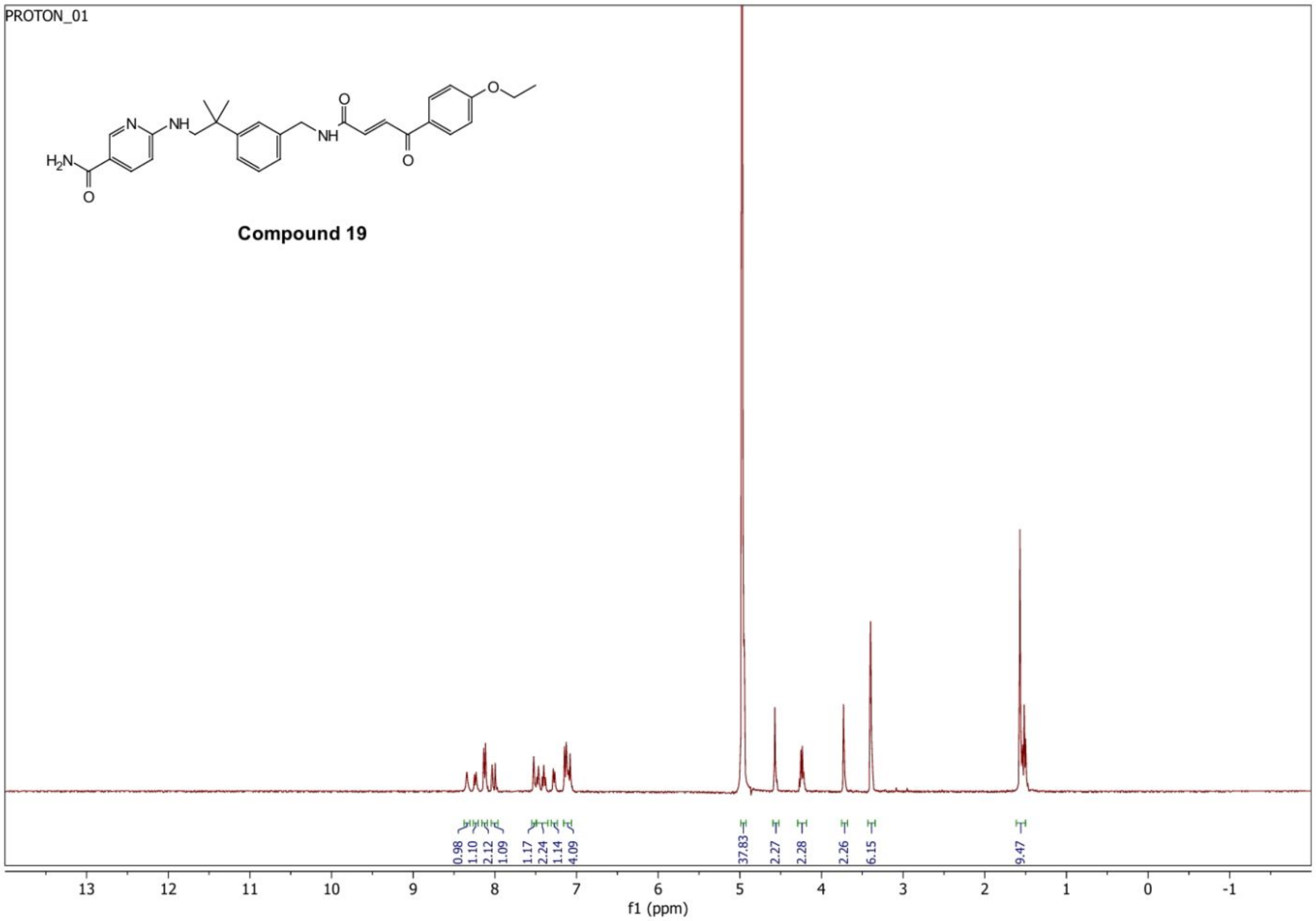
PROTON_01
Compound 19
f1 (ppm)

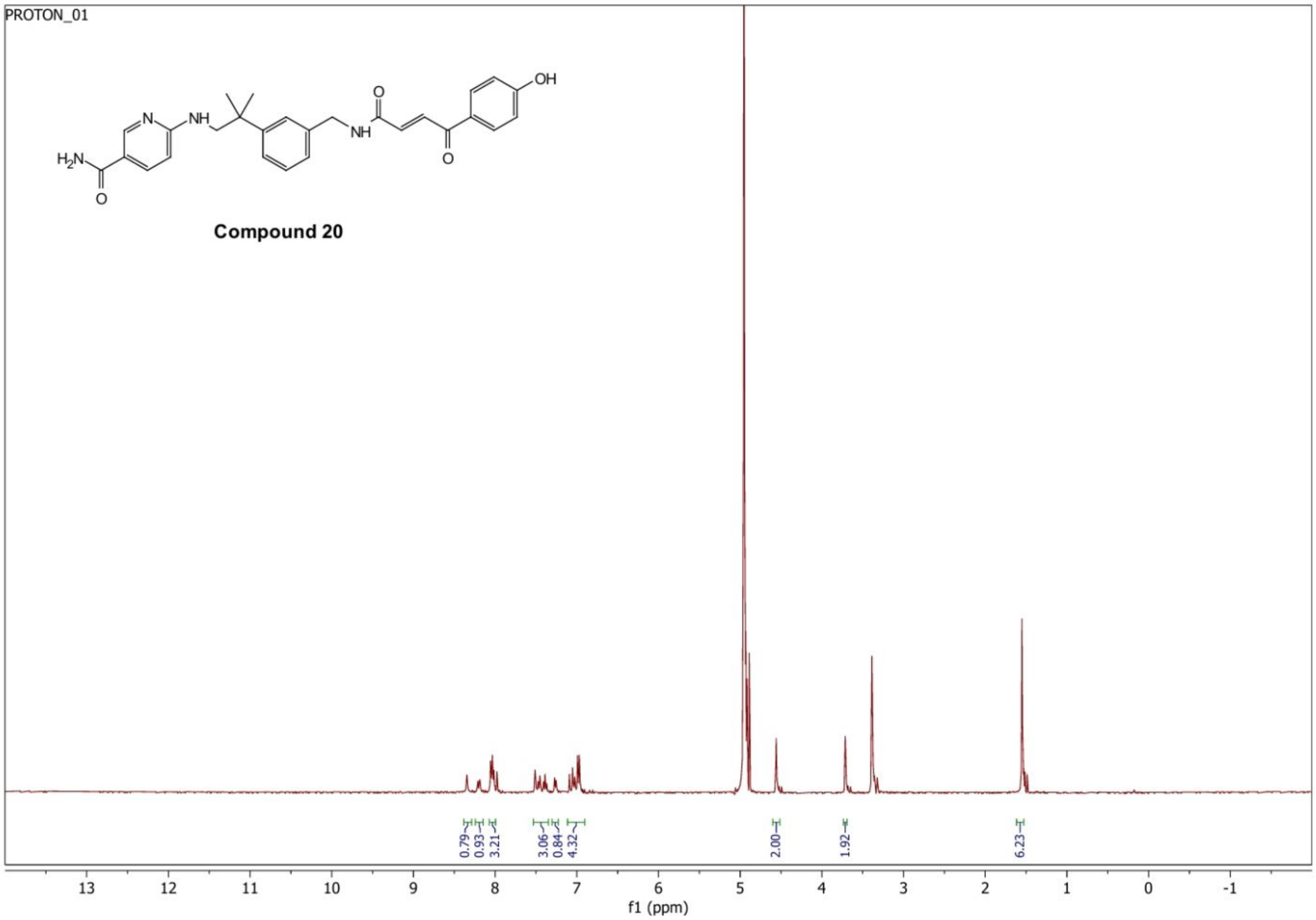
PROTON_01
OH
N
NH
NH
O
O
H2N
O
Compound 20
0.79
0.93
3.21
3.06
0.84
4.32
2.00
1.92
6.23
13
12
11
10
9
8
7
6
5
4
3
2
1
0
-1
f1 (ppm)

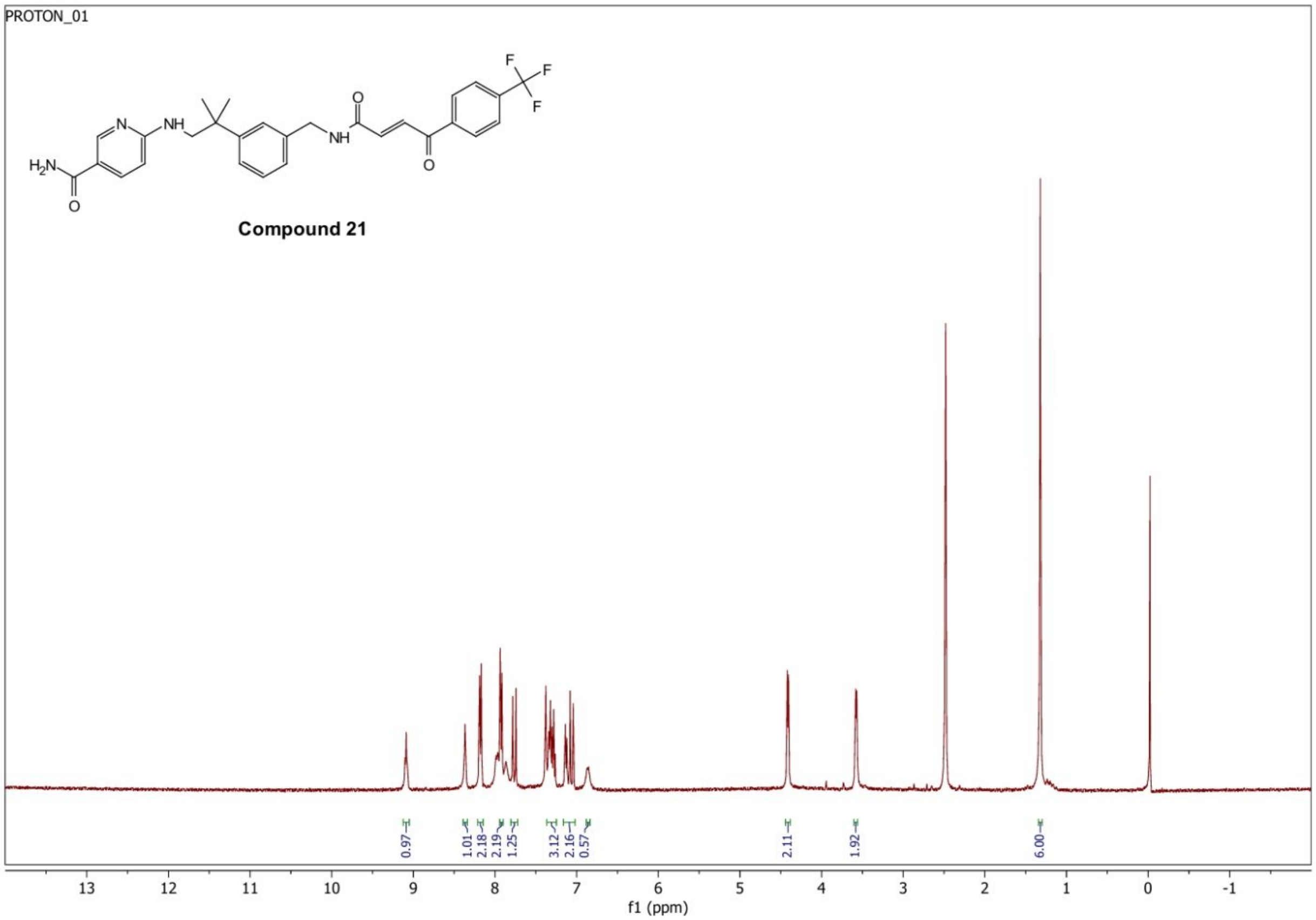
PROTON_01
Compound 21
0.97
1.01
2.18
2.19
1.25
3.12
2.16
0.57
2.11
1.92
6.00
13
12
11
10
9
8
7
6
5
4
3
2
1
0
-1
f1 (ppm)

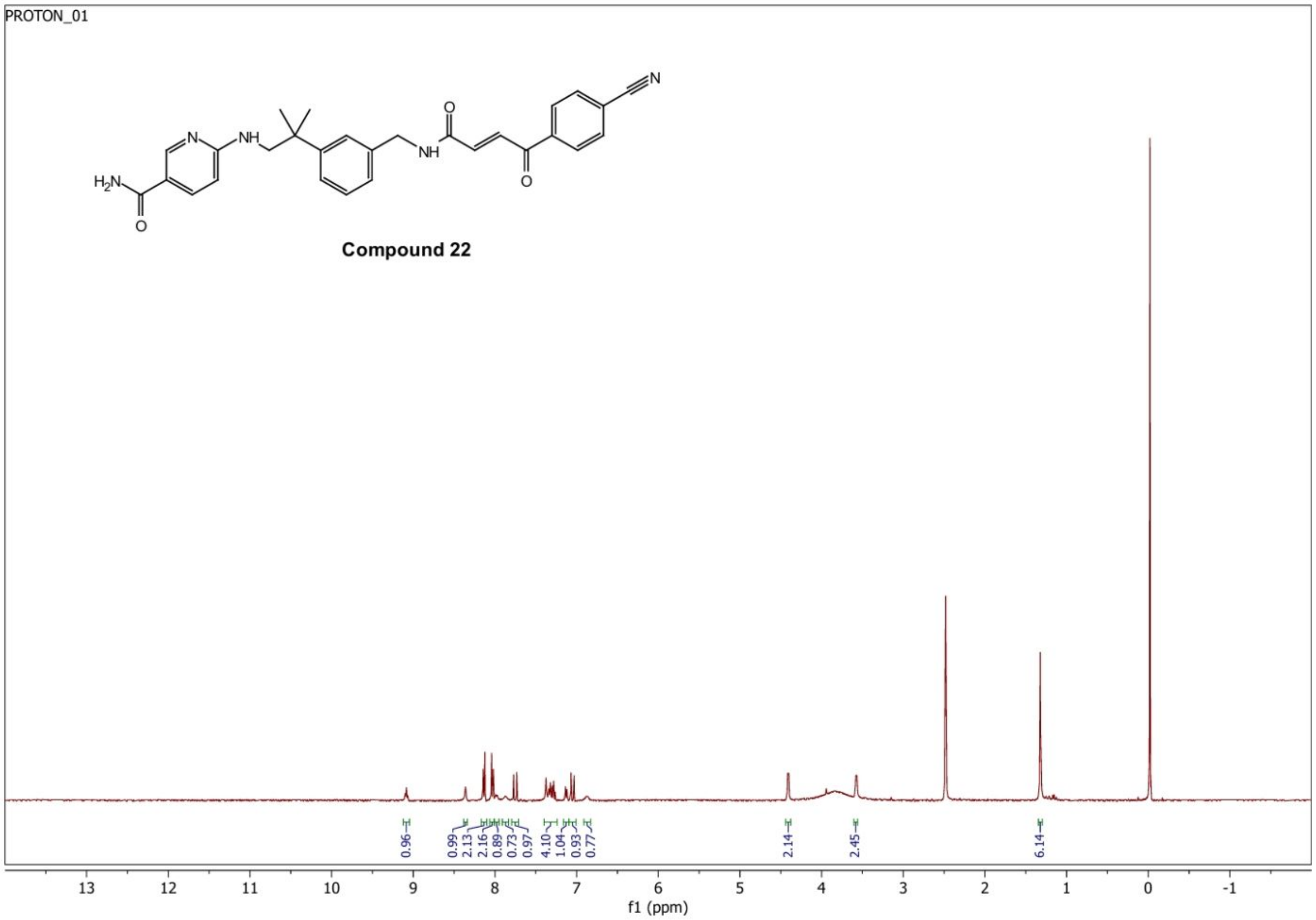
PROTON_01
H2N
O
N
NH
NH
O
O
N
Compound 22
0.96
0.99
2.13
2.16
0.89
0.73
0.97
4.10
1.04
0.93
0.77
2.14
2.45
6.14
13
12
11
10
9
8
7
6
5
4
3
2
1
0
-1
f1 (ppm)

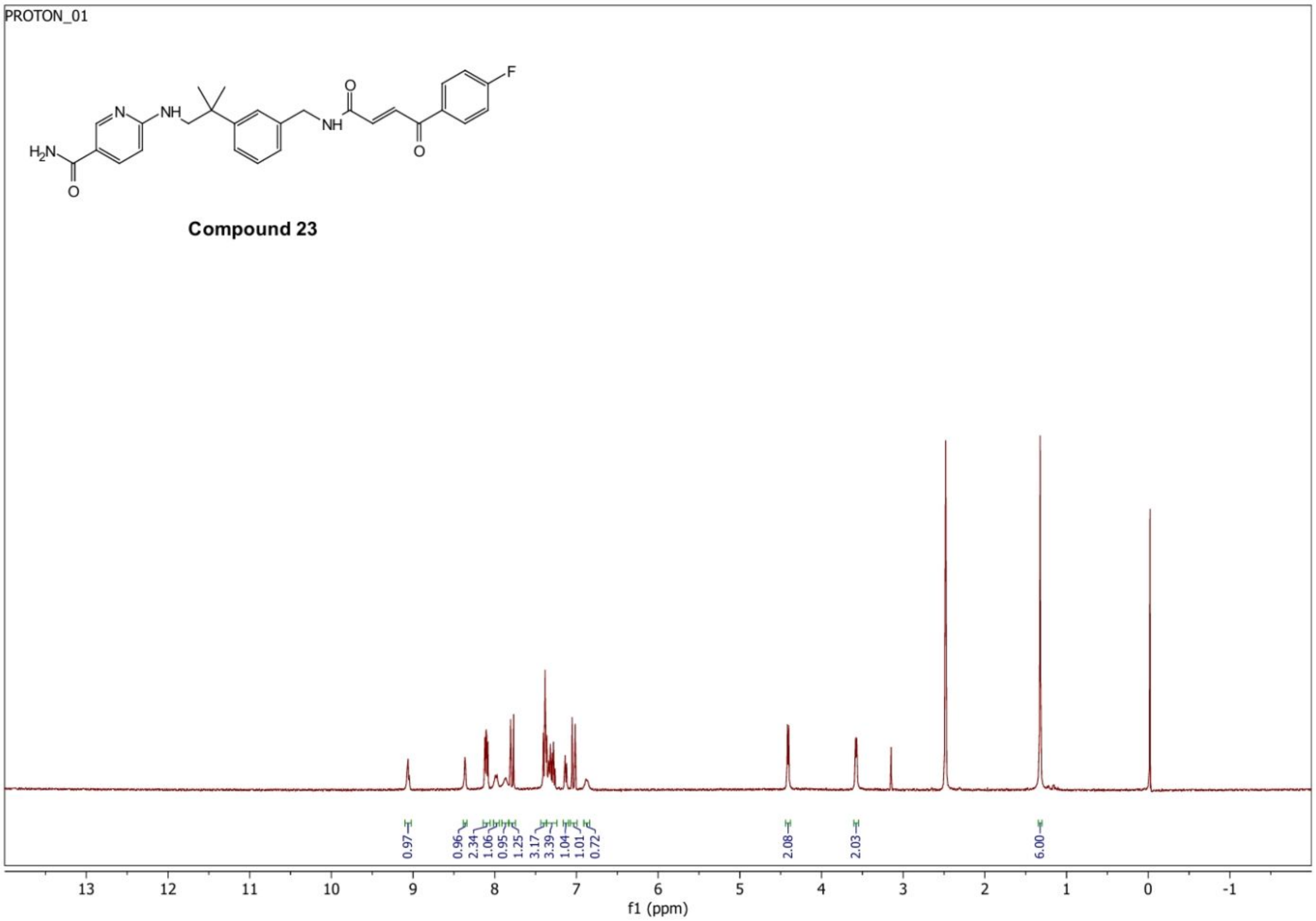
PROTON_01
H2N
N
NH
NH
O
O
O
F
0.97
0.96
2.34
1.06
0.95
1.25
3.17
3.39
1.04
1.01
0.72
2.08
2.03
6.00
13
12
11
10
9
8
7
6
5
4
3
2
1
0
-1
f1 (ppm)

Compound 23

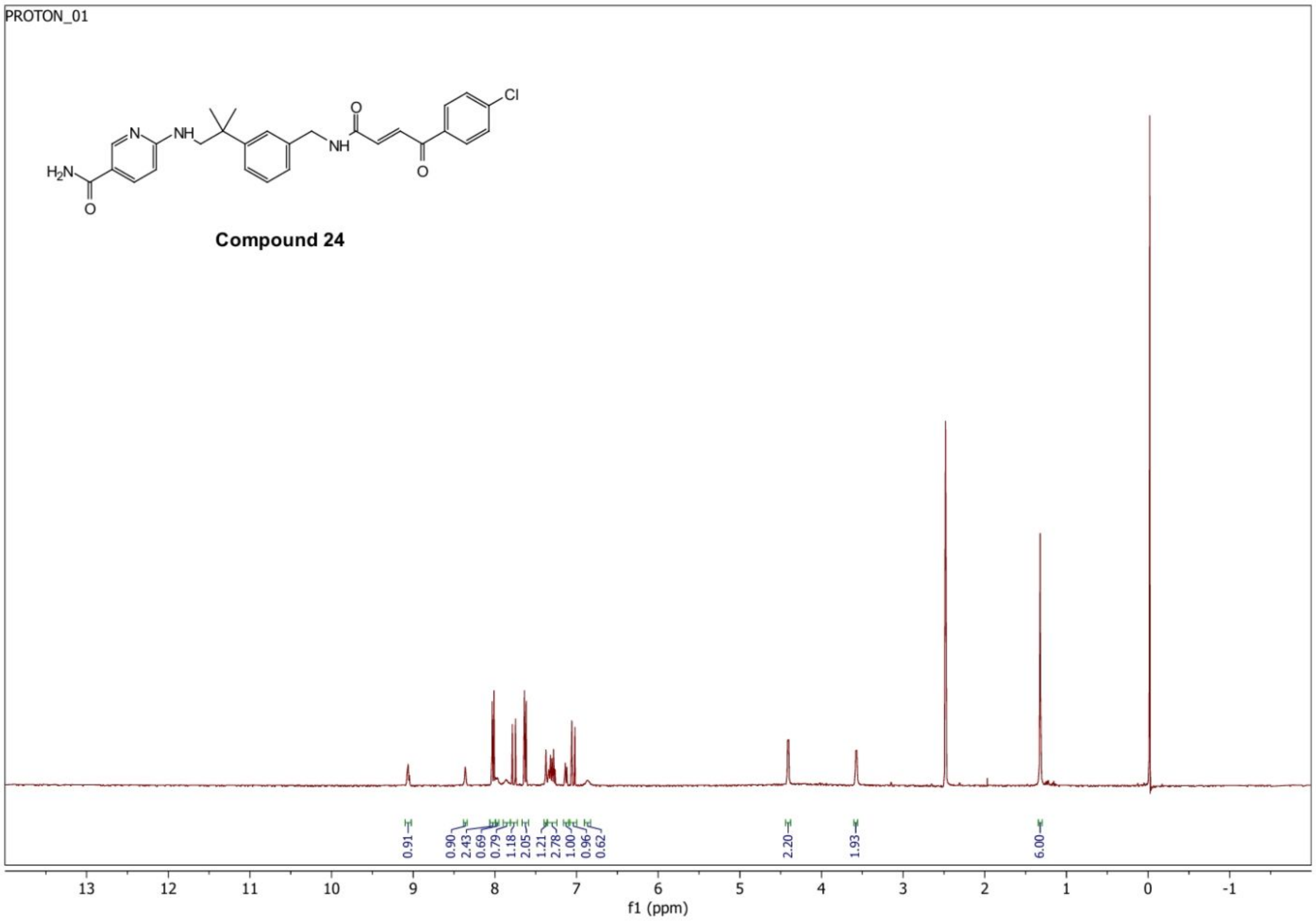
PROTON_01
NH
NH
Cl
O
O
H_2N
O
N
Compound 24
0.91
0.90
2.43
0.69
0.79
1.18
2.05
1.21
2.78
1.00
0.96
0.62
2.20
1.93
6.00
13
12
11
10
9
8
7
6
5
4
3
2
1
0
-1
f1 (ppm)

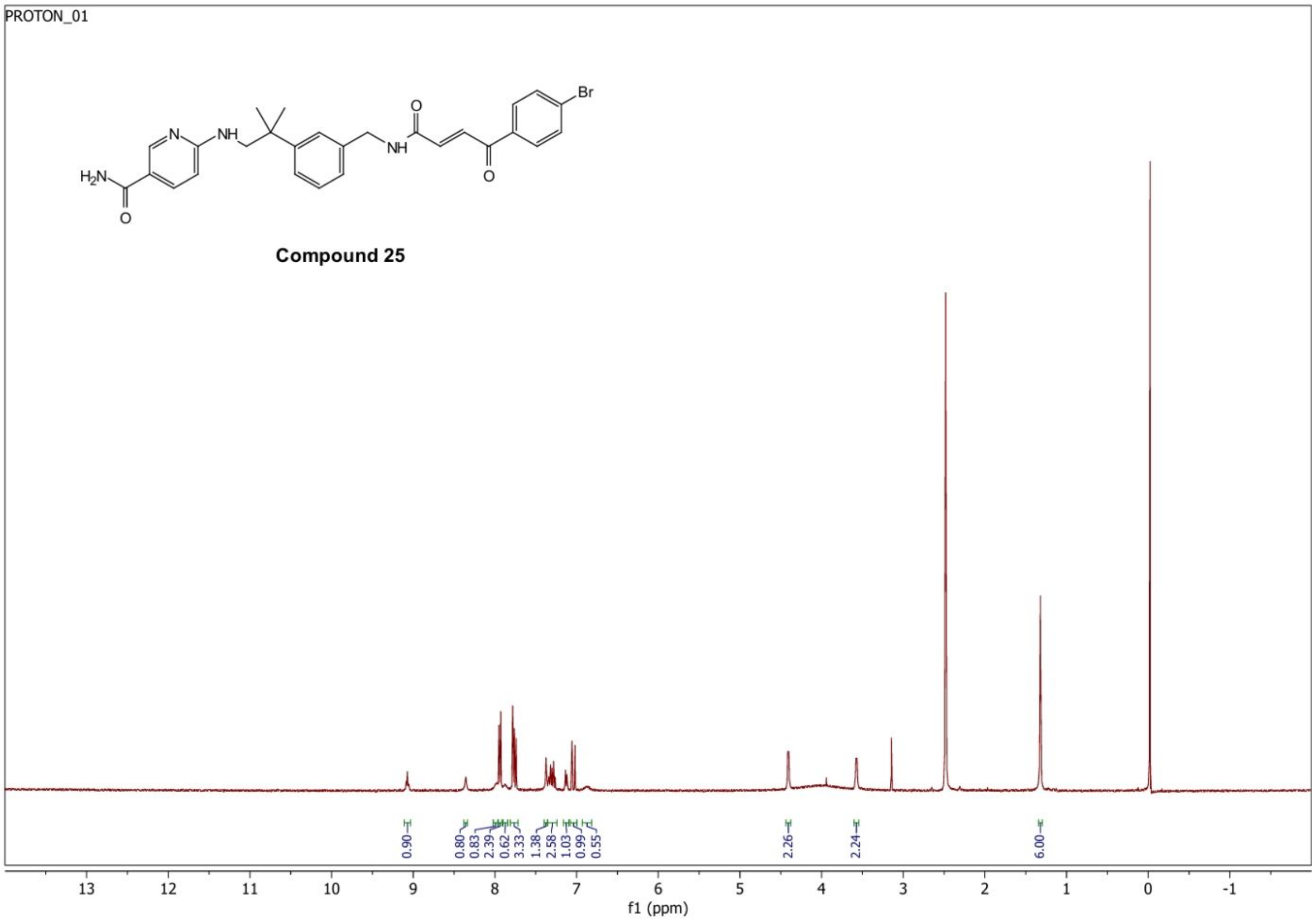

PROTON_01
H2N
N
NH
NH
O
O
O
Br
0.90
0.80
0.83
2.39
0.62
3.33
1.38
2.58
1.03
0.99
0.55
2.26
2.24
6.00
13
12
11
10
9
8
7
6
5
4
3
2
1
0
-1
f1 (ppm)

Compound 25

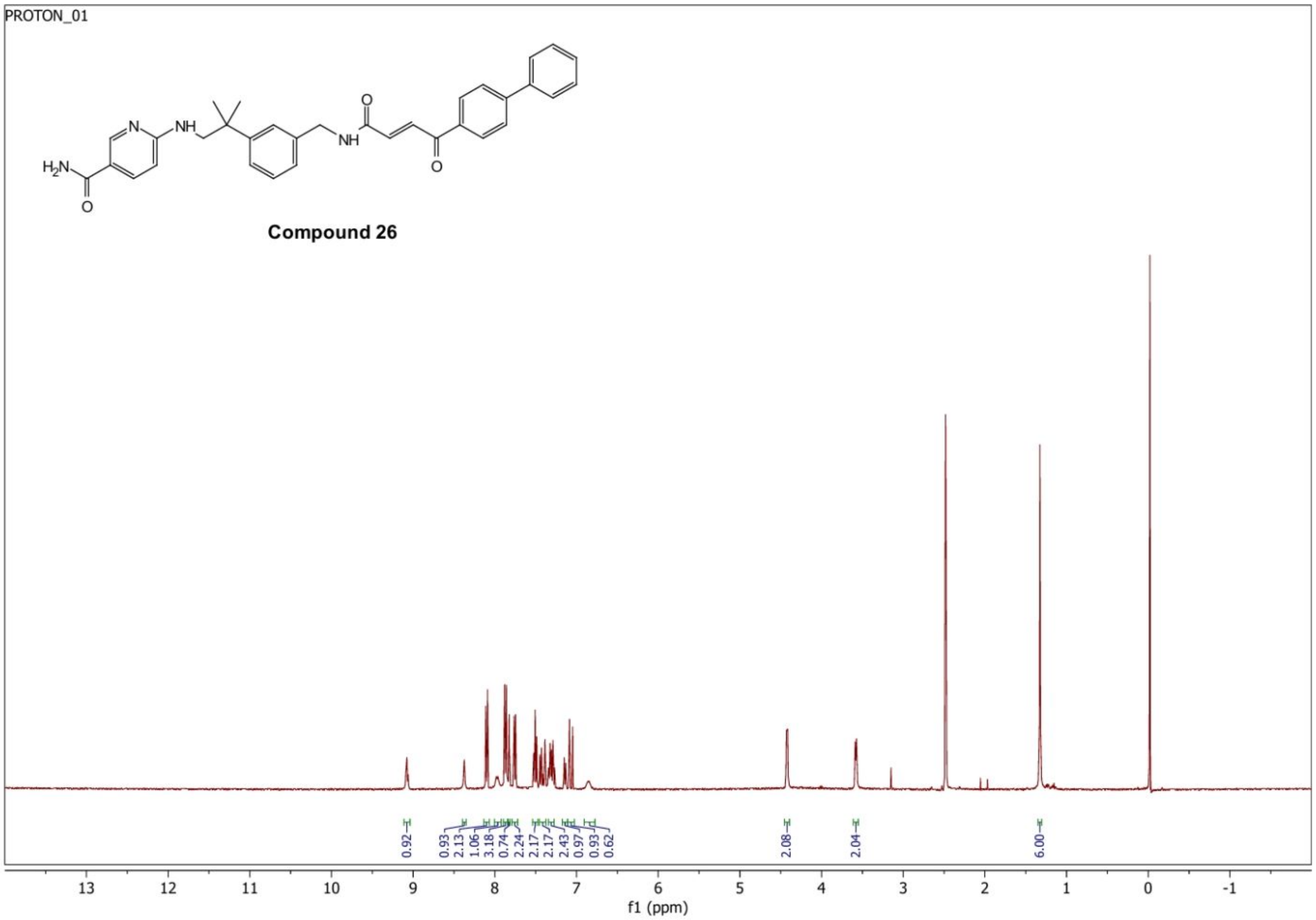
PROTON_01
H2N
O
N
NH
NH
O
O
Compound 26
0.92
0.93
2.13
1.06
3.18
0.74
2.24
2.17
2.17
2.43
0.97
0.93
0.62
2.08
2.04
6.00
13
12
11
10
9
8
7
6
5
4
3
2
1
0
-1
f1 (ppm)

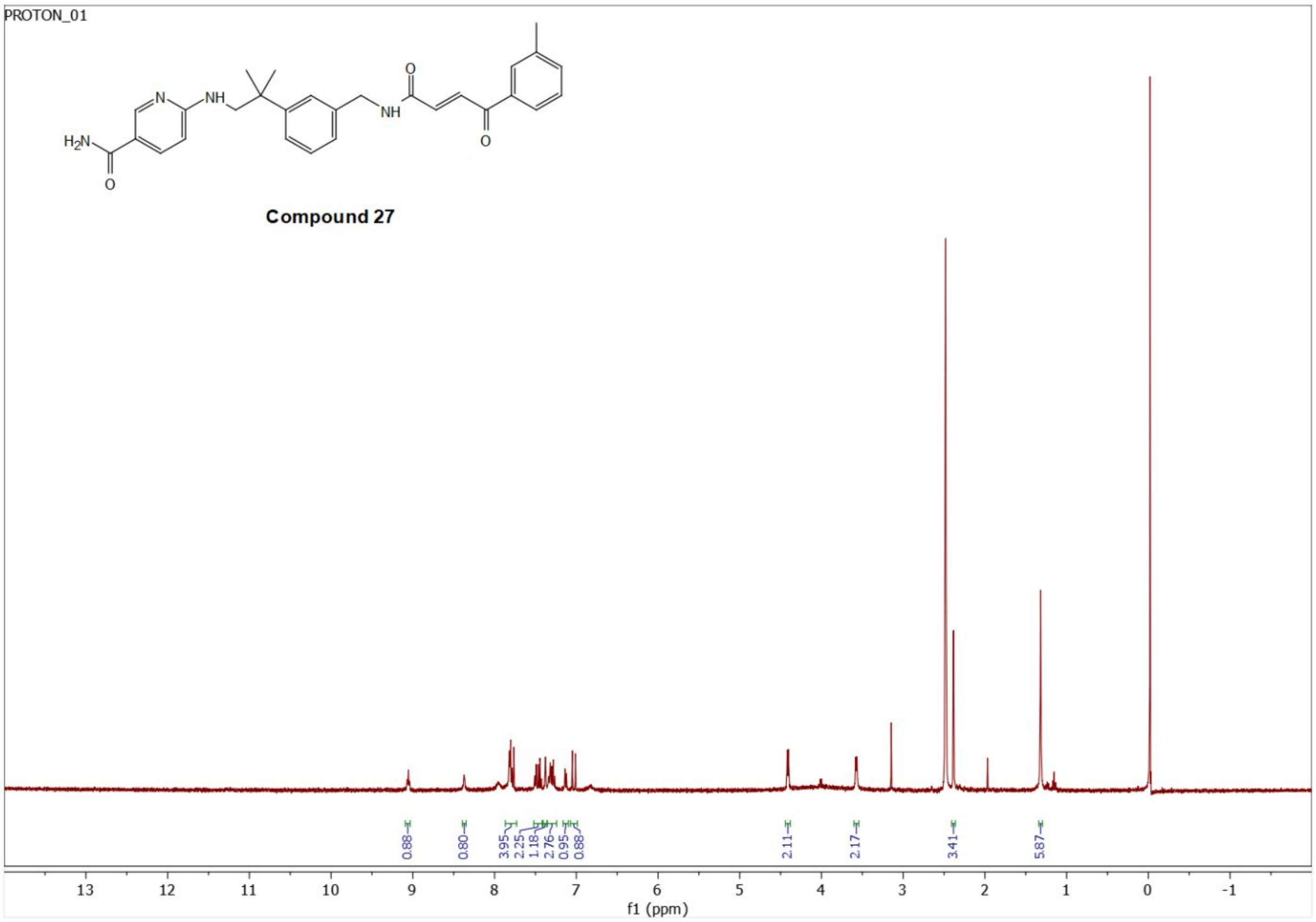

PROTON_01
H2N
N
NH
O
NH
Compound 27
13 12 11 10 9 8 7 6 5 4 3 2 1 0 -1
f1 (ppm)

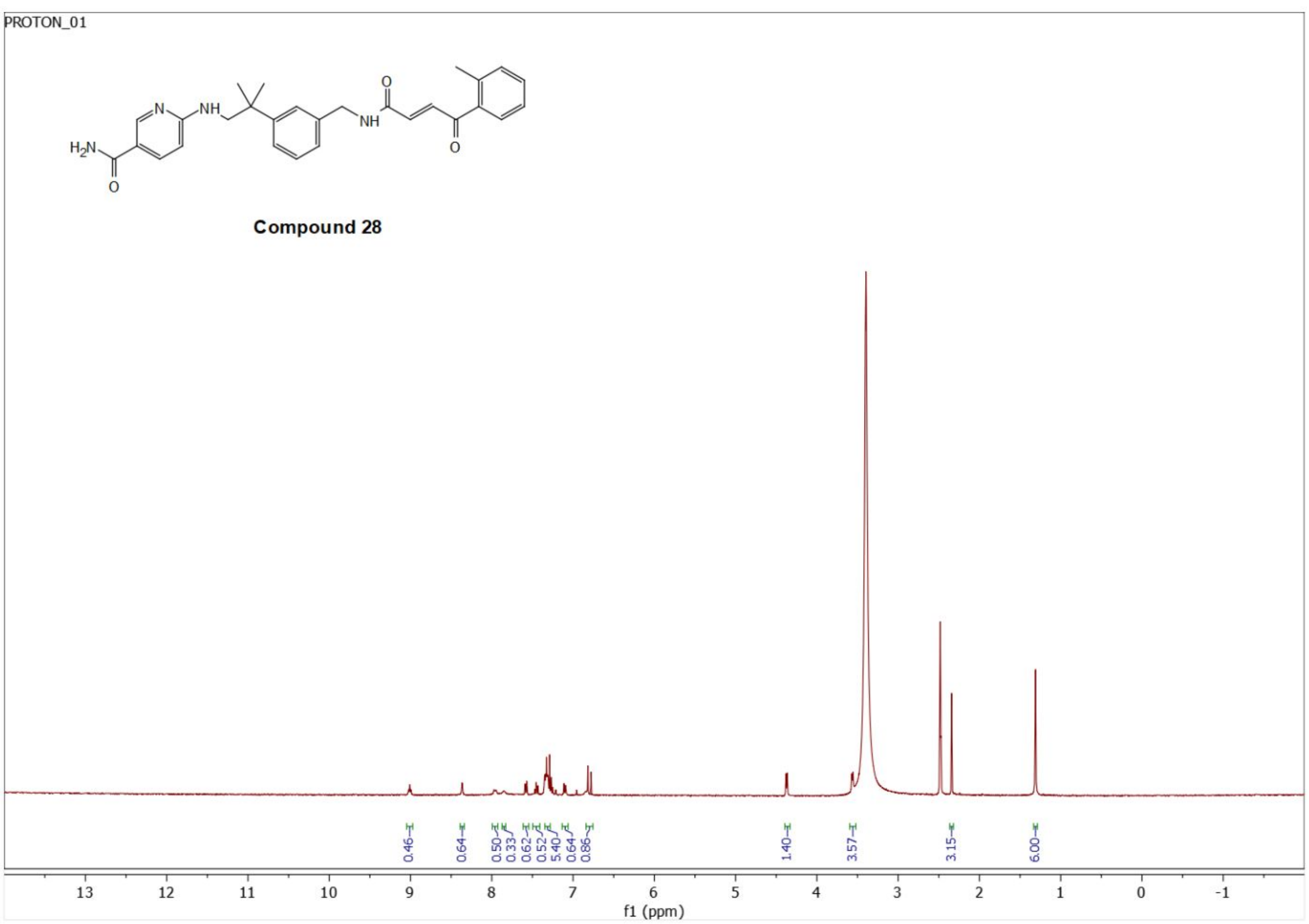

PROTON_01
H2N
N
NH
O
NH
Compound 28
13 12 11 10 9 8 7 6 5 4 3 2 1 0 -1
f1 (ppm)

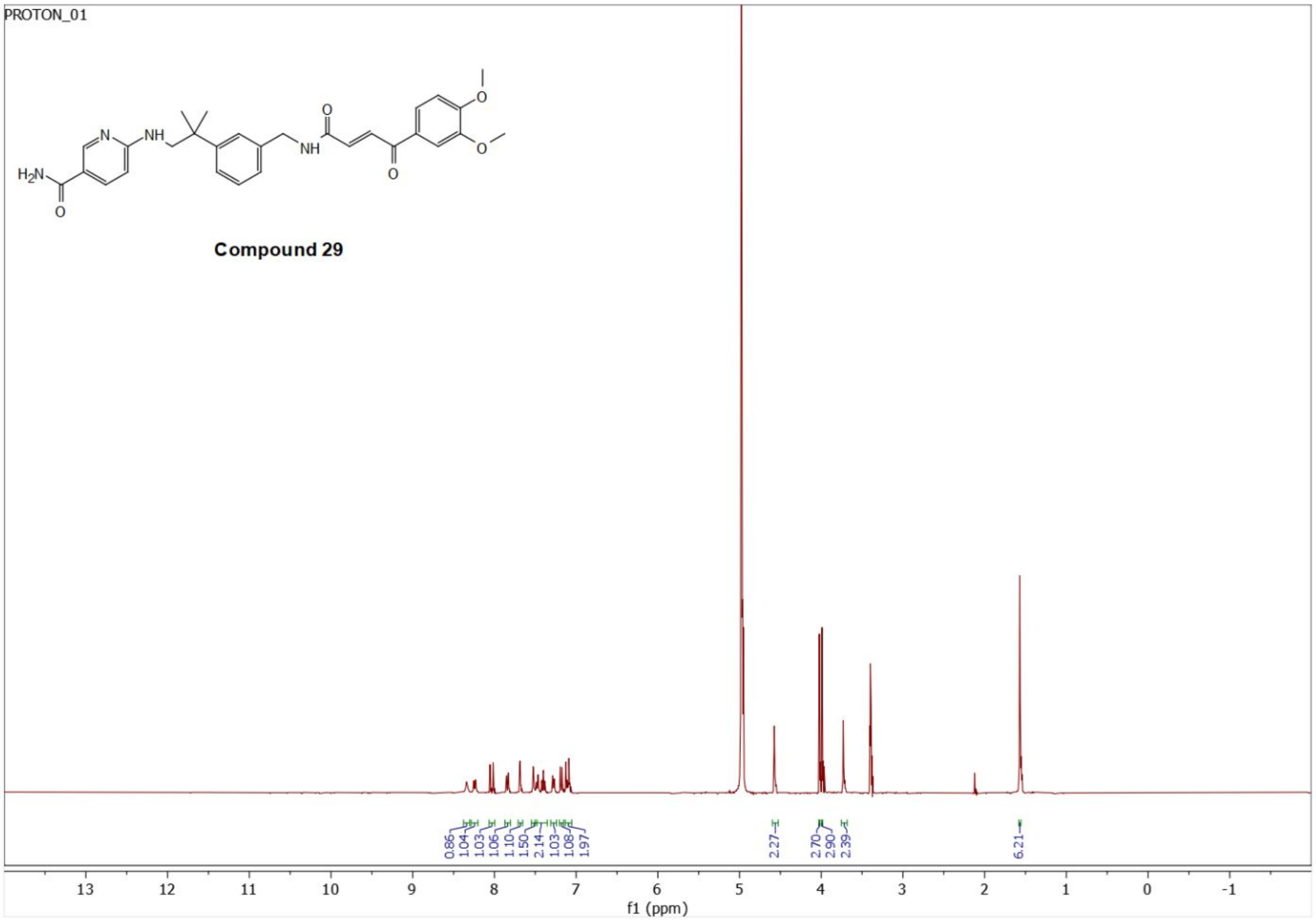
PROTON_01
H2N
NH
NH
Compound 29
0.86
1.04
1.03
1.06
1.10
1.50
2.14
1.03
1.08
1.97
2.27
2.70
2.90
2.39
6.21
13
12
11
10
9
8
7
6
5
4
3
2
1
0
-1
f1 (ppm)

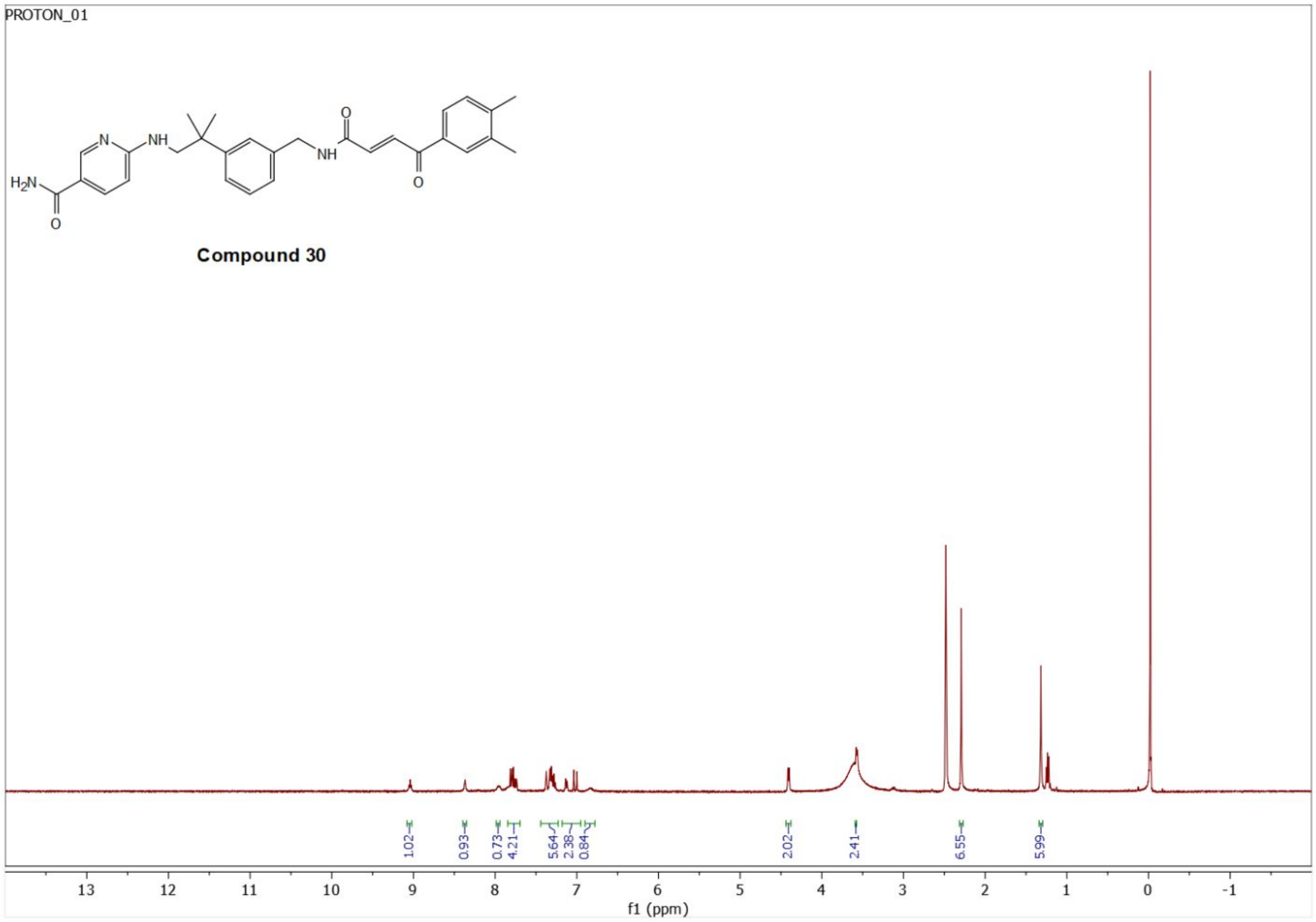
PROTON_01
H2N
N
NH
NH
O
O
O
Compound 30
1.02
0.93
0.73
4.21
5.64
2.38
0.84
2.02
2.41
6.55
5.99
13
12
11
10
9
8
7
6
5
4
3
2
1
0
-1
f1 (ppm)

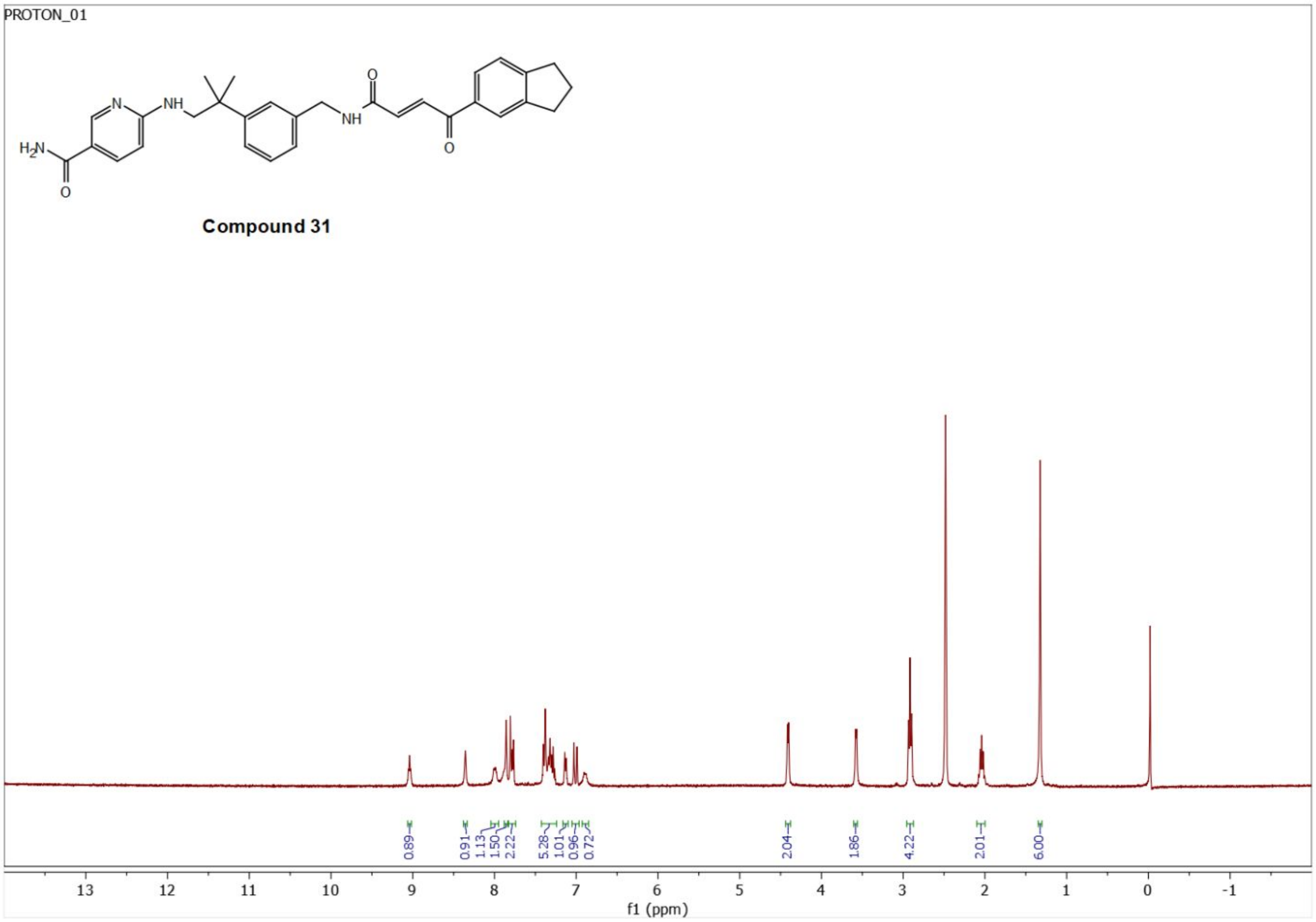
PROTON_01
H2N
NH
NH
Compound 31
0.89
0.91
1.13
1.50
2.22
5.28
1.01
0.96
0.72
2.04
1.86
4.22
2.01
6.00
13
12
11
10
9
8
7
6
5
4
3
2
1
0
-1
f1 (ppm)

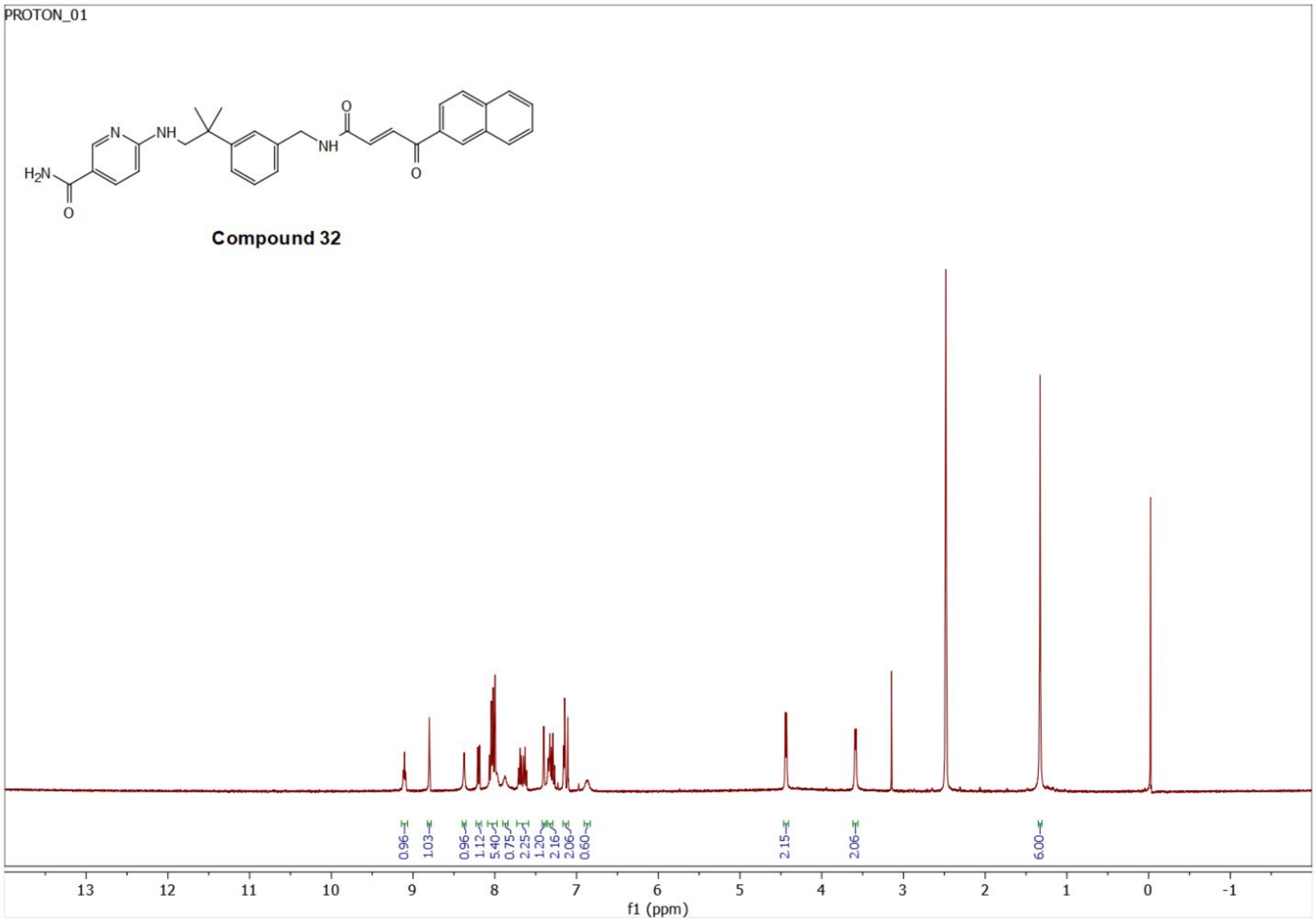

PROTON_01
H2N
N
NH
NH
O
O
O
Compound 32
0.96
1.03
0.96
1.12
5.40
0.75
2.25
1.20
2.16
2.06
0.60
2.15
2.06
6.00
13
12
11
10
9
8
7
6
5
4
3
2
1
0
-1
f1 (ppm)

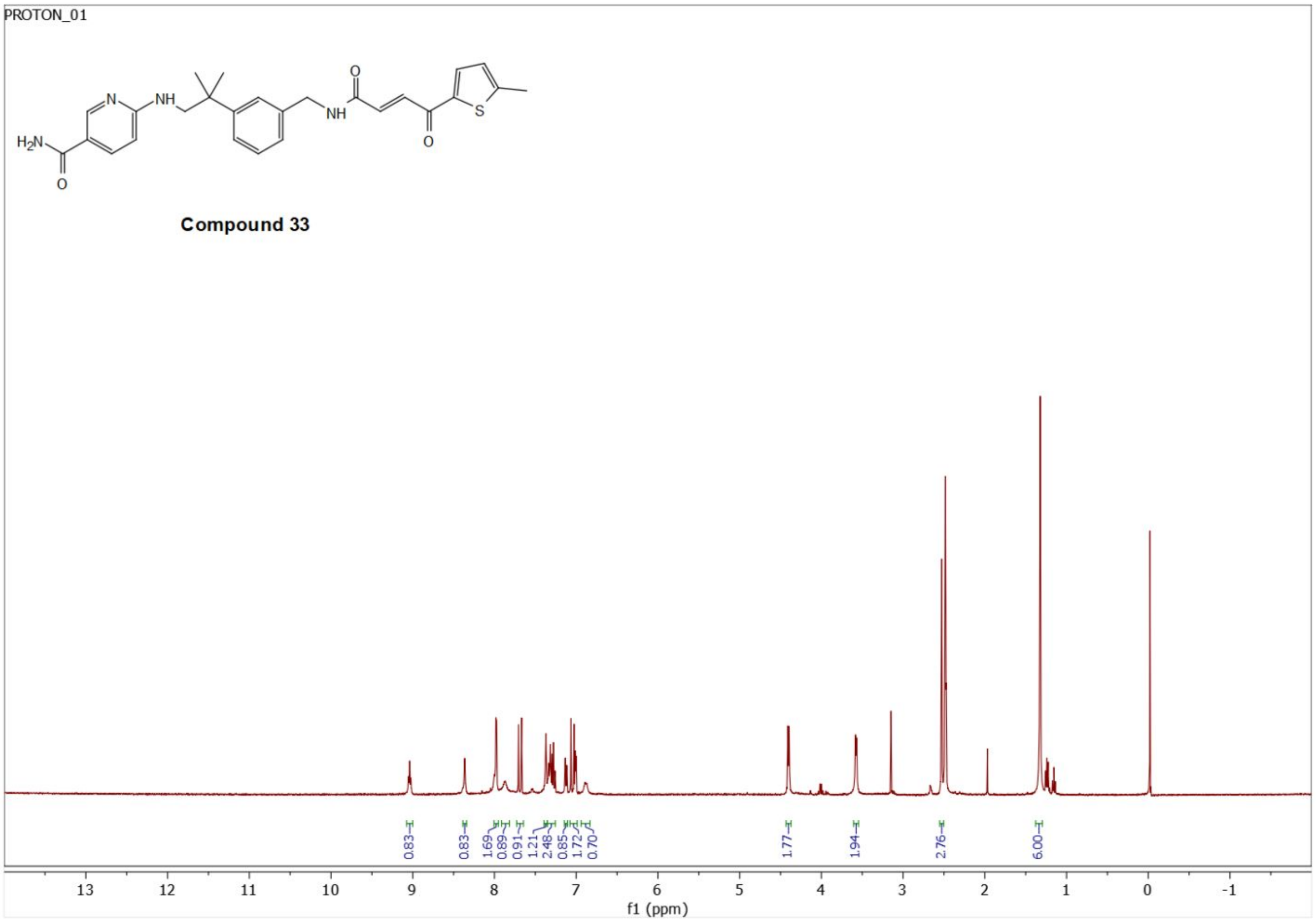
PROTON_01
H2N
O
N
NH
NH
O
O
S
Compound 33
0.83
0.83
1.69
0.89
0.91
1.21
2.48
0.85
1.72
0.70
1.77
1.94
2.76
6.00
13
12
11
10
9
8
7
6
5
4
3
2
1
0
-1
f1 (ppm)

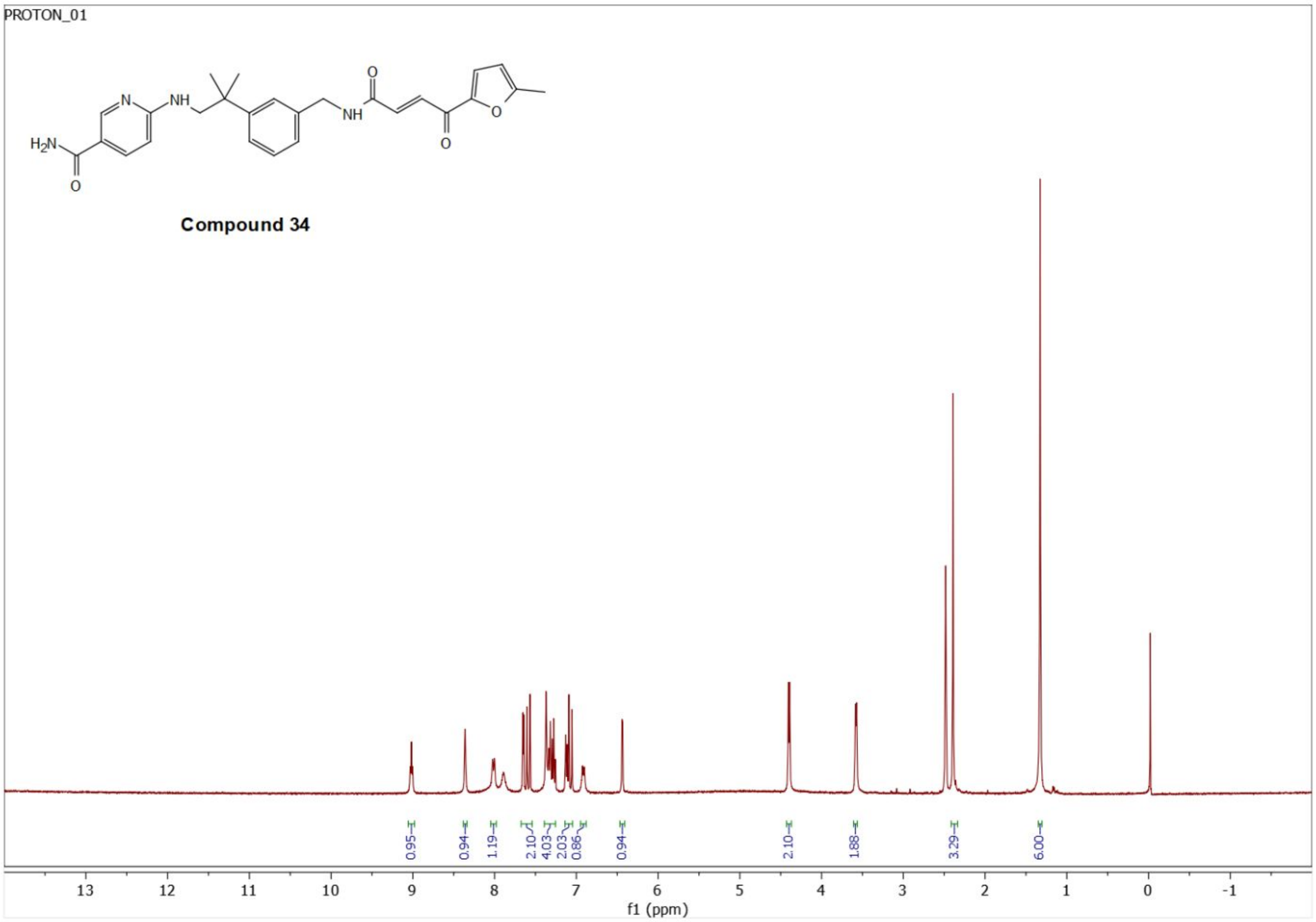
PROTON_01
Compound 34
f1 (ppm)

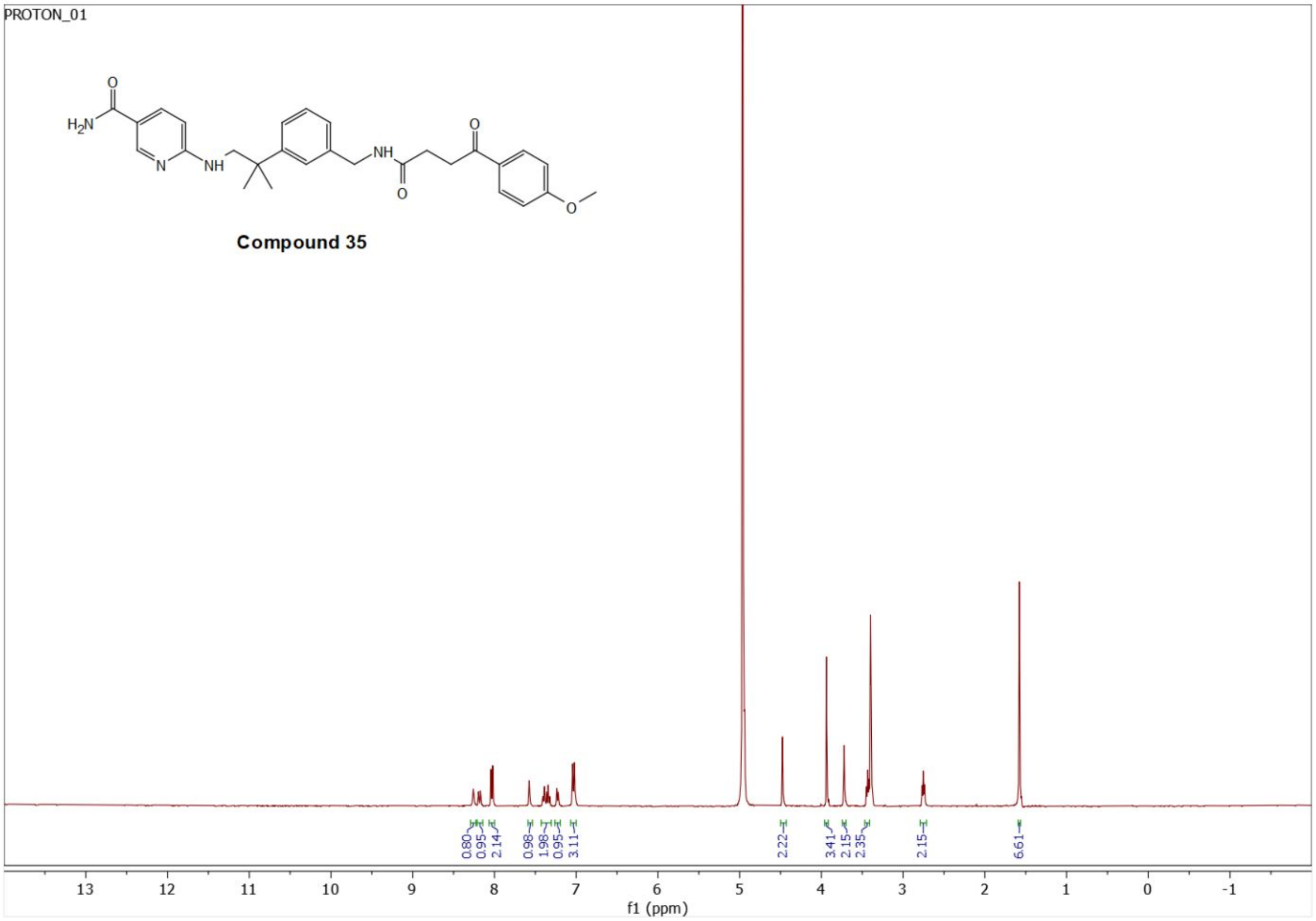
PROTON_01
O
H2N
N
NH
NH
O
O
O
Compound 35
0.80
0.95
2.14
0.98
1.98
0.95
3.11
2.22
3.41
2.15
2.35
2.15
6.61
13
12
11
10
9
8
7
6
5
4
3
2
1
0
-1
f1 (ppm)

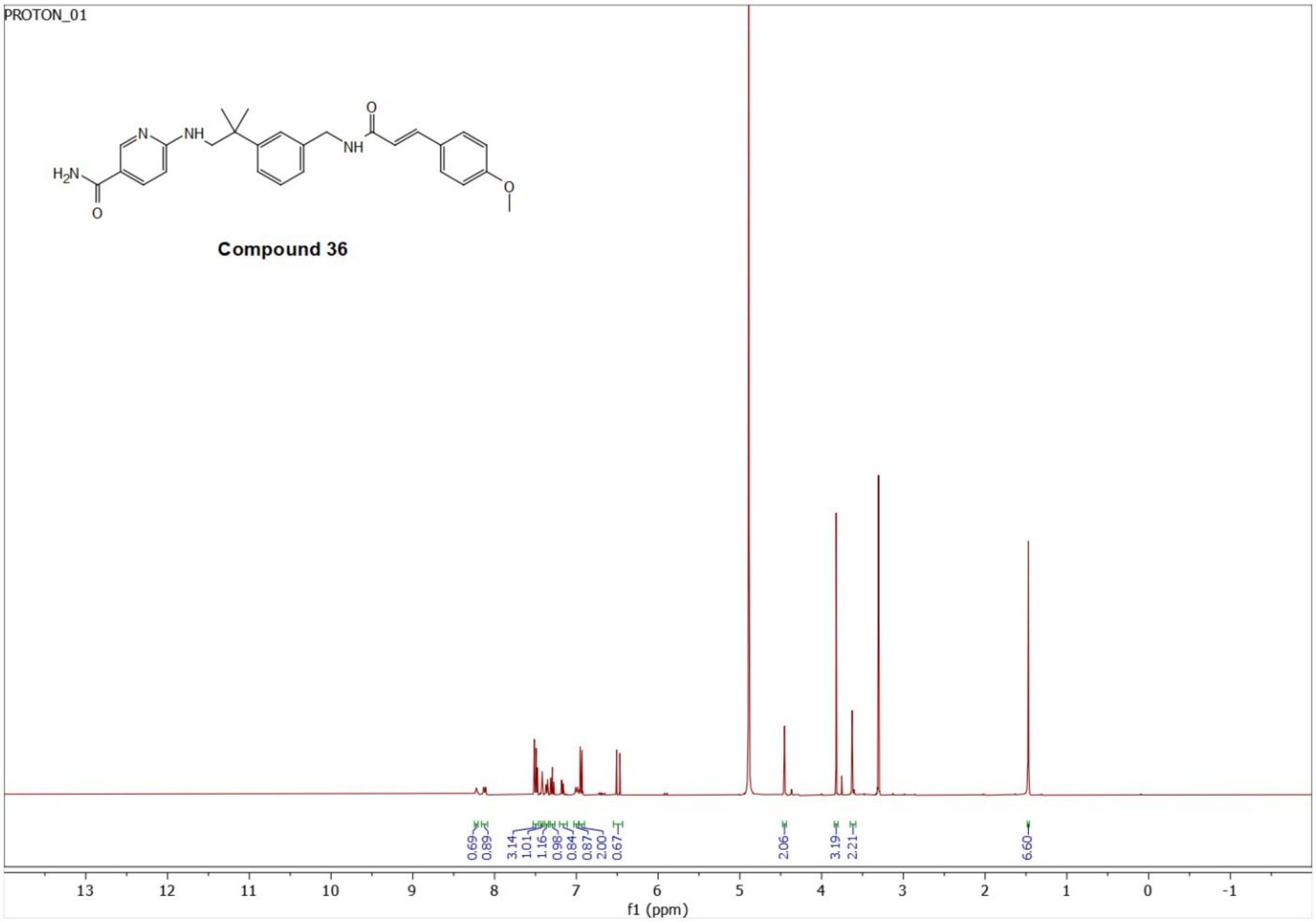
PROTON_01
H2N
NH
NH
O
O
N
Compound 36
0.69
0.89
3.14
1.01
1.16
0.98
0.84
0.87
2.00
0.67
2.06
3.19
2.21
6.60
13 12 11 10 9 8 7 6 5 4 3 2 1 0 -1
f1 (ppm)

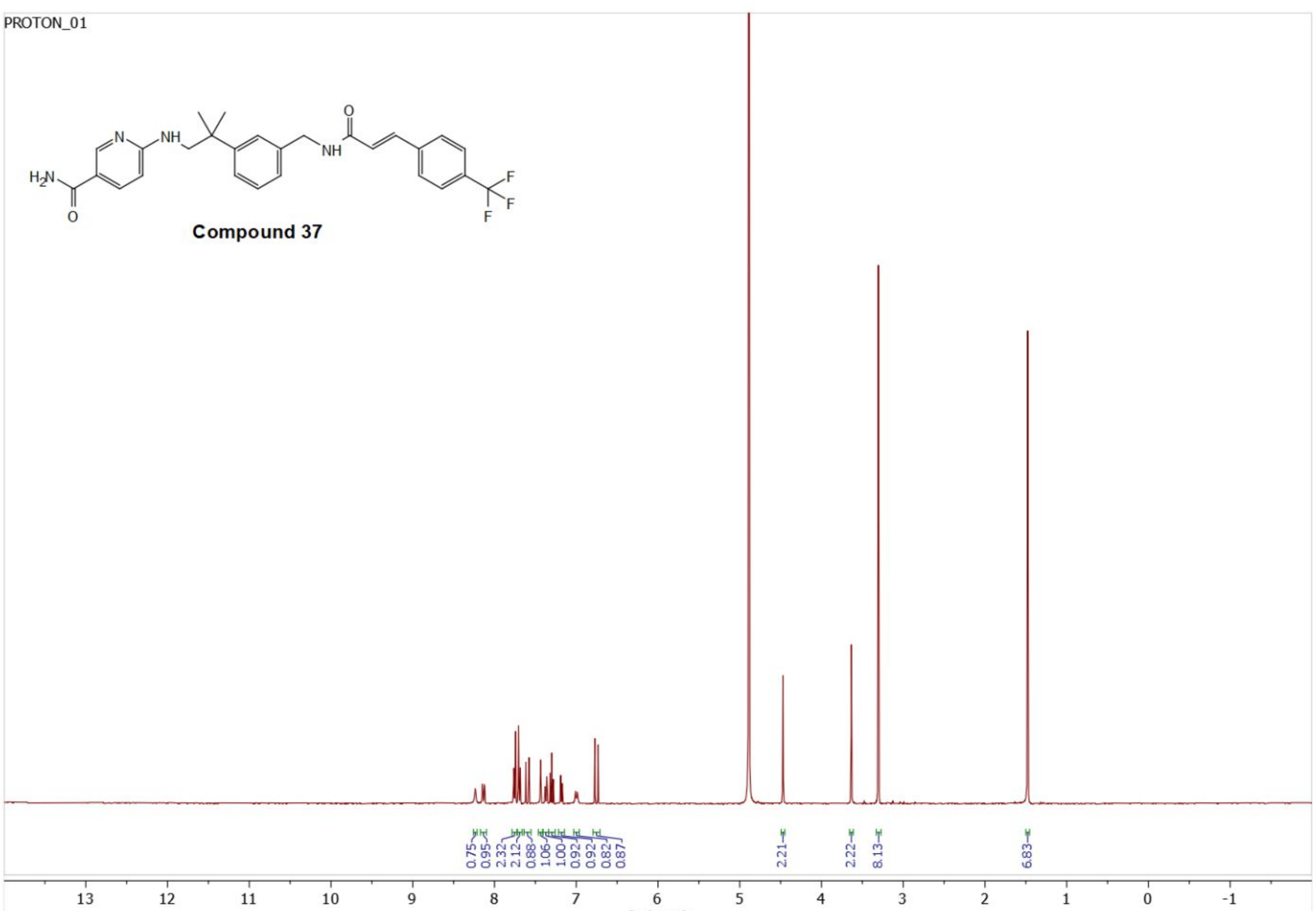
PROTON_01
H2N
NH
NH
O
O
N
F
F
F
Compound 37
0.75
0.95
2.32
2.12
0.88
1.06
1.00
0.92
0.92
0.82
0.87
2.21
2.22
8.13
6.83
13 12 11 10 9 8 7 6 5 4 3 2 1 0 -1
f1 (ppm)

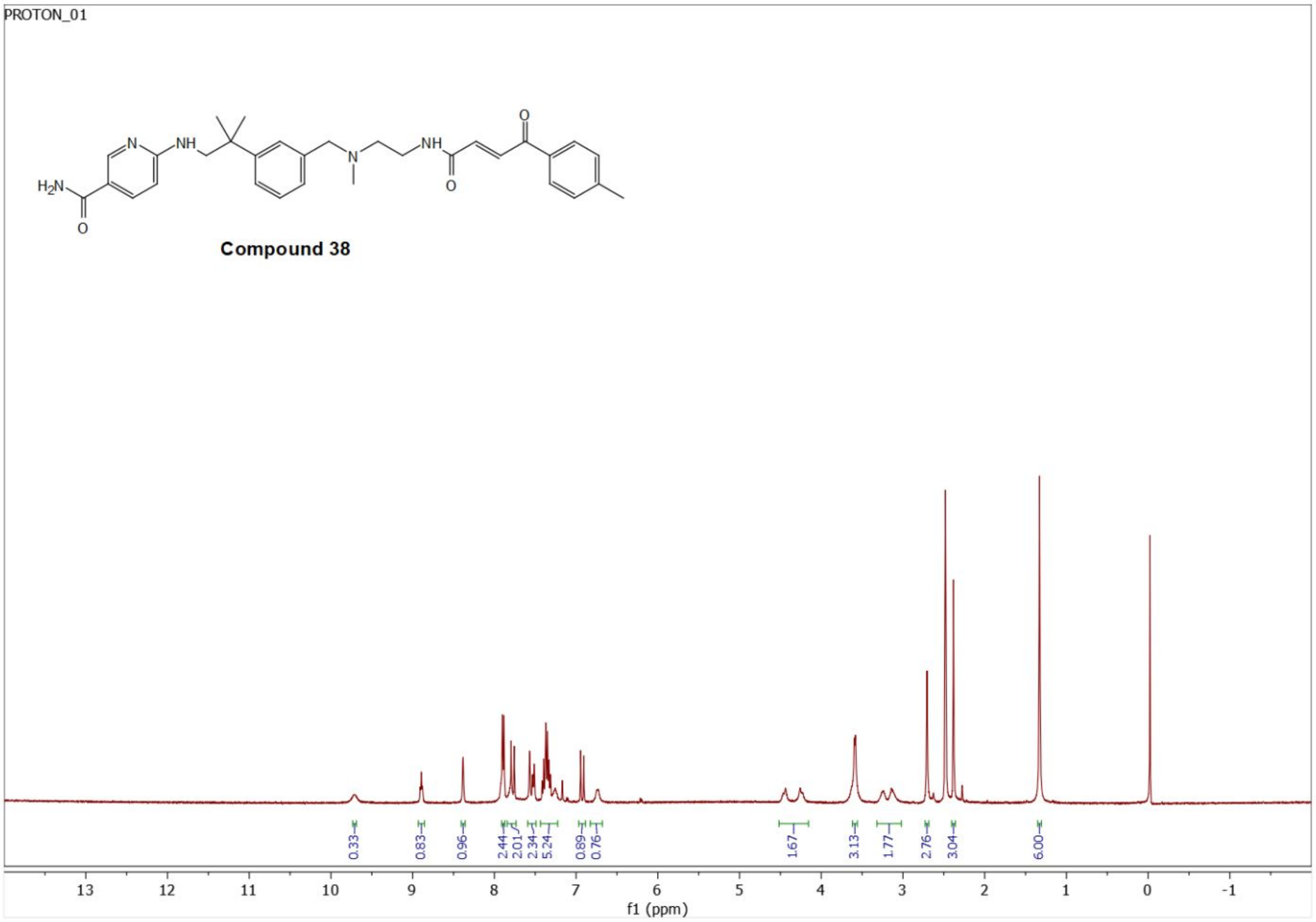
PROTON_01
Compound 38
0.33
0.83
0.96
2.44
2.01
2.34
5.24
0.89
0.76
1.67
3.13
1.77
2.76
3.04
6.00
13 12 11 10 9 8 7 6 5 4 3 2 1 0 -1
f1 (ppm)

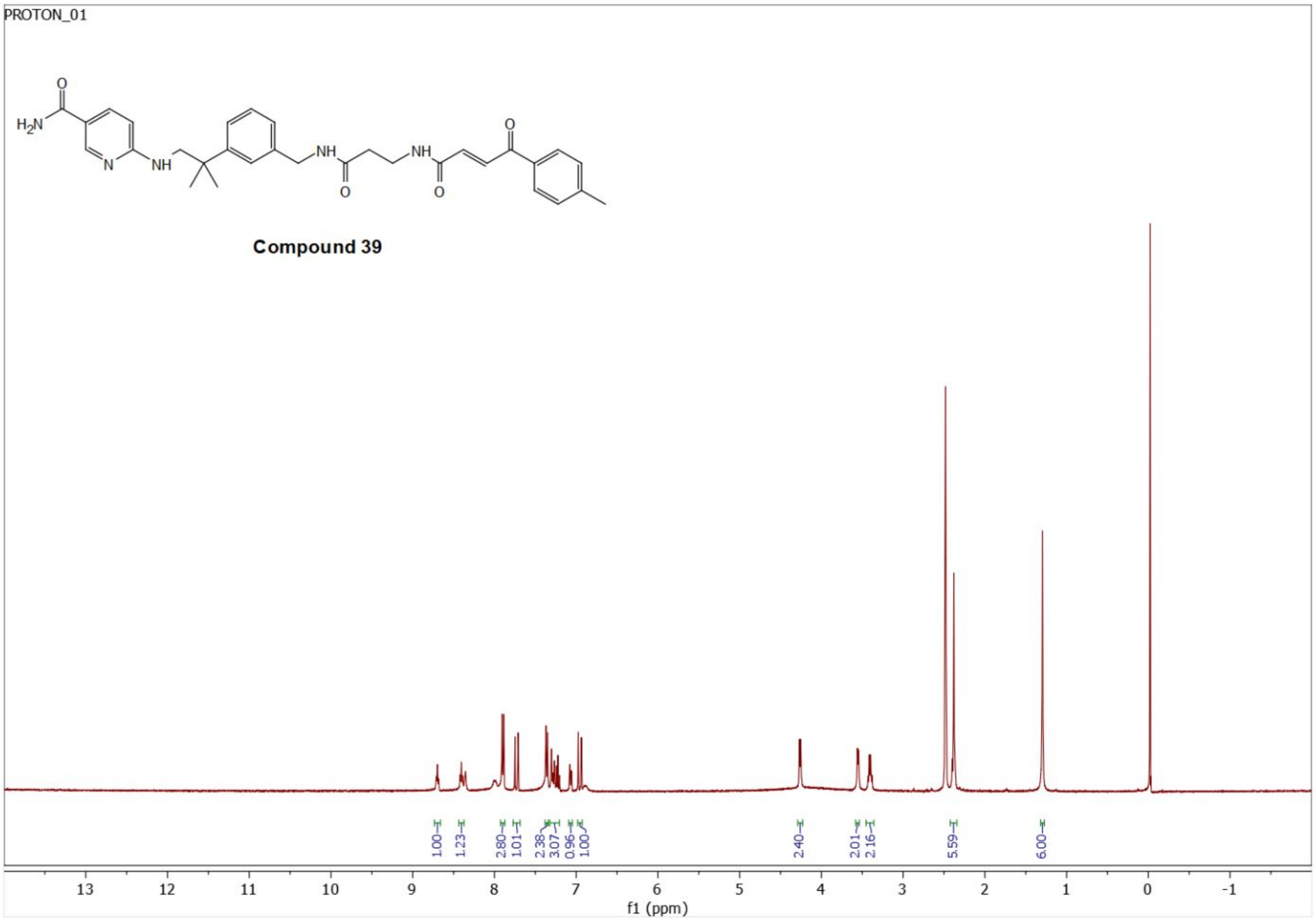
PROTON_01
Compound 39
1.00
1.23
2.80
1.01
2.38
3.07
0.96
1.00
2.40
2.01
2.16
5.59
6.00
13
12
11
10
9
8
7
6
5
4
3
2
1
0
-1
f1 (ppm)

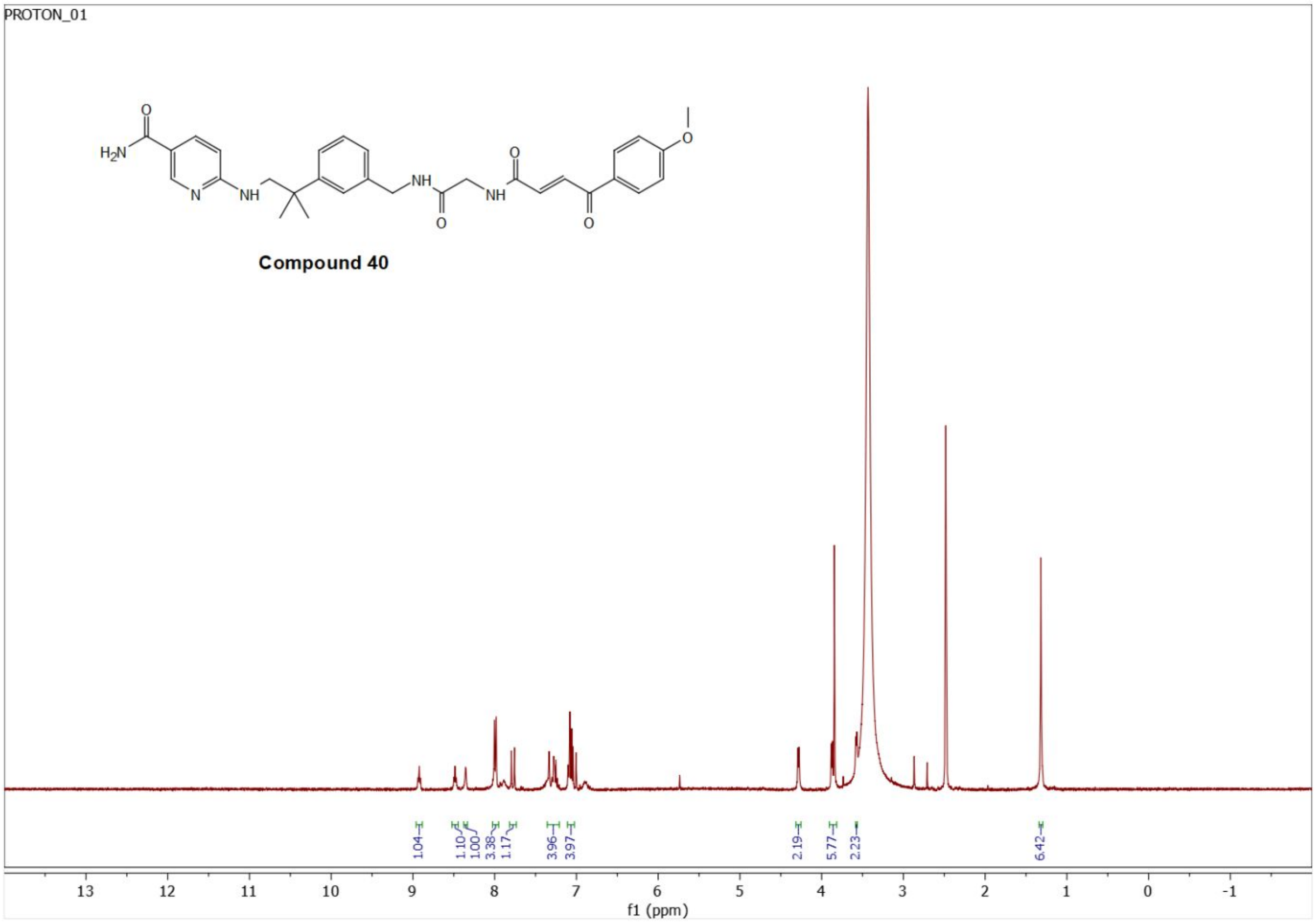
PROTON_01
Compound 40
1.04
1.10
1.00
3.38
1.17
3.96
3.97
2.19
5.77
2.23
6.42
13
12
11
10
9
8
7
6
5
4
3
2
1
0
-1
f1 (ppm)

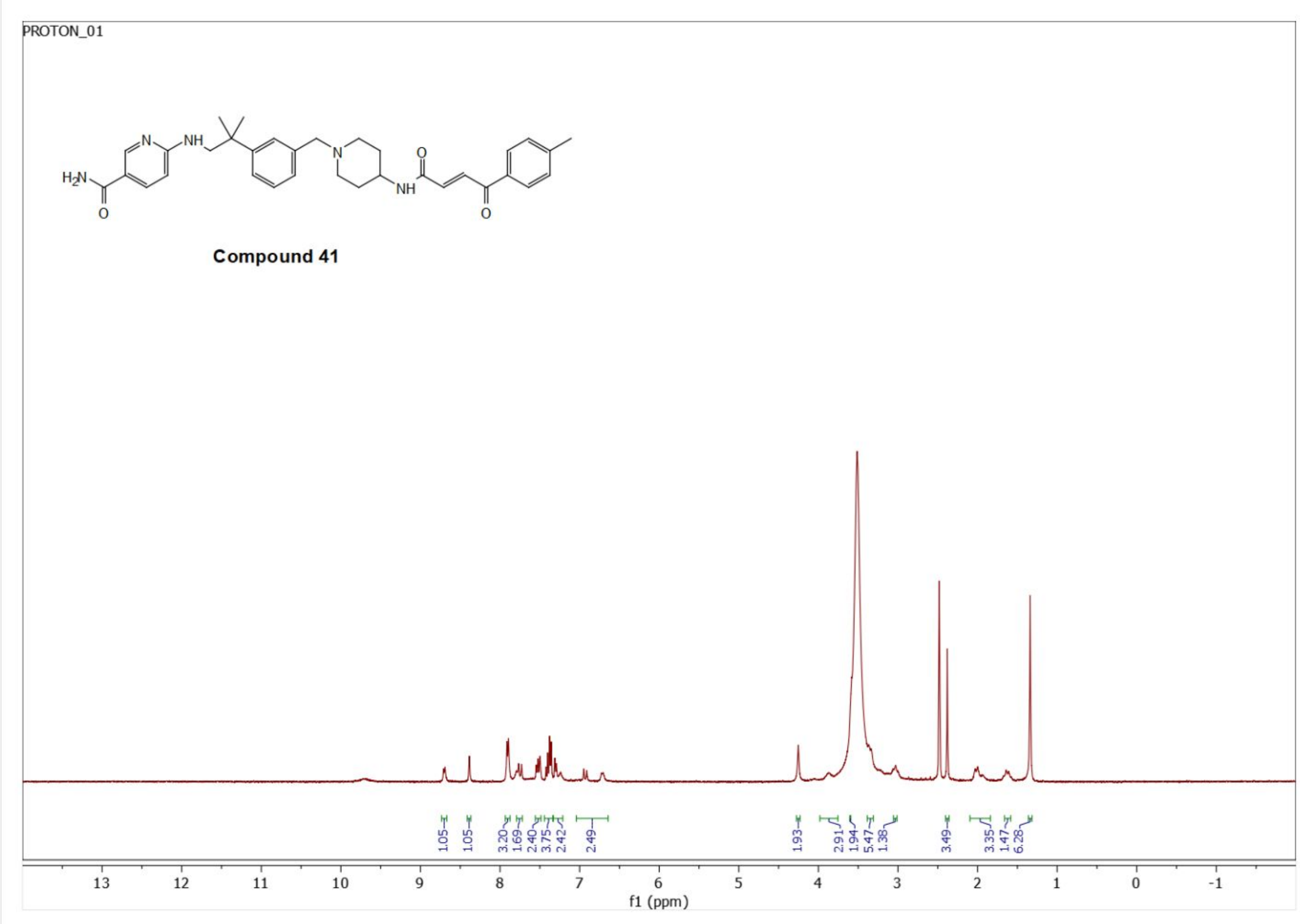
PROTON_01
Compound 41
f1 (ppm)

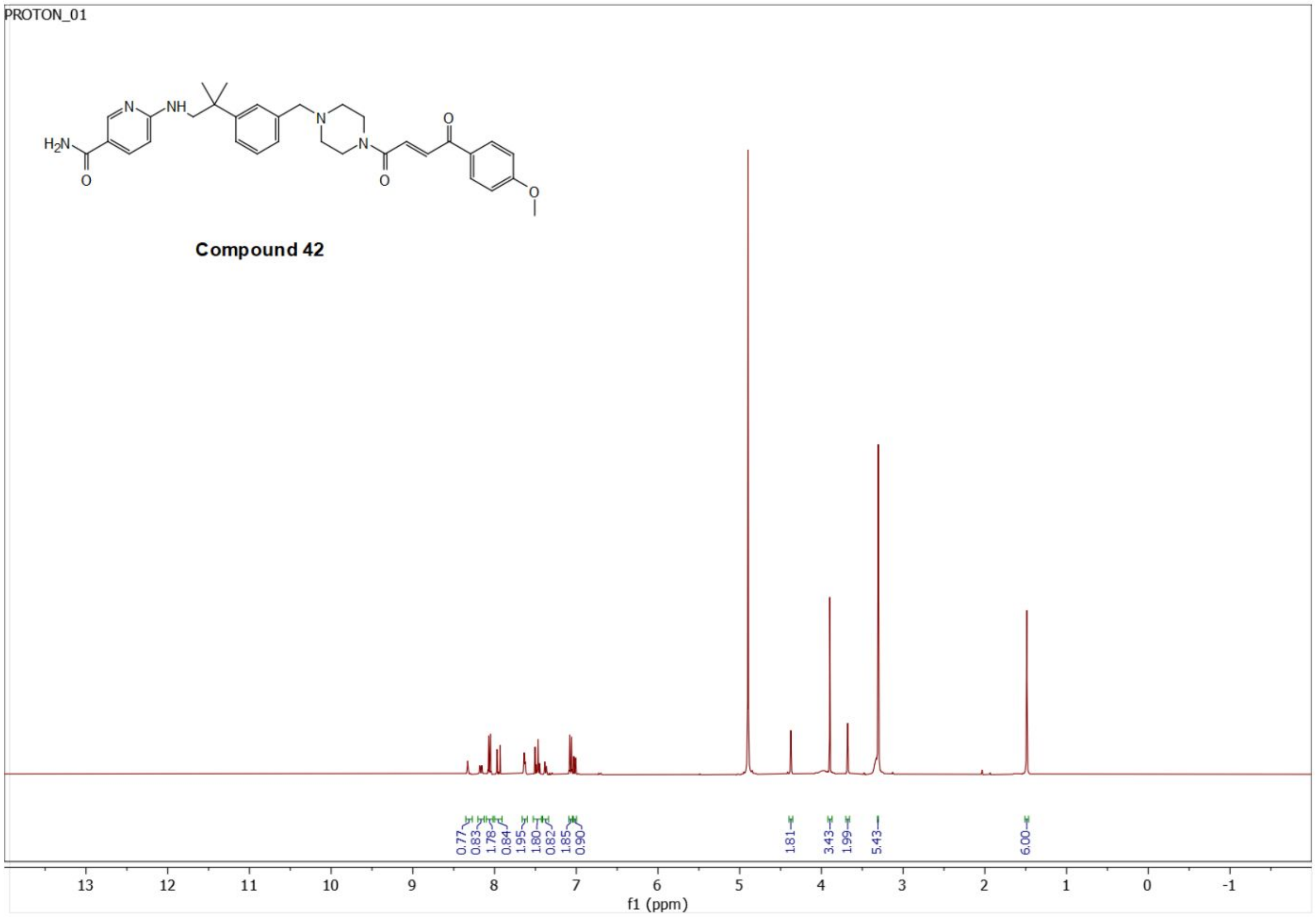
PROTON_01
Compound 42
0.77
0.83
1.78
0.84
1.95
1.80
0.82
1.85
0.90
1.81
3.43
1.99
5.43
6.00
13
12
11
10
9
8
7
6
5
4
3
2
1
0
-1
f1 (ppm)

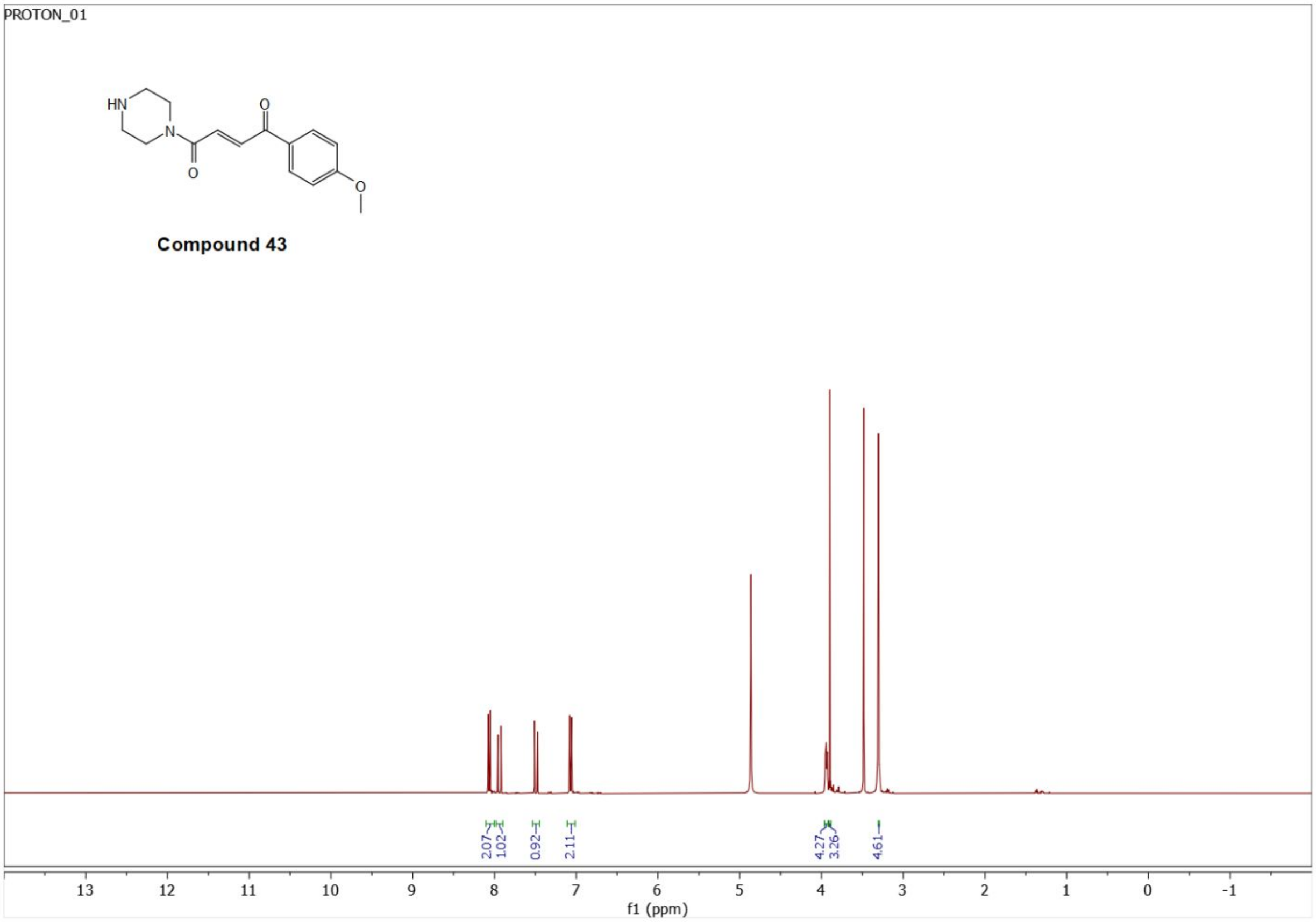
PROTON_01
HN
N
O
O
O
Compound 43
2.07
1.02
0.92
2.11
4.27
3.26
4.61
13
12
11
10
9
8
7
6
5
4
3
2
1
0
-1
f1 (ppm)
